# Baseline exposure, antibody subclass, and hepatitis B response differentially affect malaria protective immunity following RTS,S/AS01E vaccination in African children

**DOI:** 10.1186/s12916-018-1186-4

**Published:** 2018-10-31

**Authors:** Itziar Ubillos, Aintzane Ayestaran, Augusto J Nhabomba, David Dosoo, Marta Vidal, Alfons Jiménez, Chenjerai Jairoce, Hèctor Sanz, Ruth Aguilar, Nana Aba Williams, Núria Díez-Padrisa, Maximilian Mpina, Hermann Sorgho, Selidji Todagbe Agnandji, Simon Kariuki, Benjamin Mordmüller, Claudia Daubenberger, Kwaku Poku Asante, Seth Owusu-Agyei, Jahit Sacarlal, Pedro Aide, John J Aponte, Sheetij Dutta, Ben Gyan, Joseph J Campo, Clarissa Valim, Gemma Moncunill, Carlota Dobaño

**Affiliations:** 1 ISGlobal, Hospital Clínic, Universitat de Barcelona, Carrer Rosselló 153 CEK building, E-08036 Barcelona, Catalonia Spain; 2 Centro de Investigação em Saúde de Manhiça (CISM), Rua 12, Cambeve, Vila de Manhiça, CP 1929 Maputo, Mozambique; 3 Kintampo Health Research Centre, Kintampo, Ghana; 4 Spanish Consortium for Research in Epidemiology and Public Health (CIBERESP), Barcelona, Spain; 5 Ifakara Health Institute, Bagamoyo Research and Training Center, P.O. Box 74, Bagamoyo, Tanzania; 6 Institut de Recherche en Sciences de la Santé, Nanoro, Burkina Faso; 7 Centre de Recherches Médicales de Lambaréné (CERMEL), BP 242, Lambaréné, Gabon; 8 Institute of Tropical Medicine and German Center for Infection Research, University of Tübingen, Wilhelmstraße 27, 72074 Tübingen, Germany; 9 Kenya Medical Research Institute (KEMRI)/Centre for Global Health Research, Kisumu, Kenya; 10 Swiss Tropical and Public Health Institute, Socinstrasse 57, 4002 Basel, Switzerland; 11 Facultade de Medicina, Universidade Eduardo Mondlane, Maputo, Mozambique; 12 Walter Reed Army Institute of Research (WRAIR), Silver Spring, MD USA; 13 Noguchi Memorial Institute for Medical Research, University of Ghana, Accra, Ghana; 14 Department of Osteopathic Medical Specialties, Michigan State University, 909 Fee Road, Room B 309 West Fee Hall, East Lansing, MI 48824 USA; 15 Department of Immunology and Infectious Diseases, Harvard T.H. Chen School of Public Health, 675 Huntington Ave., Boston, MA 02115 USA

**Keywords:** Malaria, Vaccine, Antibody, RTS,S, *Plasmodium falciparum*, Immunogenicity, Correlate protection, African children, Hepatitis B

## Abstract

**Background:**

The RTS,S/AS01E vaccine provides partial protection against malaria in African children, but immune responses have only been partially characterized and do not reliably predict protective efficacy. We aimed to evaluate comprehensively the immunogenicity of the vaccine at peak response, the factors affecting it, and the antibodies associated with protection against clinical malaria in young African children participating in the multicenter phase 3 trial for licensure.

**Methods:**

We measured total IgM, IgG, and IgG_1–4_ subclass antibodies to three constructs of the *Plasmodium falciparum* circumsporozoite protein (CSP) and hepatitis B surface antigen (HBsAg) that are part of the RTS,S vaccine, by quantitative suspension array technology. Plasma and serum samples were analyzed in 195 infants and children from two sites in Ghana (Kintampo) and Mozambique (Manhiça) with different transmission intensities using a case-control study design. We applied regression models and machine learning techniques to analyze immunogenicity, correlates of protection, and factors affecting them.

**Results:**

RTS,S/AS01E induced IgM and IgG, predominantly IgG1 and IgG3, but also IgG2 and IgG4, subclass responses. Age, site, previous malaria episodes, and baseline characteristics including antibodies to CSP and other antigens reflecting malaria exposure and maternal IgGs, nutritional status, and hemoglobin concentration, significantly affected vaccine immunogenicity. We identified distinct signatures of malaria protection and risk in RTS,S/AS01E but not in comparator vaccinees. IgG2 and IgG4 responses to RTS,S antigens post-vaccination, and anti-CSP and anti-*P. falciparum* antibody levels pre-vaccination, were associated with malaria risk over 1-year follow-up. In contrast, antibody responses to HBsAg (all isotypes, subclasses, and timepoints) and post-vaccination IgG1 and IgG3 to CSP C-terminus and NANP were associated with protection. Age and site affected the relative contribution of responses in the correlates identified.

**Conclusions:**

Cytophilic IgG responses to the C-terminal and NANP repeat regions of CSP and anti-HBsAg antibodies induced by RTS,S/AS01E vaccination were associated with malaria protection. In contrast, higher malaria exposure at baseline and non-cytophilic IgG responses to CSP were associated with disease risk. Data provide new correlates of vaccine success and failure in African children and reveal key insights into the mode of action that can guide development of more efficacious next-generation vaccines.

**Electronic supplementary material:**

The online version of this article (10.1186/s12916-018-1186-4) contains supplementary material, which is available to authorized users.

## Background

Malaria was estimated to cause 445,000 deaths globally in 2016, mostly attributable to *Plasmodium falciparum* in African children [1]. A reduction in malaria-associated deaths and morbidity has been attained in the last years by combining malaria control interventions, such as vector-targeted measures including distribution of long-lasting insecticide-treated bednets and indoor residual spraying, as well as mass drug administration [2]. However, emerging mosquito resistance to insecticides [3], parasite resistance to drugs [4–7], and non-sustained surveillance strategies [8] can jeopardize malaria elimination strategies. Furthermore, there is a potential for a rebound of malaria illness if immunity is lost as a result of sustained elimination campaigns, in case *P. falciparum* transmission is later reintroduced [9]. In this scenario, an effective malaria vaccine remains an essential tool to reduce and sustain malaria burden at low levels and facilitate elimination [10].

RTS,S/AS01E (Mosquirix™) has consistently provided partial protection against malaria in African children, as demonstrated in phase 3 clinical trial [11–15]. RTS,S/AS01E is based on virus-like particles with the hepatitis B surface antigen (HBsAg) and a fragment of the *P. falciparum* circumsporozoite protein (CSP), which comprises the central repeat region (NANP)_n_ and the C-terminus (C-term) [16], formulated with GlaxoSmithKline’s proprietary adjuvant AS01. RTS,S/AS01E generates high IgG titers to the CSP NANP immunodominant B cell epitope that remains above naturally acquired titers for years [17]. However, such antibodies have not consistently correlated with protection across all ages and malaria endpoints [14, 18]. Since only total IgG responses to NANP have been measured in field trials thus far, it is possible that (i) other CSP antigenic epitopes are targets of vaccine-induced protective immunity and (ii) the isotype/subclass balance, important for effector function of antibodies, is more relevant in protection against malaria than the magnitude of the IgG response. Binding kinetics of anti-NANP antibodies has been analyzed in pilot experiments and showed no strong association with protection in African children, although longitudinal analyses indicated an effect of previous exposure to vaccine or natural infection [19, 20] that can be mimicked by fractional last doses [21]. This shows that it is crucial to understand how RTS,S immunogenicity and vaccine efficacy are affected by baseline factors like age at first vaccination, sex, maternal antibodies, and malaria transmission intensity (MTI) [22]. Efforts to improve RTS,S efficacy to rationally develop and deploy second-generation vaccines should rely on a better understanding of its mode of action, currently unknown, to unravel why RTS,S does not prevent a higher proportion of malaria episodes in African children and what are the factors affecting this.

In this study, we set out to characterize in detail the immunogenicity of the RTS,S/AS01E vaccine in the African pediatric multicenter phase 3 clinical trial and assess the association between the fine epitope specificity and isotype/subclass of the antibody response and protection against clinical malaria. We measured total IgG and, for the first time, IgM as well as IgG1, IgG2, IgG3, and IgG4 responses to the HBsAg and to three CSP constructs: a full-length (FL) and two truncated proteins, one with the central NANP_16_ repeat region and the other with the C-term region [16]. In addition, we investigated the effect of baseline variables including age, malaria exposure, maternal antibodies, sex, and nutritional status on immunogenicity and protection. By applying regression models and machine learning techniques, we identified novel antibody signatures at baseline and induced by RTS,S/AS01E vaccination that correlated with protection and risk from clinical malaria, defined their antigen targets and Ig isotypes/subclasses, and assessed their determinants.

## Methods

### Design

This study was carried out in two of the seven sites included in the multicenter immunology study MAL067, ancillary to the phase 3 randomized clinical trial MAL055 (NCT00866619)—Kintampo in Ghana (representative of moderate-high MTI) and Manhiça in Mozambique (representative of low MTI) [18], to be able to assess the effect of MTI on vaccine responses. These two sites were prioritized due to higher availability of sufficient numbers and volumes of samples from both study visits and age cohorts. Subjects were followed up by passive case detection (PCD) starting 14 days after sample collection at month (M) 3, approximately 44 days after the third dose (M2), for the subsequent 12 months, when they were censored.

**Fig. 1:**
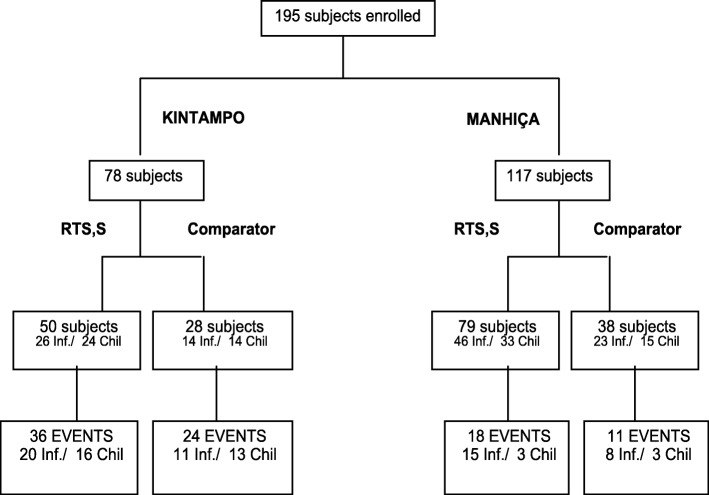
Flowchart of the study population. Inf. infants, Chil children, EVENTS events of clinical malaria

Children age 5–17 months and infants age 6–12 weeks with ≥ 150 μL plasma/serum samples available at M0 (baseline) and M3 were selected. We included 129 RTS,S/AS01E-vaccinated and 66 comparator-vaccinated children and infants from both sites (Fig. 1, Additional file 1: Table S1). For the correlates of malaria disease protection and risk analysis, 78 children and infants were randomly selected from Kintampo, 117 participants were selected from Manhiça according to a prior case-control study of cellular markers [23], and all were analyzed in a case-control design.

### Antibody assays

Quantitative suspension array technology (qSAT) [24, 25] was used to measure antibody responses to three CSP constructs (FL, NANP repeat, and C-term [residues 274 to 387: KNNQG...SSIGL] recombinant proteins from WRAIR) and HBsAg (Abcam). The qSAT assays applied the xMAP™ technology (Luminex Corp., TX) and were previously standardized and optimized to control for sources of variability [26, 27]. The multiplex antigen panel also included 32 *P. falciparum* proteins [26, 27] analyzed as markers of malaria exposure and maternally transferred antibodies (see below). Briefly, antigen-coupled multiplex beads were mixed with 50 μL of test sample, negative or positive control [28, 29] at multiple dilutions (see Additional file 1: Supplementary methods). After incubation and washing, biotinylated secondary antibodies were added. Following streptavidin-R-phycoerythrin incubations, samples were acquired with a Luminex 100/200 analyzer and antibody levels measured as median fluorescence intensity (MFI). Data pre-processing is detailed in Additional file 1: Supplementary methods.

### Statistical analysis

RTS,S/AS01E immunogenicity was evaluated for all antigens/Ig using basic descriptive methods (see Additional file 1: Supplementary methods) and longitudinal linear mixed effects models [30] including vaccination, visit (M0, M3) and the interaction between them, and adjusting by site. The effect of the vaccine on Ig responses at M3, and on change on Ig levels from M0 to M3, was assessed through tests of the corresponding fixed effects. All models included a random intercept for the individual and a random slope for changes over time among predictors (see Additional file 1: Supplementary methods). To evaluate more thoroughly the impact of age on post-vaccination levels and association between pre- and post-vaccination in RTS,S vaccinees, mixed effects models adjusted by site were also estimated across age cohorts (children and infants).

To understand the effect of the remaining study covariates on M3 Ig levels to all antigens, we fitted first univariate and next multivariable linear regression models (coefficient, 95% confidence intervals (CI), adjusted *p* values) including only RTS,S vaccinees, with the following predictors: sex, malaria transmission season at M3, having clinical malaria episodes between M0 and M3, and baseline variables like age (in weeks), antibody levels, hemoglobin (Hb) concentrations, weight-for-age Z score (WAZ), and height-for-age Z score (HAZ). For comparison purposes, models were also fitted at pre-vaccination and on comparator vaccinees at M3. Malaria transmission season was defined as high between April–October for Kintampo and November–April for Manhiça; the remaining months were defined as low transmission. The effect of baseline antibody levels was evaluated in three different ways. First, using the same antigen/Ig as the outcome variable at M3. Second, a *P. falciparum* exposure index was defined as follows [27, 31]. Upon examination of antibody responses to the 32 antigen panel [26, 27], we selected 28 markers in which IgM responses were M3 > M0 and thus acquired with age (e.g., children > infants) and exposure (e.g., Kintampo > Manhiça). Principal component analysis (PCA) of IgM responses to these antigens was performed to construct the corresponding variables, and the first component (PC1) that explained 63% of the variability was selected. Third, a *P. falciparum* maternal antibody index was defined in subjects < 10 months of age [27, 31]. For this, 17 antigens including two VAR2CSA pregnancy-specific antigen constructs were selected which IgG responses were M0 > M3 and thus declined with age (e.g., infants > children) and were higher in infants from the high MTI site (e.g., Kintampo > Manhiça). The construction of the maternal score was done in the same way as the exposure score. We selected the first component that explained 54% of the variability. Linearity of the associations with continuous covariates was evaluated through penalized splines in generalized additive models (GAM); variables were modeled as linear. A stepwise algorithm was used in multivariable models.

Analysis of correlates of protection was based on the case-control design. The outcome was clinical malaria detected by PCD defined by fever > 37.5 °C with any parasitemia in the 12 months after the start of follow-up (M3 plus 14 days). Logistic regression models (odds ratio (OR), 95% CI, adjusted *p* values) were fitted first univariate and next multivariable to obtain the effect of different predictors in the odds of having malaria. Main predictors included levels (log_10_MFI) of antibodies at M3, increment of antibody levels between M0 and M3, and ratios (IgG1 + IgG2)/(IgG2 + IgG4) at M3, in RTS,S vaccinees (comparators modeled separately for comparison purposes). The impact of the other covariates (same as above) on the association between antibody responses and clinical malaria risk/protection was also assessed. The linearity of the log_10_-transformed antibody levels was evaluated when the outcome was case-control. Univariate models were adjusted by site.

Next, multivariable models were obtained in RTS,S-vaccinated subjects through the stepwise algorithm, R package *MASS*, and function *stepAIC*. Both backwards and forward methods were combined to obtain the model with the minimum Akaike information criterion (AIC). All potential single variables were proposed in the first step of the model, not only the significant ones. For the assessment of the maternal index, only subjects < 10 months were taken into account. Correction for multiple testing was done by Holm [32] when analyzing IgG and IgM (with the following predictors: M3 antibody levels and M0–M3 change in antibodies) and IgG subclass ratios. Benjamini-Hochberg [33] was used when IgG_1–4_ subclass levels were the predictors. Holm was used to control for family wise error when there were few tests, whereas Benjamini-Hochberg was used to control for the false discovery rate when there were more tests (e.g., IgG_1–4_). HBsAg was analyzed separately from CSP constructs.

Finally, to identify the most relevant antibody variables associated with clinical malaria in multi-marker analysis, three machine learning algorithms were computed: (i) elastic net, which is a shrinkage regression that simultaneously does automatic variable selection and continuous shrinkage and can select groups of correlated variables; (ii) recursive feature elimination algorithm using support vector machines (SVM) [34] with linear kernel, which recursively removes features of low importance (computed using the weights of the linear SVM); and (iii) random forest, which constructs a multitude of uncorrelated decision trees and defines the importance of each variable by applying the permutation of the variable’s values approach [35]. The optimal tuning parameter values for each machine learning method, i.e., *α* and *λ* penalty parameters of elastic net, cost parameter of linear SVM, and number of trees and number of variables to be included in each iteration of random forest, were computed using a 5-fold cross-validation approach [36].

## Results

### RTS,S/AS01E vaccine immunogenicity

**Fig. 2:**
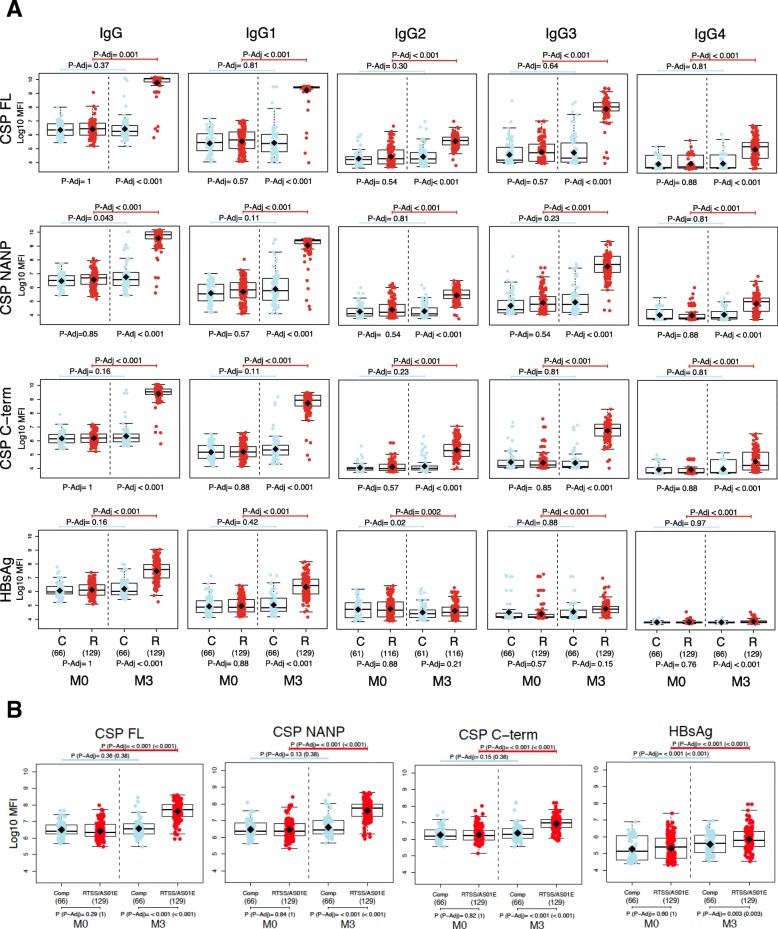
RTS,S/AS01E vaccine immunogenicity: CSP and HBsAg antibody responses in RTS,S and comparator vaccinees comparing pre- and post-vaccination. **a** IgG antibody levels (*R* = RTS,S; *C* = comparators). **b** IgM antibody levels. Groups were compared through *t* tests and *p* values adjusted by Holm for IgG and IgM and by Benjamini-Hochberg for IgG_1–4_, as explained in the “Methods” section

Three doses of RTS,S/AS01E vaccination at 1-month intervals induced a highly statistically significant increase in antibody levels (log_10_MFI) to CSP FL, NANP repeat, and C-term antigens and to HBsAg, from baseline (M0) to 1 month after the third dose (M3) for all Ig isotypes and subclasses (*p* <  0.001) (Fig. 2). Comparisons of the magnitude of antibody responses to the RTS,S antigens between the various isotypes/subclasses, and the correlations among them, are shown in Additional file 1: Supplementary results Table S2. Fold changes in RTS,S-specific antibody levels at post-vaccination compared to baseline were evaluated with mixed models (Additional file 1: Table S3). Overall differences between pre- and post-vaccination conferred by RTS,S were significant; for IgG, IgG1, and IgG3, they were substantially higher than for IgG2 and IgG4; changes for HBsAg were lesser than those for CSP. Comparator vaccinees had no or negligible increases in responses from pre- to post-vaccination. When contrasting RTS,S vs comparators at post-vaccination, the highest ratios were again recorded for IgG and IgG1 and the lowest for IgM, followed by IgG4 and IgG2 (Additional file 1: Figure S4).

#### Effect of age

Children vaccinated with RTS,S/AS01E had significantly higher levels of IgG to all CSP constructs, and of IgG1 and IgG3 (but not other isotypes/subclasses) to NANP and C-term, than infants at M3 (Additional file 1: Figure S5). Levels of antibodies to HBsAg at M3 were also higher in RTS,S-vaccinated children than infants, except for IgG2. Regarding changes in antibody levels from pre- to post-vaccination, children had a greater increase to all RTS,S antigens than infants, particularly for CSP FL (all isotypes and subclasses) (Additional file 1: Table S3 and Figure S6). Statistically significant or marginally significant differences between infants and children were recorded in IgG (CSP FL, C-term, and NANP), IgG1 (CSP FL), IgG2 (CSP FL and NANP), and IgG3 (CSP FL, C-term, and NANP). For HBsAg, RTS,S-vaccinated children had significantly higher pre- to post-vaccination changes for IgG, IgM, and IgG1 than infants. Ratios of antibody levels in RTS,S to comparators at post-vaccination in infants and children are reported in Additional file 1: Figure S7. Using univariate linear models adjusted by site, we evaluated whether Ig levels induced by RTS,S vaccination changed with age when computed as a continuous variable within infant and children cohorts (Additional file 1: Table S4). We found that for HBsAg (but not CSP), log_10_MFIs for most antibodies significantly increased for every 1-week increase in age at baseline in infants and children. Antibody seropositivity was consistent between age groups except for HBsAg (Additional file 1: Table S5).

#### Effect of site

Levels of IgG, IgG1, and IgG3 to CSP or HBsAg in RTS,S vaccinees at M3 were not different in the two sites (Additional file 1: Figures S8 and S9 stratified also by age), whereas levels of IgG2 to all CSP constructs and IgG4 to CSP FL were significantly higher in Kintampo (high MTI) than Manhiça (low MTI). Levels of anti-CSP IgM at M3 did not differ by site, but anti-HBsAg IgMs and IgG4 were higher in Manhiça than Kintampo (Additional file 1: Table S6). Some associations between prior clinical malaria episodes or between malaria transmission season at M3 and antibody responses in univariate analysis were lost when adjusted by site (data not shown).

#### Effect of baseline CSP and HBsAg antibodies

Anti-CSP IgG levels at M0 were usually higher in infants than children but not statistically significant with this limited sample size (Additional file 1: Figure S6). Significantly higher IgM and lower IgG2 to HBsAg were detected in children, who had been previously vaccinated with the pentavalent DTwP-HBV-Hib vaccine (as part of the routine expanded program of immunization (EPI)) compared to infants (*p* <  0.001). Consistent with higher MTI, levels of IgG, IgG1, IgG2, and IgG3 to CSP constructs (and of IgM in children) were higher in Kintampo than Manhiça at baseline, but this did not happen for antibodies against HBsAg (Additional file 1: Table S7). In fact, pre-vaccination levels of anti-HBsAg IgG2, and of IgG4 in children, were significantly higher in Manhiça than in Kintampo.

**Fig. 3:**
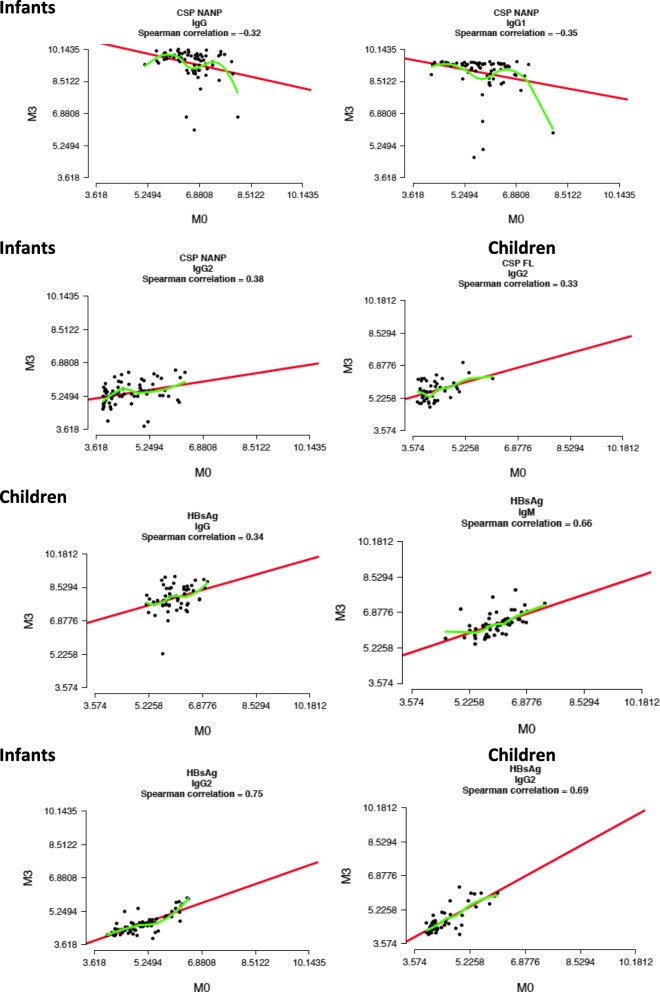
Effect of pre-vaccination (M0) antibody levels on post-vaccination (M3) RTS,S immunogenicity. Some representative examples of antibody isotypes/subclasses and antigens are shown. Spearman correlation coefficients are included as well as linear regression and non-parametric LOESS estimations, as red and green lines, respectively. See Additional file 1: Figure S6 for further details

We tested whether pre-vaccination levels had an impact on post-RTS,S vaccination responses by fitting regression models in each age group [18] and found no consistent associations with anti-CSP antibodies. Correlation coefficients were low and with varying direction, although a trend (*p* = 0.05) was observed for CSP NANP whereby infants with higher M0 had lower M3 IgG and IgG1 levels (Additional file 1: Figure S6, Fig. 3), but no effect was detected in children, except for CSP FL IgG2. However, there was a significant effect for HBsAg whereby children with higher M0 IgG, IgM, and IgG2 (also for infants) had higher M3 levels. There was no significant correlation between baseline HBsAg antibodies and CSP responses at M3, or the opposite (data not shown).

#### Effect of baseline maternal *P. falciparum* antibodies

Summary scores based on IgGs to malarial antigens at baseline in subjects younger than 10 months are shown on Additional file 1: Table S8 and Figure S10. At M0, there was a significant positive association between the maternal malaria antibody index and anti-CSP IgGs, but this was not the case for CSP IgM or for anti-HBsAg antibodies, except IgG2 (Additional file 1: Table S9). In fact, there was a significant negative association with anti-HBsAg IgM (also in comparator vaccines at M3) and IgG3 levels at baseline and a positive association for IgG2. Regarding the impact on M3 RTS,S responses, maternal antibodies were significantly negatively associated with IgG and IgG1 HBsAg levels (borderline associations to some CSP constructs) (Additional file 1: Table S9).

#### Effect of baseline *P. falciparum* exposure antibodies

Summary scores based on naturally acquired antimalarial IgM (to distinguish from maternally derived IgG) are shown in Additional file 1: Table S8 and Figure S10. At M0, there was a significant positive association between malaria exposure and IgMs to all CSPs and HBsAg (Additional file 1: Table S10). When stratifying by age, the association with HBsAg IgM was only significant in infants (data not shown). Regarding the impact on M3 responses to RTS,S, the malaria exposure index was significantly and positively associated with IgG, IgG1, and IgM (borderline for IgG3) HBsAg levels, also in comparator vaccinees for IgM (Additional file 1: Table S10).

#### Multivariable linear regression analysis of immunogenicity

Factors significantly affecting the magnitude of RTS,S antibody responses at M3 depending on the antigen/Ig are shown in Table 1. Whilst all Ig tended to increase with age, C-term and FL (but not NANP) CSP IgG2 levels decreased in subjects younger than 10 months (Additional file 1: Table S11). Site strongly affected HBsAg antibody levels, not only for IgM but also for IgGs (Table 1), particularly in subjects of age < 10 months (Additional file 1: Table S11), which were higher in Manhiça than Kintampo, as well as IgG2 and IgG4 to CSPs, which were higher in Kintampo than Manhiça. Here, prior malaria episodes (rather than exposure index) were significantly associated with higher HBsAg IgG, IgG1, and IgM, and CSP (NANP and C-term) IgG4, M3 levels. Furthermore, a statistically significant negative impact of high baseline NANP (borderline for C-term) CSP IgG on M3 responses emerged, and the positive effect of baseline HBsAg IgG2 and IgM remained. Higher anti-*P. falciparum* maternal IgG at M0 was also inversely associated with CSP (not HBsAg) IgG levels at M3 in subjects age < 10-month old (Additional file 1: Table S11).

**Table 1:** Factors affecting the immunogenicity of RTS,S/AS01E. Multivariable linear models including RTS,S/AS01E vaccinees at month 3

Isotype	Antigen	Age*	Site	Prior episode^†^	Season	Baseline Ig	Exposure index	Sex	Hb	WAZ	HAZ
IgG	CSP FL	1.21 (0;2.43), *0.05*									
	CSP C-term	1.1 (0.01;2.2), *0.048*		99.08 (− 16.6;375.18), 0.12		− 43.99 (− 69.72;3.61), 0.06			− 13.8 (− 28.2;3.5), 0.11		
	CSP NANP	1.55 (0.3;2.82), *0.02*				− 46.15 (− 65.99;-14.73), *0.009*			− 14.99 (− 30.87;4.5), 0.12		
	HBsAg	3.97 (2.83;5.12), < *0.001*	93.14 (9.18;241.67), *0.02*	238.29 (29.17;785.95), *0.01*					− 18.02 (− 32.07;− 1.06), *0.04*		
IgG1	CSP FL									− 21.26 (− 41.39;5.8), 0.11	
	CSP C-term	1.54 (0.17;2.94), *0.03*	91.17 (− 7.67;295.82), 0.08	166.93 (− 18.55;774.83), 0.1						− 21.42 (− 42.4;7.22), 0.13	
	CSP NANP					− 44.3 (− 63.7;-14.7), 0.08	8.62 (0.4;17.5), *0.04*				
	HBsAg	4.28 (3.09;5.49), < *0.001*	90.61 (4.9;246.35), *0.03*	161.88 (− 4.42;617.53), *0.06*					− 17.52 (− 32.3;0.43), *0.005*		
IgG2	CSP FL		− 69.3 (− 78.86;-55.43), < *0.001*				3.28 (− 7.2;0.8), 0.11				
	CSP C-term		− 74.77 (− 84.4;-59.2), < *0.001*								
	CSP NANP	1.2 (0.25;2.16), *0.01*	− 45.45 (− 66.66;− 10.72), *0.02*			30.86 (− 8.56;87.27), 0.14	− 5.93 (− 10.55;-1.07), *0.02*	25.18 (− 6.89;68.29), 0.14		− 24.23 (− 37.63;− 7.94), *0.006*	
	HBsAg	2.45 (1.75;3.15), < *0.001*				318.44 (234.5;423), < *0.001*					
IgG3	CSP FL			144.38 (− 20.29;649.19), 0.12						− 22.63 (− 43.2;5.31), 0.1	
	CSP C-term	1.05 (− 0.17;2.29), 0.09					5.84 (− 0.92;13.06), 0.09				
	CSP NANP	1.38 (− 0.13;2.91), 0.07		197.82 (− 8.62;870.67), 0.07					− 23.08 (− 40.14;− 1.15), *0.041*		
	HBsAg	1.96 (1.23;2.71), < *0.001*									20.89 (3.3;41.43), *0.002*
IgG4	CSP FL		− 52.07 (− 75.66;− 5.61), *0.03*	123.51 (− 21.64;537.55), 0.13			8.6 (1.45;16.2), *0.02*				− 22.32 (− 40.8;2.03), 0.07
	CSP C-term	0.93 (− 0.38;2.25), 0.16	72.93 (− 13.56;245.99), 0.12	215.08 (0.49;887.87), *0.049*		− 49.59 (− 76.34;7.39), 0.08				− 26.52 (− 45.39;− 1.14), *0.042*	
	CSP NANP			304.28 (72.25;848.85), *0.002*							
	HBsAg	0.57 (0.33;0.82), < *0.001*	18.49 (4.39;34.49), *0.009*	19.24 (− 2.74;46.19), 0.09		55.36 (− 6.46;158.1), 0.09			− 4 (− 7.75;− 0.1), *0.045*	8.14 (1.42;15.3), *0.02*	− 5.15 (− 11.05;1.14), 0.11
IgM	CSP FL				− 76.82 (− 96.23;42.51), 0.11				− 14.68 (− 27.4;0.32), 0.055		
	CSP C-term		62.15 (6.71;146.4), *0.02*	95.03 (− 0.67;282.94), 0.052					− 18.52 (− 28.3;− 7.4), *0.002*		− 12.64 (− 26.4;3.73), 0.12
	CSP NANP	1.07 (0.05;2.09), *0.04*									
	HBsAg	2.17 (0.87;3.48), *0.001*	212.61 (97.4;395.11), < *0.001*	190.2 (38.4;508.6), *0.005*		120.31 (49.19;225), < *0.001*			− 15.86 (− 27.03;− 2.99), *0.02*		− 15.53 (− 30.01;1.95), 0.08

The coefficients indicate the percent change for a unit change in the predictor (95% confidence intervals), the *p* values indicated are for statistically significant covariates (in italics) and for those that improved the model. Malaria transmission season at month 3 sample collection (low vs high). Baseline antibodies to the same Ig/antigen. Baseline anti-*P. falciparum* exposure IgM levels (exposure PC1 index).

*Sex* male vs female, *Hb,g/dL* baseline hemoglobin, *WAZ* weight-for-age Z scores, *HAZ* height-for-age Z scores

*Continuous age at weeks. Site (Manhiça vs Kintampo)

**†**Malaria episode between month 0 and month 3 (yes vs no)

### RTS,S/AS01E-induced antibody correlates of malaria disease protection

Crude log_10_MFI levels of IgG, IgG1, and IgG3 to CSP constructs at M3 in RTS,S vaccinees were higher but not significantly in those who did not present with clinical malaria vs those who did over the 12-month follow-up period. In contrast, levels of IgG2 to all CSP constructs and of IgG4 to CSP FL were significantly lower in non-malaria controls than in malaria cases (Additional file 1: Figures S10–S13). A combined analysis of all IgG subclasses showed that the ratio of cytophilic (IgG1 + IgG3) to non-cytophilic (IgG2 + IgG4) antibodies to all CSP constructs was significantly higher among controls than cases in RTS,S vaccinees but not in comparator vaccinees (Fig. 5). Stratified by age, this difference occurred in children but not in infants (Additional file 1: Figure S14). Stratified by site, this was only seen for Kintampo but not for Manhiça (Additional file 1: Figures S15 and S16).

**Fig. 4:**
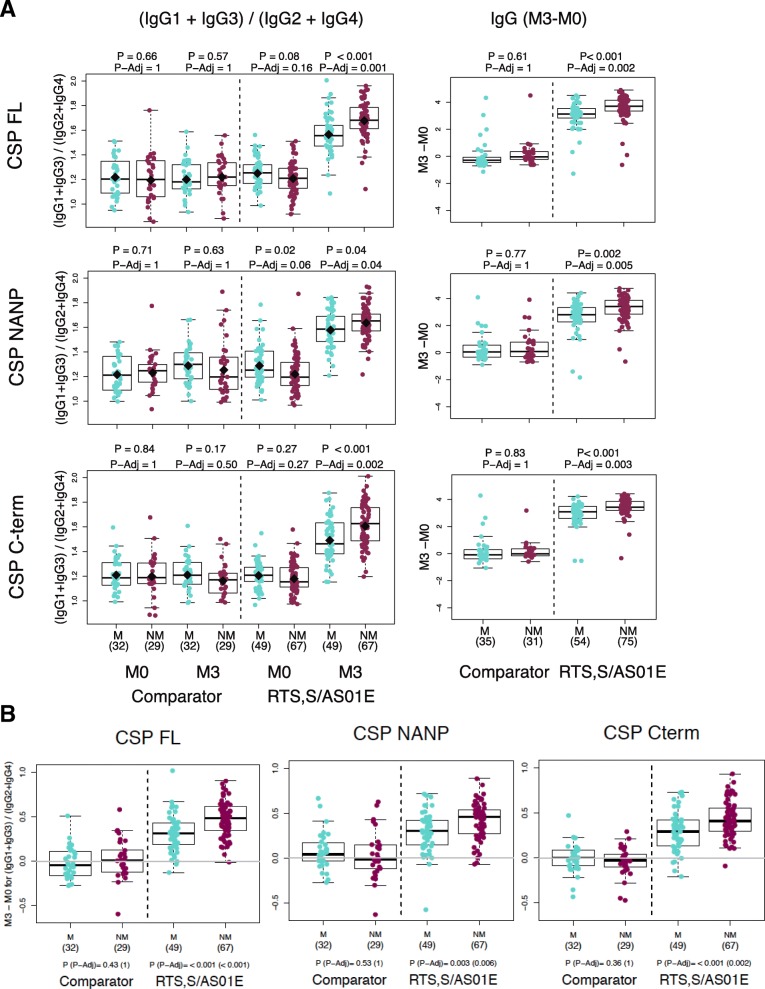
Association between CSP Ig responses after vaccination and RTS,S-induced protection. **a** Ratio of cytophilic IgG1 and IgG3 vs non-cytophilic IgG2 and IgG4 antibodies, and increment of IgG levels between month 0 and month 3 in protected (NM = no clinical malaria) and non-protected (M = clinical malaria). **b** Difference between month 3 and month 0 (M3–M0) cytophilic/non-cytophilic ratios. Groups were compared through *t* tests and *p* values adjusted by Holm

Baseline levels of CSP IgG were generally higher in subjects who developed clinical malaria than in those who did not (Additional file 1: Table S12). An analysis of the change in antibody responses between M0 and M3 in RTS,S vaccinees revealed significantly higher increase of Ig CSP levels in those who were protected than in those who subsequently suffered clinical malaria (Fig. 4). Stratified by age, this difference was significant in children but not in infants (Additional file 1: Figure S14). Stratified by site, this was more apparent for Kintampo than Manhiça (Additional file 1: Figure S15).

Regarding responses to the HBsAg component of the RTS,S vaccine, levels of IgG, IgG1, IgG3, and IgM at M3 in RTS,S vaccinees but not in comparators were lower in those who had clinical malaria over the 12-month follow-up period vs those who did not (Additional file 1: Figures S11–S13).

The covariates significantly associated with clinical malaria risk in univariate logistic regression models (including all vaccinees) were site, previous malaria episodes, WAZ, HAZ, baseline CSP IgG (and IgG1), *P. falciparum* maternal and exposure antibodies (Additional file 1: Tables S13–S14). When adjusted by site (Kintampo had higher odds of malaria than Manhiça), only age cohort and baseline *P. falciparum* maternal and exposure antibodies were statistically significant. Within RTS,S vaccinees, age cohort and baseline *P. falciparum* maternal antibodies remained significant. We next assessed if these covariates affected the associations between CSP and HBsAg Ig responses (M3 levels, M3-M0 changes, and M3 IgG subclass ratios) and clinical malaria in RTS,S vaccines. In multivariable regression models, the associations between antibody responses and clinical malaria remained statistically significant after correction for multiple comparisons for anti-C-term CSP IgG2 levels, M3–M0 changes in IgG and IgG1, and cytophilic to non-cytophilic IgG subclass ratio, as well as anti-HBsAg levels (IgG, IgG1, IgG3) and subclass ratio (Table 2). In these models, age and site significantly affected clinical malaria and, in some cases, also baseline antibodies. Sex was not significantly associated with clinical malaria, but there were some significant interactions with antibody responses to RTS,S vaccination; and antibodies appeared more strongly associated with malaria disease protection in males rather than females (data not shown). In addition, sex (*p* = 0.1) was retained in all models including C-term CSP IgG2 responses (Table 2).

**Table 2:** Association between RTS,S-induced antibody responses and protection against clinical malaria

		Univariate All subjects			Multivariable All subjects				Multivariable Subjects age < 10 months			
Antibody	Antigen	OR	95%CI	*p*	OR	95%CI	*p*	Covariates*	OR	95%CI	*P*	Covariates**
IgG M3	CSP FL	0.74	0.41; 1.23	0.25				*Age*, *site*, exposure				*Age*, *site*, exposure
	CSP C-term	0.58	0.28; 1.05	0.21	0.6	0.26; 1.18	0.17	Age, *site*, M0, exposure				*Age*, *site*, M0, exposure
	CSP NANP	0.67	0.37; 1.08	0.21				Age, *site*, M0				*Age*, *site*
	HBsAg	0.50	0.29; 0.82	*0.02*	0.38	0.2; 0.7	*0.002*	*Site*, exposure	0.42	0.18; 0.9	*0.03*	*Site*, *M0*, exposure, maternal
IgM M3	CSP FL	0.94	0.5; 1.75	1				*Age*, *site*, exposure				*Age*, *site*, M0
	CSP C-term	1.09	0.49; 2.44	1				*Age*, *site*, exposure				*Age*, *site*, M0
	CSP NANP	0.79	0.43; 1.44	1				*Age*, *site*, exposure				*Age*, s*ite*, M0
	HBsAg	0.46	0.25; 0.82	*0.03*				*Site*, *M0*, exposure				*Site*, *M0*, exposure
IgG1 M3	CSP FL	0.86	0.52; 1.36	0.54				*Age*, *site*, exposure				*Age*, *site*, exposure
	CSP C-term	0.60	0.34; 0.97	0.09	0.64	0.36; 1.06	0.1	Age, *site*, exposure				*Age*, *site*, M0, exposure
	CSP NANP	0.83	0.53; 1.25	0.48				*Age*, *site*, *M0*, exposure				*Age*, *site*, *M0,* exposure
	HBsAg	0.58	0.35; 0.92	*0.057*	0.49	0.27; 0.85	*0.01*	*Site*, exposure				*Age*, *site*, exposure
IgG2 M3	CSP FL	4.38	1.75; 12.29	*0.006*				Age, *site*				*Site*, *maternal*
	CSP C-term	4.08	2.07; 8.79	**<** *0.001*	2.62	1.18; 6.11	*0.02*	*Site*, sex	2.69	1.09; 7.32	*0.04*	*Maternal*, site, sex
	CSP NANP	2.63	1.2; 6.25	*0.049*				Age, *site*				*Maternal*, *site*
	HBsAg	0.76	0.37; 1.49	0.49				Age, *site*				*Maternal*, *site*
IgG3 M3	CSP FL	0.88	0.56; 1.35	0.54				*Age*, *site*, exposure				*Age*, *site*, exposure
	CSP C-term	0.75	0.44; 1.26	0.46				*Age*, *site*, exposure				*Age*, *site*, exposure
	CSP NANP	0.81	0.53; 1.21	0.46	0.63	0.38; 1.02	0.07	Age, *site*, exposure				*Age*, *site*, exposure
	HBsAg	0.19	0.06; 0.51	*0.004*	0.1	0.02; 0.34	**<** *0.001*	*Site*, exposure	0.26	0.06; 0.92	*0.05*	*Site*, maternal, exposure
IgG4 M3	CSP FL	2.15	1.31; 3.66	*0.009*				*Age*, *site*, exposure				*Age*, *site*, exposure
	CSP C-term	1.27	0.79; 2.04	0.46	1.63	0.92; 3	0.1	*Age*, *site*				*Age*, *site*, exposure
	CSP NANP	1.27	0.74; 2.24	0.48				*Age*, *site*, M0, exposure				*Age*, *site*, exposure
	HBsAg	0.11	0.01; 1.51	0.20				*Age*, *site*, exposure				*Age*, *site*, exposure
IgG M3–M0	CSP FL	0.47	0.27; 0.73	*0.001*				*Age*, *site*, exposure				*Age*, *site*, exposure
	CSP C-term	0.36	0.18; 0.65	*0.001*	0.51	0.26; 0.88	*0.03*	Age, *site*, exposure				*Age*, *site*, exposure
	CSP NANP	0.55	0.35; 0.8	*0.003*	0.72	0.46; 1.06	0.11	Age, *site*, exposure				*Age*, *site*, exposure
	HBsAg	0.73	0.49; 1.07	0.11				*Age*, *site*, exposure				*Age*, *site*, exposure
IgM M3–M0	CSP FL	0.72	0.43; 1.18	0.38				*Age*, *site*, exposure				*Age*, *site*, exposure
	CSP C-term	0.64	0.36; 1.11	0.35				*Age*, *site*, exposure				*Site*, *maternal*
	CSP NANP	0.62	0.38; 0.97	0.14				*Age*, *site*, exposure				*Age*, *site*, exposure
	HBsAg	0.79	0.42; 1.41	0.42				*Age*, *site*, exposure				*Age*, *site*, exposure
IgG1 M3–M0	CSP FL	0.52	0.33; 0.76	*0.007*				*Age*, *site*, exposure				*Age*, *site*, exposure
	CSP C-term	0.54	0.33; 0.81	*0.01*	0.63	0.39; 0.96	*0.04*	Age, *site*, exposure	0.66	0.4; 1.02	0.07	*Age*, *site*, exposure
	CSP NANP	0.63	0.44; 0.86	*0.01*	0.75	0.52; 1.03	0.08	Age, *site*, exposure	0.78	0.54; 1.08	0.14	*Age*, *site*, exposure
	HBsAg	0.73	0.51; 1.05	0.19				*Age*, *site*, exposure				*Age*, *site*, exposure
IgG2 M3–M0	CSP FL	0.72	0.41; 1.21	0.34				Age, *site*				*Site*, *maternal*
	CSP C-term	2.30	1.29; 4.33	*0.02*	1.86	0.95; 3.81	0.08	*Site*, sex	2.46	1.1; 6.13	*0.04*	*Site*, *maternal*, sex
	CSP NANP	0.86	0.52; 1.43	0.60				Age, *site*				*Site*, *maternal*
	HBsAg	1.32	0.65; 2.73	0.56				Age, *site*				*Site*, *maternal*
IgG3 M3–M0	CSP FL	0.65	0.45; 0.91	*0.03*	0.77	0.52; 1.11	0.16	Age, *site*, exposure				*Age*, *site*, exposure
	CSP C-term	0.79	0.53; 1.17	0.35				*Age*, *site*, exposure				*Age*, *site*, exposure
	CSP NANP	0.72	0.53; 0.97	0.07	0.73	0.52; 1.02	0.07	Age, *site*, exposure				*Age*, *site*, exposure
	HBsAg	0.76	0.48; 1.16	0.34				*Age*, *site*, exposure				*Age*, *site*, exposure
IgG4 M3–M0	CSP FL	1.67	1.08; 2.66	*0.053*				*Age*, *site*, exposure				*Age*, *site*, exposure
	CSP C-term	1.15	0.78; 1.71	0.56	1.44	0.9; 2.37	0.14	Age, *site*				*Age*, *site*, exposure
	CSP NANP	1.19	0.75; 1.9	0.56				*Age*, *site*, exposure				*Age*, *site*, exposure
	HBsAg	0.78	0.08; 7.09	0.82				*Age*, *site*, exposure	18.1	0.57;967	0.11	*Age*, *site*, exposure
IgG1 + IgG3/IgG2 + IgG4 M3	CSP FL	0.01	0; 0.12	**<** *0.001*	0.11	0.01; 1.9	0.13	Age, *site*				*Site*, *maternal*
	CSP C-term	0.03	0; 0.22	*0.002*	0.03	0; 0.35	*0.007*	*Site*, sex	0.04	0; 0.79	*0.04*	*Site*, *maternal*, sex
	CSP NANP	0.06	0; 0.82	*0.07*	0.06	0; 1.26	0.07	Age, *site*, exposure				*Site*, *maternal*
	HBsAg	0.07	0; 1.14	*0.07*	*0*	0; 0.15	*0.004*	*Site*, exposure				*Site*, *maternal*

Logistic regression models of the association between (i) levels of antibodies (log_10_MFI) at month (M) 3, (ii) increments from M0 to M3 antibodies (iii), ratio of cytophilic to non-cytophilic antibodies at M3, and clinical malaria (yes/no), in RTS,S/AS01E vaccinees, expressed as odds ratio (OR), 95% confidence interval (CI), and *p* values (corrected for multiple comparisons, see the “Methods” section). The associations between predictors and outcome shown here were not found in comparator vaccines at month 3 (data not shown). Multivariable analysis models were done with the minimum Akaike information criterion (AIC); statistically significant variables are marked in italics. A combination of backward and forward stepwise algorithms was used to obtain the model with the minimum AIC.

*Variables that were statistically significant (italics) or improved the multivariable model. Exposure = malaria exposure antibody index, age in weeks; M0 = baseline antibody levels to the respective antigen/Ig

**Analyses included also antimalarial maternal antibody index (maternal)

**Fig. 5:**
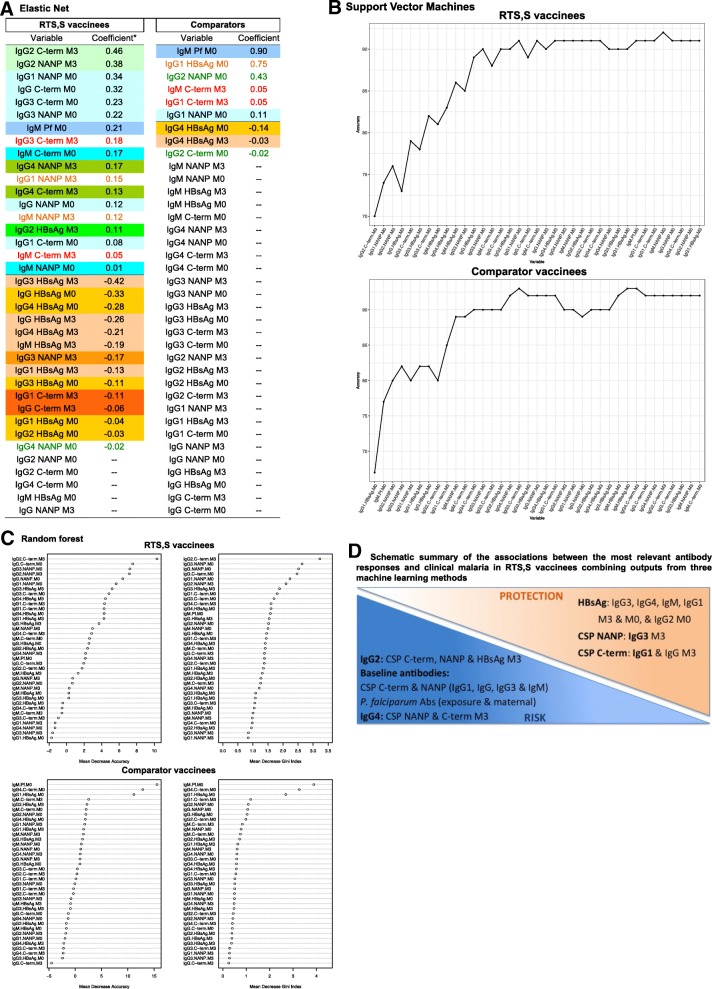
Multi-marker correlates analyses by machine learning techniques. Results from three complementary methods are shown stratified by vaccination group. **a** Elastic Net. **b** Support vector machines. **c** Random forest. **d** Schematic summary of the associations between the most relevant antibody responses and clinical malaria in RTS,S vaccinees combining outputs from three machine learning methods

Machine learning multi-marker analyses (Fig. 5) revealed a signature of protection against clinical malaria in RTS,S vaccinees composed of antibodies to HBsAg (IgG3, IgG4, IgM, and IgG1 at M3 and M0, and IgG2 at M0), to CSP NANP (IgG3 at M3), and C-term (IgG1 and IgG at M3). In analysis stratified by age and site, IgG3 NANP was more prominent in infants in Manhiça, and IgG1 C-term in children in Kintampo (Additional file 1: Table S14). Moreover, a signature of malaria disease risk was also identified composed of IgG2 responses to CSP C-term, NANP, and HBsAg at M3; baseline responses to CSP C-term, NANP (IgG1, IgG, IgG3, and IgM), and *P. falciparum* antigens (exposure and maternal indices); and IgG4 responses to CSP NANP and C-term at M3 (Fig. 5). In addition, IgG3 and IgM to CSP C-term at M3 (in detriment of IgG1), and IgG1 and IgM to CSP NANP at M3 (in detriment of IgG3), were also associated with increased risk. In stratified analysis, M0 IgG2 to CSP NANP and M0 IgG4 to CSP C-term were also associated with risk in infants. These protective signatures were not found in comparator vaccinees, in whom malaria exposure index was the strongest risk variable.

## Discussion

Our study confirms prior knowledge on RTS,S/AS01E immunogenicity and goes a significant step beyond in understanding its determinants and mode of action. The data corroborate that vaccination induces a substantial increase in anti-CSP and HBsAg IgG responses, higher in children than in infants, which contributes to protection against clinical malaria [18]. In addition, a set of novel findings expand the breadth of knowledge about RTS,S immunogenicity and potential correlates of protection.

First, we showed that antibodies to other CSP epitopes in its C-terminal region are also elicited by RTS,S/AS01E in African children (although seemingly at lower levels compared to NANP repeat) and are the only ones associated with clinical malaria protection in multivariable analysis in our study population. Inclusion of a CSP FL construct allowed detecting responses of lower magnitude possibly due to the additive response to multiple B cell epitopes.

Second, we characterized Ig responses not previously assessed, including IgM and IgG subclasses for all antigens. We found that IgG1 predominates upon RTS,S vaccination, followed by IgG3, with lower production of IgG2 and IgG4. Furthermore, our data support that the balance of anti-CSP IgG subclasses more than the total IgG levels is important for protection against clinical malaria, as a higher ratio of cytophilic IgG1 and IgG3 to non-cytophilic IgG2 and IgG4 antibodies was associated with antimalarial immunity. Thus, children who predominantly produced anti-CSP (particularly C-term) IgG2 and IgG4 in detriment of IgG1 and IgG3 as a response to vaccination were at higher risk of suffering malaria disease. This is consistent with the notion that acquired immunity is attributed to cytophilic rather than non-cytophilic IgGs [37] due to their functional capacity to fix complement and opsonize parasites for Fc binding and phagocytosis [38, 39]; these mechanisms could also be acting in CSP-mediated sporozoite immunity. Antibodies to C-term CSP seem not important in blocking hepatocyte invasion by sporozoites but could mediate these other protective mechanisms. Recent studies have shown that acquired human antibodies, and antibodies to CSP, can fix and activate complement on the sporozoite surface, leading to inhibition of motility [40]. This potential antibody-mediated mechanism of action of RTS,S needs to be confirmed in future functional studies. Our findings are, however, not in line with observations by Chaudhury et al. [41], where IgG4 was associated with protection against sporozoite challenge in RTS,S-vaccinated naïve adults and IgG4 inhibited phagocytosis mediated by IgG1 and IgG3 when using the THP1 pro-monocytic cell line. That study also found that phagocytic activity using THP1 cells was not associated with vaccine efficacy. It is possible that the mechanisms of immunity differ between vaccinated malaria-naive adults and children resident in a malaria-endemic region. Furthermore, using THP-1 cells may not represent all Fc-receptor interactions that occur with phagocytes in vivo due to differences in Fc-receptor expression and function between THP1 cells, monocytes, neutrophils, and other cells. Recent studies have also highlighted differences in opsonic phagocytosis activity when using purified monocytes versus whole-blood assays [42].

Third, we characterized the effect of baseline status, including variables related to malaria exposure, on RTS,S immunogenicity and efficacy. At the individual level, the presence of antibodies at the time of vaccination due to maternal transfer and/or to past/present infections or to other vaccinations [43, 44] likely varies in infants and children and may differentially affect RTS,S outcomes. As expected, Kintampo had higher M0 levels of CSP antibodies than Manhiça. The site did not seem to have a major role in CSP antibody levels at M3, but because of the different baselines, it had a significant effect on change from M0 to M3 CSP responses. Thus, individuals exposed to higher MTI had higher M0 Ig levels and lower change in antibodies from M0 to M3. This was manifested differently depending on age group: (i) higher M0 IgG levels in infants than children presumably represented passively transferred maternal antibodies that, in the absence of vaccination (comparators), decayed from M0 to M3, and these were generally higher in Kintampo than Manhiça due to higher malaria exposure in the mothers; (ii) higher M0 IgG and IgM levels in children represented those acquired upon infection and were also higher in Kintampo than Manhiça. Season did not impact the outcomes (consistent with a recent report [45]) as the majority of volunteers were vaccinated within restricted time periods.

A prior analysis of the effect of baseline anti-NANP CSP IgGs within the phase 3 trial [18] reported that pre-vaccination titers were associated with lower RTS,S immunogenicity in infants and higher immunogenicity in children. We also found a significant negative association between anti-NANP CSP pre-vaccination levels and post-vaccination IgG and IgG1 responses, particularly in younger children. At baseline, anti-CSP IgGs in infants < 10 months correlated with IgG to *P. falciparum* antigens used as markers of maternal antibodies, and anti-CSP IgGs in children correlated with IgM to *P. falciparum* antigens that are markers of current/recent exposure. These maternal and exposure indices, obtained using an unprecedented breadth of pre-erythrocytic and erythrocytic stage proteins, were significantly associated to some CSP and HBsAg Ig responses at M3. Overall, the data show that high concentrations of maternal CSP IgG at baseline could interfere with RTS,S immunization by binding the vaccine proteins and impeding antigen presentation and subsequent response, resulting in lower immunogenicity in newborns.

More importantly, higher malaria exposure prior to M0 positively predicted the occurrence of malaria cases over the 12-month follow-up period after M3, while lower levels of antibodies to CSP or *P. falciparum* antigens at M0 predicted lower risk of clinical malaria during the post-vaccination follow-up; this also applied for comparator vaccinees at M3. Here, naturally acquired CSP antibody levels also indicated malaria exposure. In this line, it is well established that the strongest risk factor for future malaria disease is having had malaria episodes in the past [37]. In fact, malaria events prior to M3 rendered participants more susceptible to future malaria episodes, but this was largely explained by site, with higher baseline MTI and malaria incidence in Kintampo than Manhiça. As RTS,S vaccination induced a potent increase in anti-CSP antibodies at M3, individuals protected against clinical malaria had a significantly higher increase in M0 to M3 Ig levels than the non-protected, more remarkably for C-term Ig. The associations were significant mostly for Kintampo (where the effect of malaria exposure was heavier) and children (in whom there were little maternal antibodies and RTS,S elicited higher M3 Ig levels). Thus, it appears that RTS,S exerts a larger benefit (vaccine efficacy) when vaccinees have had less malaria exposure before M0, thus that they are able to mount a higher (on average) CSP IgG response at M3 upon vaccination, and this contributes more efficiently to controlling *P. falciparum* infection. This is in line with the results from the main MAL055 trial with regard to the significant interaction between vaccine efficacy and site in children (not infants) whereby lower efficacy was generally estimated in sites of higher MTI [14].

We and others have shown that malaria exposure alters the phenotype and functional characteristics of memory B lymphocytes [46, 47] and other cells, e.g., T helper [48], that are responsible for antibody production. Individuals under higher MTI may have an immune system that is suppressed, de-regulated, or primed by natural exposure to produce a different immune response that upon RTS,S vaccination could deviate CSP response to predominantly non-cytophilic antibodies and/or overall lower IgG levels. Elicitation of IgE rather than IgG CSP responses [49] and of T_H_2 rather than T_H_1 [23] may also be associated with lower RTS,S efficacy. Induction of greater IgG1 and IgG3 relative to lower IgG2 and IgG4 in individuals under lower MTI would lead to a better quality and more balanced immune response associated with functional CSP antibody-mediated vaccine protection. Our data strongly support this assertion. Levels of IgG2 to all CSP constructs and IgG4 to CSP FL (but not of IgG1 or IgG3) induced by RTS,S vaccination were significantly higher with heavier MTI. Other baseline variables not previously assessed in RTS,S studies could influence the immune balance, and particularly, the role of sex deserves more investigation because of the increased overall mortality in girls than boys found in a secondary analysis [50]. Baseline Hb, WAZ, and HAZ were associated with antibody levels at M3, suggesting that the health status of the child might also affect the initial response to the vaccine, but this was not significant after adjustment. Due to sample sizes, these observations should be interpreted with caution but merit further assessment. Thus, determinants of IgG2, IgG4, or IgE CSP responses and exhausted T_H_ and B cells associated with malaria infections need to be disentangled as they appear relevant for vaccine success.

Remarkably, higher HBsAg antibody levels were associated with less malaria disease risk in RTS,S vaccinees, and this was consistent across Ig isotypes, ages, and sites and confirmed in machine learning analysis. This unforeseen result could be an indirect association related to a better general immune status in the volunteers protected against clinical malaria, who may respond more potently to non-malarial antigen epitopes upon vaccination, or a surrogate of other protective mechanisms. Alternatively, there may be a direct immunological mediation whereby HBsAg-specific T cells might provide help to B cells to produce CSP antibodies by virtue of being presented together (hapten-carrier hypothesis). The complex relationship between malaria exposure, HBsAg antibodies, and clinical malaria risk will be the subject of future investigations.

Our study design had some limitations that can be mitigated in follow-up studies. In Kintampo, all children who fulfilled the inclusion criteria, most of whom had malaria, were analyzed. Whereas in Manhiça, a case-control design was needed because there were fewer cases; a cohort design would have required to increase the number of samples substantially to have power to detect association with protection. In Manhiça, there was also an age imbalance, as most cases were in infants. Despite this and having many markers and multiple comparisons, the results were coherent and biologically plausible. Thus, the impacts of CSP IgG subclasses, baseline malaria exposure, and of HBsAg Ig levels, on clinical malaria risk, were consistent.

## Conclusions

Our characterization of an expanded breadth of antigen epitopes and Ig isotypes/subclasses induced by RTS,S, including non-CSP *P. falciparum* antigens as markers of maternal antibodies and malaria exposure, led to a better understanding of baseline determinants of vaccine take and protection against clinical malaria. We identified new potential correlates of malaria disease risk and protection, including IgG subclasses, baseline antibody levels, and HBsAg antibodies. These data shed new light into the mode of action of RTS,S, further evidencing that it is more complex and multifactorial than previously thought. Future studies should assess the function of the antibodies, their correlation with cellular immune responses associated with clinical malaria protection and risk, and the kinetics of peak and post-booster CSP and HBsAg responses over time. Elucidating mechanisms of RTS,S immunity and correlates will be translated into a more rational development, testing, and deployment of next-generation vaccines. Protective responses identified could be favored with appropriate adjuvants, delivery systems, and/or vaccination schedules, including combination with antimalarial drugs.

## Additional file

Additional file 1: Supplementary methods (DOC 11140 kb)
