# Supplementary material for: Baseline exposure, antibody subclass, and hepatitis B response differentially affect malaria protective immunity following RTS,S/AS01E vaccination in African children
Source: BMC Med. 2018 Oct 31;16:197. doi: 10.1186/s12916-018-1186-4 (PMC6208122; doi:10.1186/s12916-018-1186-4)
Supplement: Supplementary file 1 — Supplementary methods (DOC 11140 kb) [file 12916_2018_1186_MOESM1_ESM.doc]

**Additional File 1**

**Supplementary Methods**

**Antibody measurements**

Quantitative suspension array technology (qSAT) assays included bovine serum albumin (BSA) coupled beads for background determination. Glutathione S*-*transferase (GST) coupled beads were also added to the panel as a control for background signal coming from nonspecific binding in the cases where *Plasmodium falciparum* proteins were fused to GST. Proteins were covalently coupled directly to MagPlex beads and blocked with BSA.

Antigen-coupled beads were added to a 96-well μClear® flat bottom plate (Greiner Bio-One) in multiplex (1,000 microspheres/analyte/well) resuspended in 50µL of PBS, 1% BSA, 0.05% Azide pH 7.4 (PBS-BN). Fifty µL of sample, negative or positive control were added to multiplex wells and incubated overnight at 4ºC in a shaker protected from light. Plates were washed three times with 200µL/well of wash buffer (PBS-Tween 20 0.05%) using a manual magnetic washer. Then, 100µL of biotinylated secondary antibody were added diluted in PBS-BN: anti-human IgG (Sigma), anti-human IgM (Sigma), anti-human IgG1 (Abcam) and anti-human IgG3 (Sigma). For IgG2 and IgG4, we added mouse anti-human IgG4 (Thermo) and mouse anti-human IgG2 (Thermo Fisher), followed by biotinylated goat anti-mouse IgG (Sigma) in PBS-BN. All antibody incubations were performed for 60 min, at room temperature, with agitation and protected from light. Next, streptavidin-R-phycoerythrin (Sigma) in PBS-BN was added to all wells and incubated 30 min, at room temperature, with agitation and protected from light. Plates were washed as before and resuspended in 100 μL/well of PBS-BN. Plates were stored at 4°C overnight protected from light and read the next day using the Luminex xMAP® 100/200 analyser; at least 50 microspheres per analyte were acquired per sample.

Test samples were assayed at 4 dilutions for IgG (500, 5000, 50,000 and 500,000), IgG1, IgG3 (100, 1000, 10,000 and 100,000) and IgM (100, 1000, 10,000 and 50,000), and 2 dilutions for IgG2 and IgG4 (50 and 500) to ensure that at least one dilution lie in the linear range of the respective standard curve, i.e. close to the highest slope between two dilution points. For IgG assays, 18 to 22 serial dilutions 1:2 of a positive control were used to perform antigen-isotype/subclass specific standard curves . The positive control consisted of a WHO Reference Reagent for anti-malaria *P. falciparum* human serum (NIBSC code: 10/198) at 1:50 plus a pool of plasmas from RTS,S/AS02 vaccinated children with high IgG titers against CSP at 1:100. For the IgM assay, 18 serial dilutions 1:2 of a pool of samples from ISGlobal repository with high IgM levels against *P. falciparum* antigens were used . A total of 69 different negative control samples from malaria-naïve adult donors were assayed along the study to calculate the cutoffs of seropositivity (mean + 3 standard deviations [SD]). Blanks were added to each plate in triplicates for quality control purposes. Sample distribution across plates was designed to ensure a balanced distribution of vaccination groups, age cohorts and time-points. Data were captured using xPonent software.

**Data pre-processing**

The assay quality control for each antigen and plate was based on the estimation of the % coefficient of variation (CV) of the 3 blank controls (Fig. S1A). The antigen-isotype/subclass specific lower limits of quantification (LLOQ) and lower limits of detection (LLOD) were calculated using the blanks mean + 10 SD and blanks mean + 3 SD, respectively . The standard curve for each antigen-isotype/subclass-plate was estimated using the *drLumi* R package flow , fitted in a 5-parameter logistic (5-PL) regression model, and data points logarithmically transformed. If the model did not converge, 4-PL or exponential regressions were fitted. The standard curves were visually inspected, and the percentage of plates within an analyte-isotype/subclass with CV Emax SE/Beta <15%, CV Emin SE/Beta <15%, R^2^ >0.95 and Model fit p value >0.05, were calculated.

To select the sample dilution in the linear part of the sigmoidal curve (antigen, isotype/subclass and plate specific), an algorithm that detects the two points with the highest slope between them was used. The slope was computed as: m = (log_10_ MFI_i_ – log_10_ MFI_i+1_) / (dilution_factor_i_ – dilution_factor_i+1_). The mean log_10_ MFI value of the two points was computed, and the nearest log_10_MFI of the test sample and the corresponding dilution was selected. For some antigens, IgG2 and IgG4 standard curves did not converge, as there is no appropriate positive standard control for these Ig subclasses. In these cases, we assigned the first sample dilution. The MFI measurement of the selected dilution was corrected multiplying by its corresponding dilution factor and transformed to log_10_ scale to stabilize the variance.

Blank and GST background signals were not subtracted. GST subtraction distorted and increased the variability of the data due to the lack of correlation between GST-fusion proteins and GST alone (Fig. S1B).

**Basic descriptive statistical analyses**

Demographic and other subjects’ characteristics were compared across vaccination groups through t-tests, when reporting means, Wilcoxon-rank sum tests, when reporting medians, and Pearson Chi^2^ or Fisher exact tests, when reporting proportions. Pairwise correlations within Ig isotypes/subclasses across antigens, and within antigens across Ig isotypes/subclasses, were evaluated through scatterplots with loess and regression lines and Spearman coefficients (rho) with 95% CI. Descriptive comparisons of Ig levels across antigens and isotypes/subclasses were done through boxplots with geometric means, medians and interquartile ranges (IQR), by t-tests, and p-values adjusted by the Holm approach for IgG and IgM, or the Benjamini-Hochberg approach for IgG_1-4_, considering all antigens together. Proportion of positive antibody responders was compared through cross tabulations and Pearson Chi^2^ tests. Analyses included either all subjects or separately by visit and by vaccination, and in some cases stratifying by site, by age, and by age group within a site.

**Mixed models equations**

The fixed effects of the multivariable mixed effects models used to assess RTS,S/AS01E immunogenicity were given as: log_10_MFI = β__ + β_1_ · RTS,S + β_2_ · M3 + β_3_ · RTS,S · M3 + ε where RTS,S was a binary indicator being 1 when a subject was vaccinated with RTS,S and 0 otherwise; M3 was 1 when Ig levels were measured at M3 and 0 in case it was M0. Thus, two questions could be answered by combining fixed effects coefficients: difference between RTS,S and comparator at M3 (β__ + β_3_), and change from M0 to M3 attributable to RTS,S (β_3_).

The mixed effects models estimated across age cohorts (children and infants) incorporated new terms (β_4_ · covariate + β_5_ · RTS,S · covariate + β_6_ · M3 · covariate + β_7_ · RTS,S · M3 · covariate) to assess the main effect of age (covariate), as well as the interaction between the covariate, vaccination group and timepoint. Likelihood ratio test p-values were obtained for the main effect of age, as well as the interaction between the covariate, vaccine and timepoint. Through linear combination of coefficients, the effect of vaccination on the ratio between the RTS,S and comparator-vaccinees antibody levels at M3 (i.e. the immunogenicity specifically induced by RTS,S as opposed to natural exposure and not affected by regression to the mean) (β_1_ + β_3_ + β_5_ + β_7_), and change from M0 to M3 within RTS,S (β_2_ + β_3_ + β_6_ + β__), could be estimated in each age group.

**Results**

**Baseline characteristics**

RTS,S and comparator vaccinees were similar at baseline (Table S1) and most of them (93%) completed the 12-month post-vaccination follow-up. The median time to drop out of the study in those not completing the 12-month follow-up was 113 days, ranging from 21 to 276, and was mostly early terminations due to loss to follow-up (7 subjects) or migration (4 subjects). A total of 89 clinical malaria events were recorded during the follow-up time (60 in Kintampo and 29 in Manhiça); 39% of those in the children age category and 61% in the infant age category. Parasite densities in subjects who had an event were comparable between RTS,S and comparator vaccines (Table S1).

**Magnitudes of isotype and subclass antibody responses to RTS,S vaccine antigens**

Antibody levels ranged between 3.57 and 10.18 log_10_ median fluorescence intensity (MFI) (Fig. S2). The highest IgG responses were for CSP FL followed by NANP and C-term. With regards to IgG subclasses, the highest ranges of responses were observed for IgG1 and IgG3, and the lowest for IgG2 and IgG4; HBsAg IgG4 responses were negligible (Fig. S2).

Within each Ig isotype and subclass, all pairwise Spearman correlations were strong amongst CSP antigens (range rho=0.74-0.88), except within IgG4 where correlations were moderate (rho=0.40-0.55) (Table S2, Fig. S3). Correlations between CSP and HBsAg antibodies were moderate within IgG and IgG1 (rho=0.65-0.70), weak within IgM, IgG3 and IgG4 (rho=0.25-0.37), and very weak within IgG2 (rho=0.08-0.09).

Within each antigen, correlations between IgG and IgG1 were very strong for all antigens (rho >0.90) (Table S2). Generally, correlations across isotypes/subclasses within antigens ranged from moderate to strong. The lowest correlations were for IgG4 (rho=0.30-0.64) and IgM (rho=0.30-0.66) with all other isotypes/subclasses, particularly within CSP C-term (rho=0.41-0.55). Correlations across isotypes/subclasses of anti-HBsAg antibodies ranged from very weak to weak/moderate (rho=0.07-0.46), except for the association of IgG and IgG1 (rho=0.89).

**Effect of other variables on immunogenicity**

Sex was not significantly associated with CSP IgG nor IgM responses. Weight-for-age Z score (WAZ), height-for-age Z score (HAZ) or hemoglobin (Hb) concentration at baseline were not associated either for IgG responses to RTS,S, but there were statistically significant negative associations between WAZ and CSP IgMs, for C-term (at M0: Coefficient [confidence interval, CI] -20.05 [-30.52;-8.01], p-adj 0.02) and NANP (in comparators at M3: -33.54 [-48.71;-13.87], p-adj 0.03). Similarly, there were significant negative associations between baseline Hb concentrations and IgM levels to CSP C-term (at M0: -20.38 [-28.51;-11.34], p-adj <0.001; and M3 RTS,S vaccines: -16.69 [-26.57;-5.47], p-adj 0.049) and CSP FL (at M0: -18.62 [-27.32;-8.87], p-adj 0.005), predominantly in children.

In multivariable analyses, sex was not statistically significant, but some trend differences were noted for IgG2 and IgG4 (Table S11). Also in multivariable analyses, new associations emerged for Hb, WAZ and HAZ on IgG (not only IgM) responses, particularly lower M0 Hb concentrations correlated with higher M3 HBsAg IgG, IgG1, IgG4 and IgM levels (Table S11).

**Supplementary Figures and Tables**

**A**

**B**

**Figure S1. Quality control/quality assurance and pre-processing of antibody data.** Examples of **A)** percentage of samples below lower limit of quantification (LLOQ) and lower limit of detection (LLOD) for IgG responses to all antigens tested. **B)** IgG levels (median fluorescent intensity [MFI]) to CSP full length, CSP C-term and CSP-NANP vs IgG levels to GST RTS,S/AS01E and comparator vaccines data are combined.

**A**

| 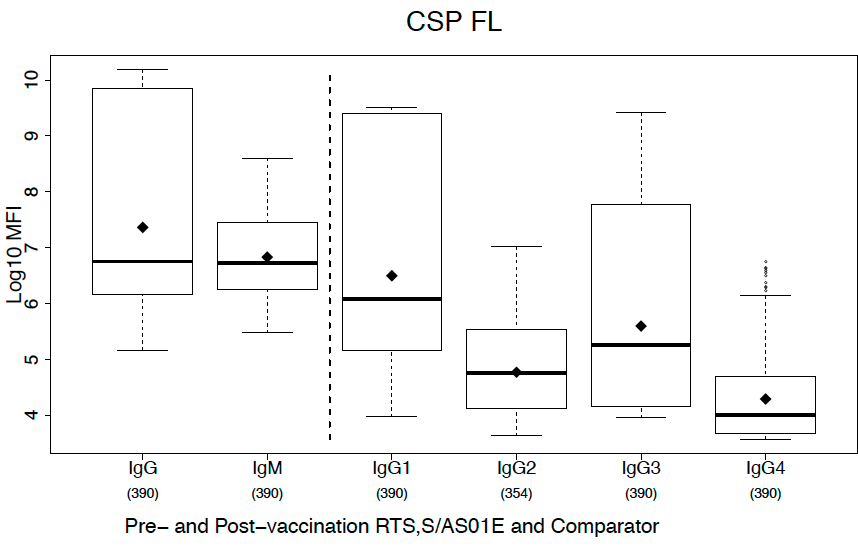 | 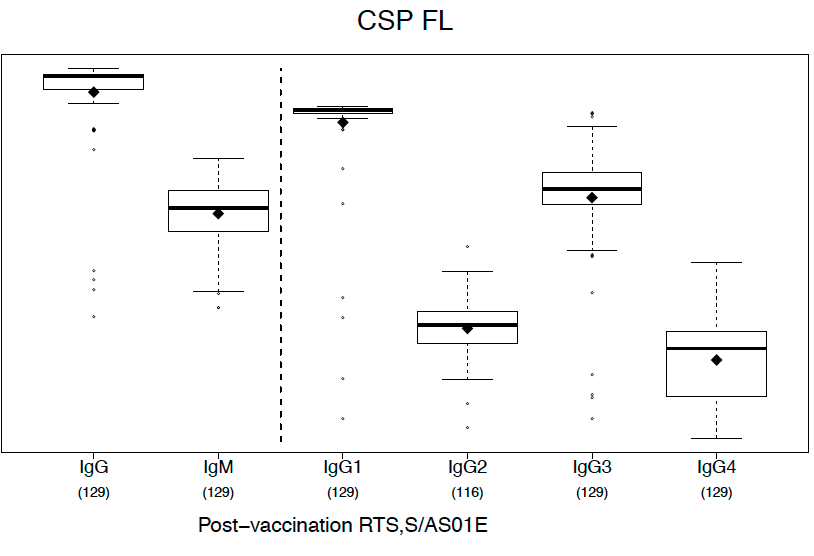 |
| --- | --- |
| 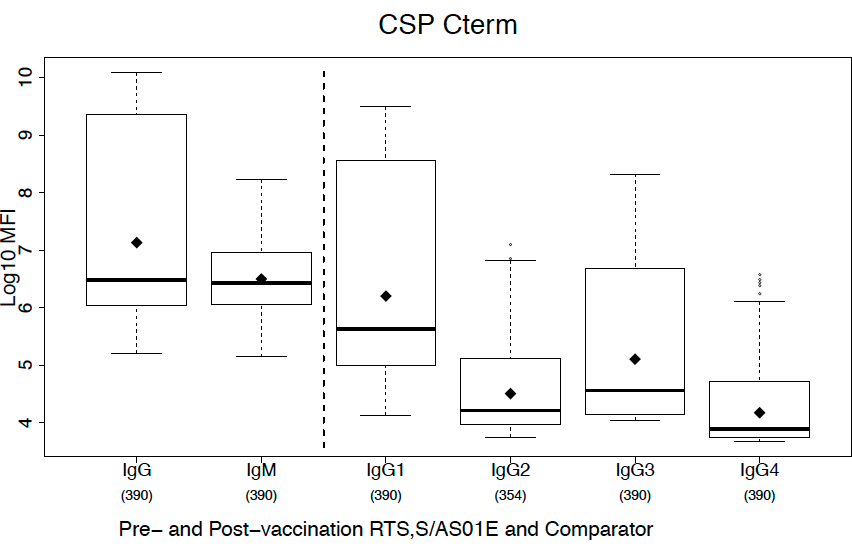 | 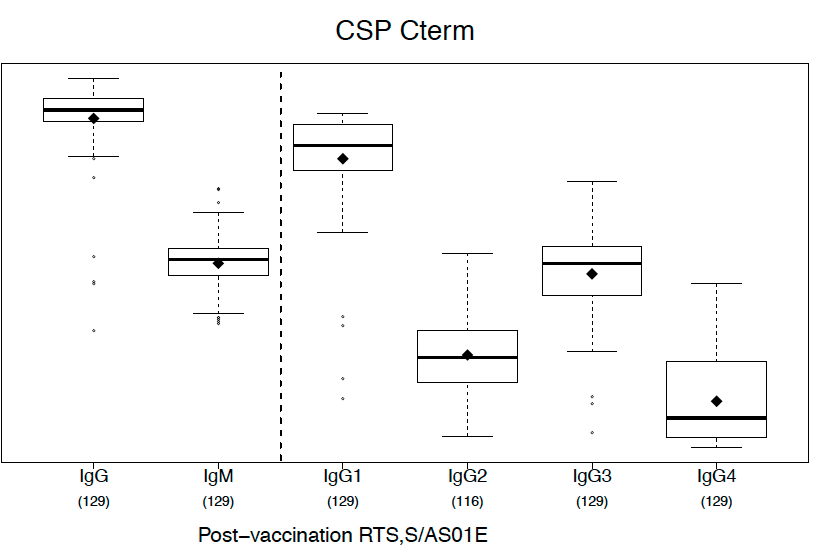 |

B

| 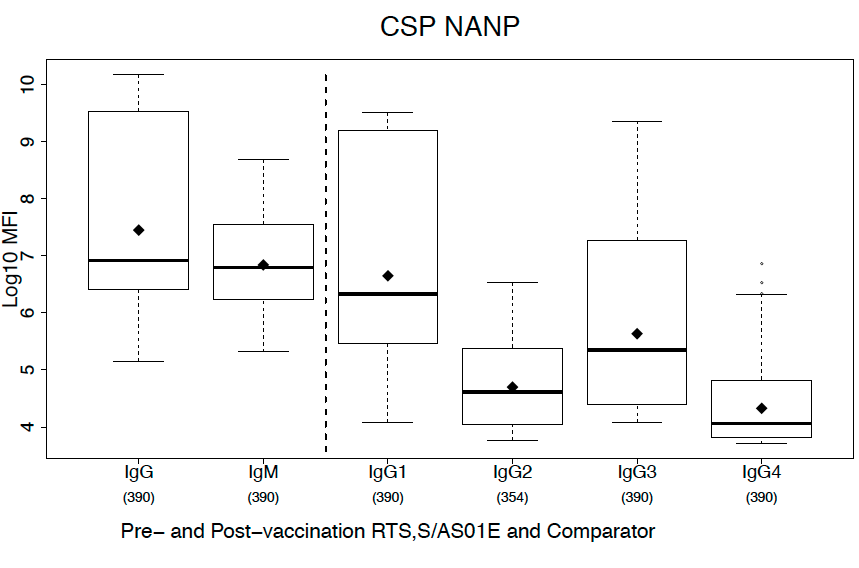 | 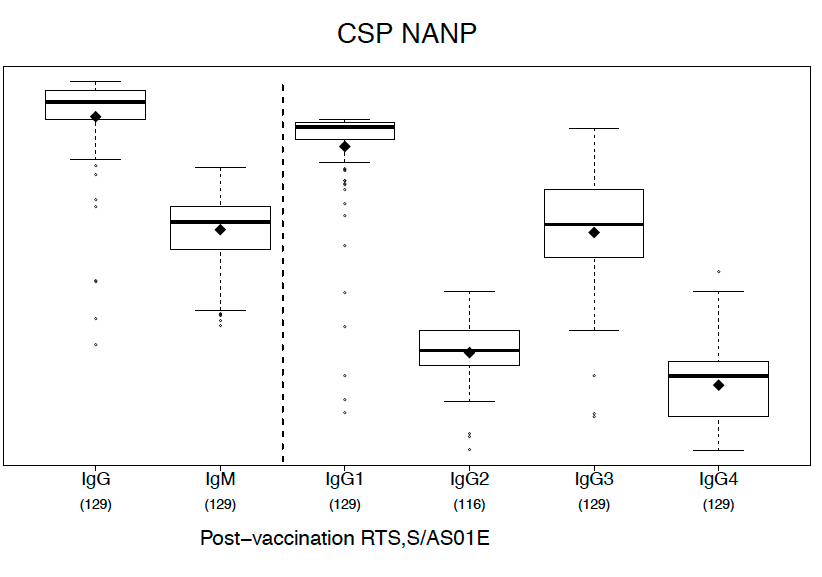 |
| --- | --- |
| 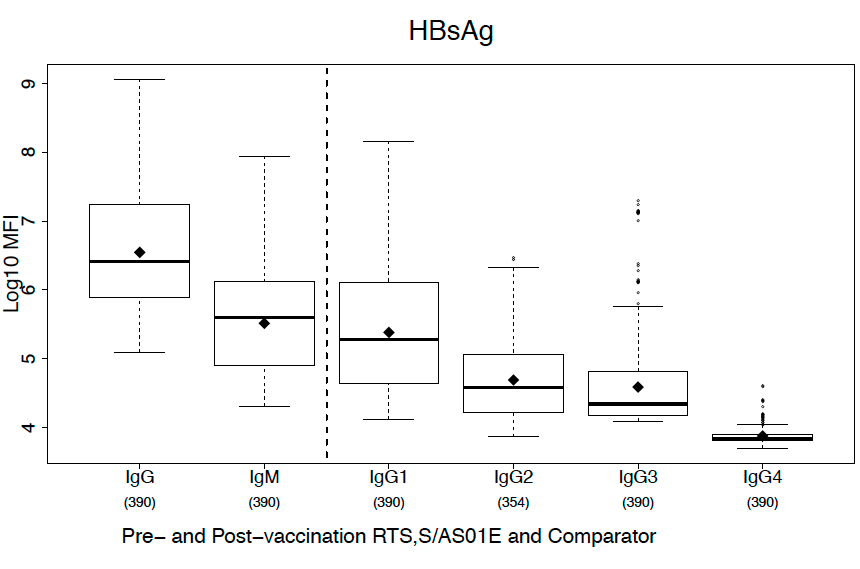 | 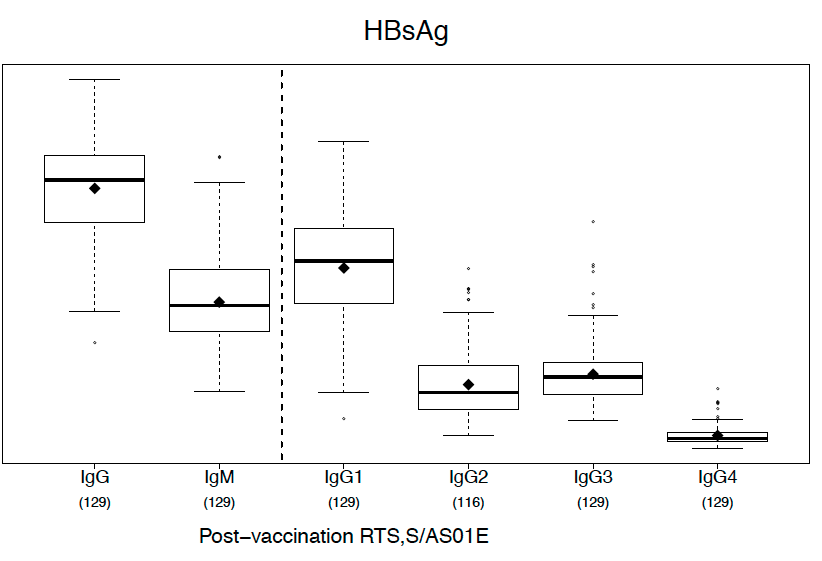 |

**Figure S2. Comparison of isotype and subclass antibody responses to CSP constructs and HBsAg. (A)** At both time points (pre- and post-vaccination) for RTS,S/AS01 and comparator vaccinees together, and **(B)** post-vaccination for RTS,S/AS01 vaccinees only. The solid line in the boxplot represents the median and the diamond represents the geometric mean. The highest IgG responses were for CSP FL (median 10.05; interquartile range [IQR] = 9.83-10.09) observed post-vaccination in RTS,S/AS01E vaccinees, followed by CSP NANP (median 9.82; IQR = 9.52-10.02), and CSP C-term (median 9.54; IQR = 9.35-9.75). CSP IgM responses ranged from 5.15 to 8.68; the highest responses were also recorded for post-RTS,S/AS01E vaccination against CSP FL (median 7.71; IQR = 7.30-8.02) followed by CSP NANP (median 7.74; IQR = 7.26-8.0), and CSP C-term (median 6.70; IQR = 6.73-7.19).

**A) B)**

**
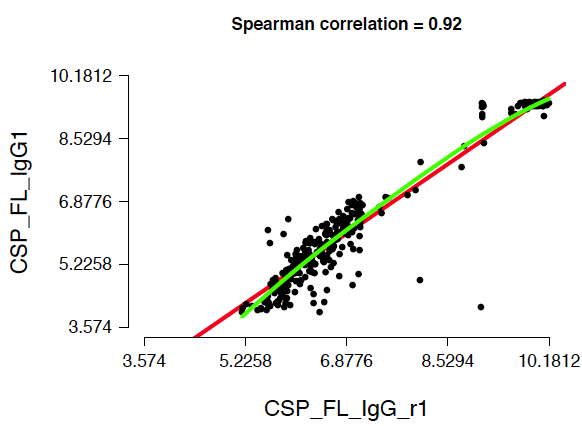
** **
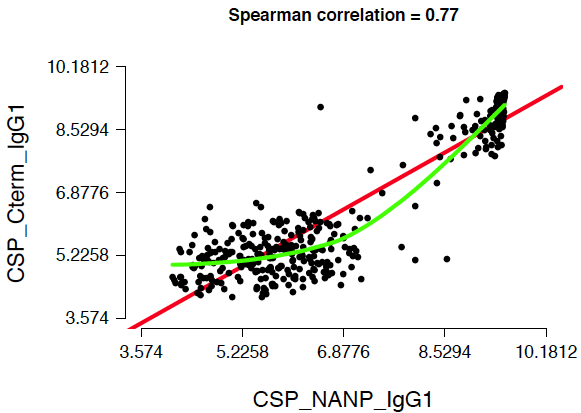
**

**C) D)**

**
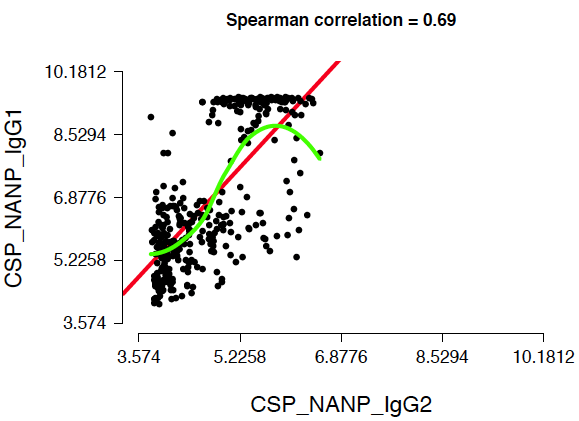
** **
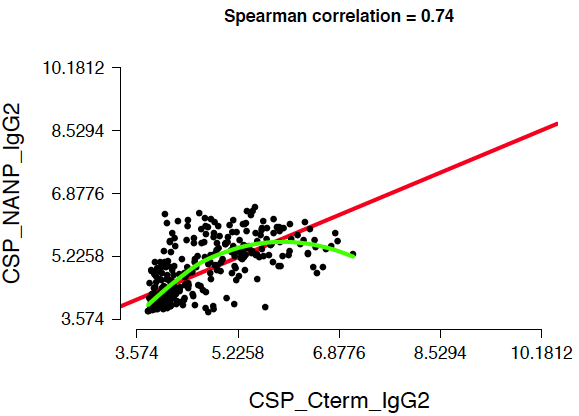
**

**E) F)**

**
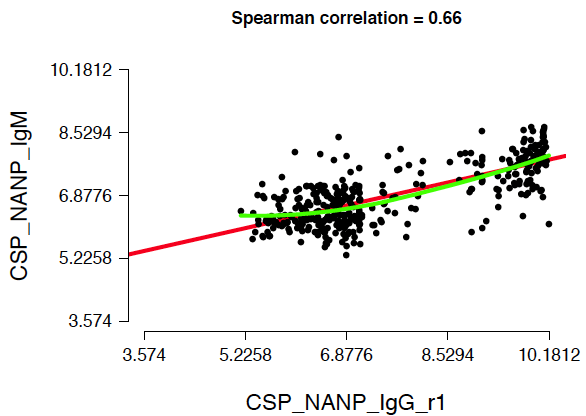

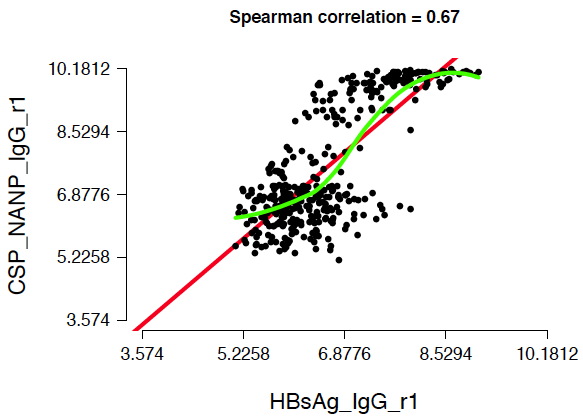
**

**G) H)**

**
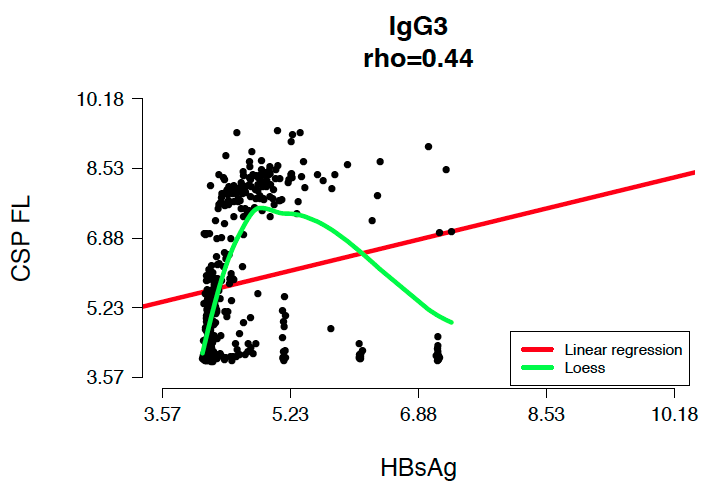

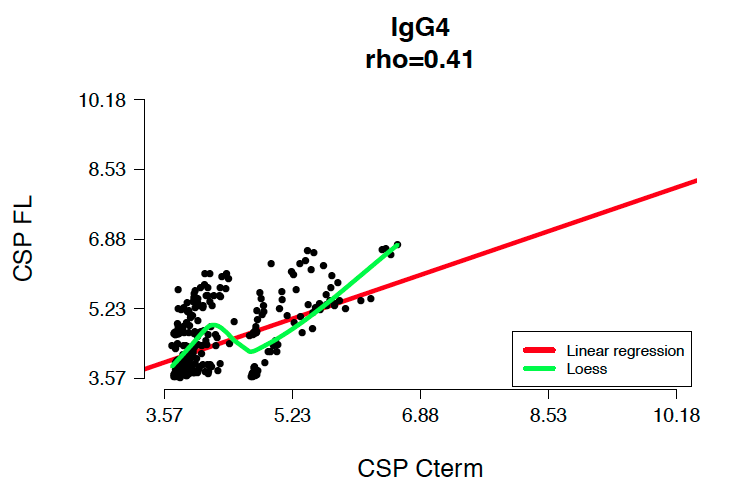
**

**Figure S3. Representative examples of scatterplots from correlation analysis.** All individuals and time points**. (A)** IgG *vs* IgG1 for CSP FL. (**B)** CSP C-term *vs* NANP for IgG1. (**C)** IgG1 *vs* IgG2 for CSP NANP. (**D)** CSP C-term *vs* NANP for IgG2. (**E)** IgG *vs* IgM for CSP NANP. (**F)** CSP NANP *vs* HBsAg for IgG in RTS,S M3. (**G)** HBsAg *vs* CSP-FL IgG3. (**H)** CSP FL *vs* C-term IgG4. In G) and H) the association between antigens had questionable monotonicity and thus correlation coefficients shall be interpreted with caution. Rho: Spearman correlation coefficient.

**A**

**
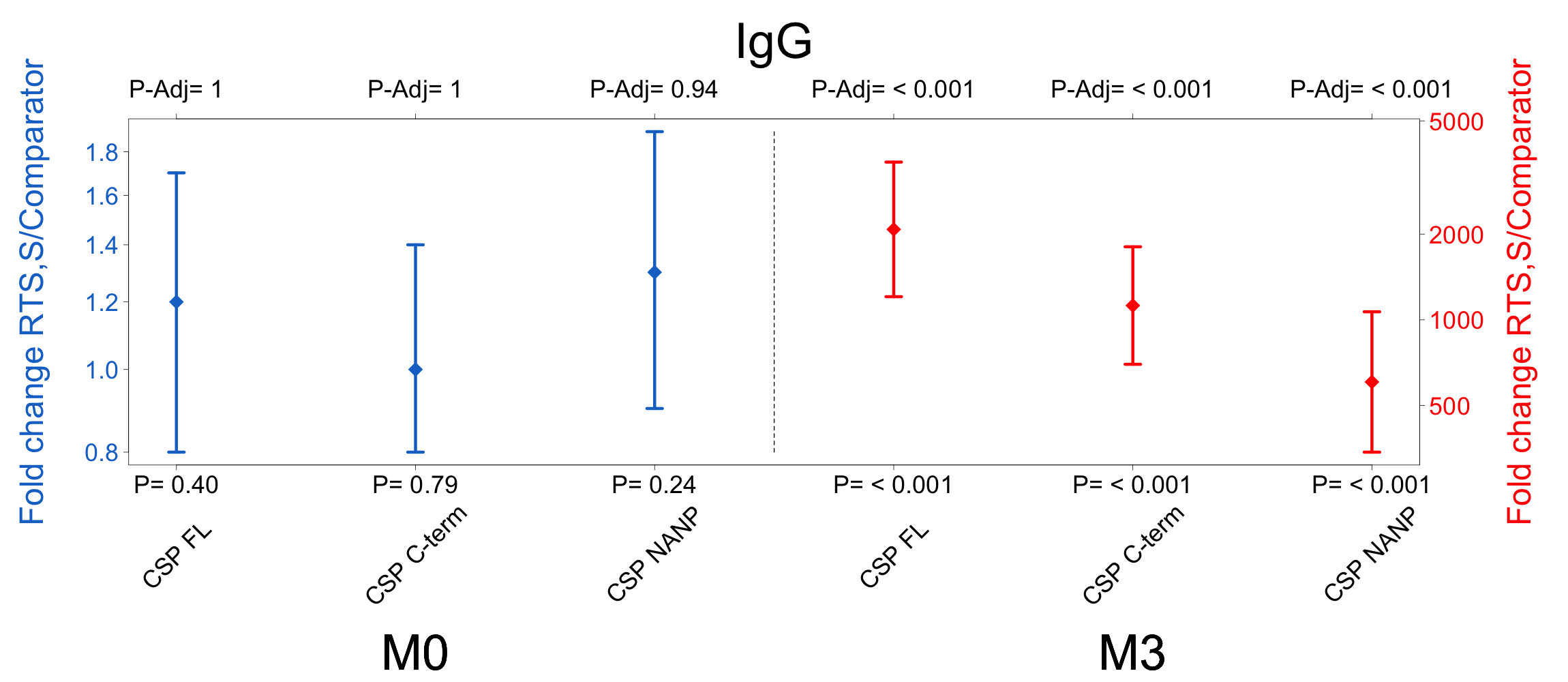
**

**B**

**
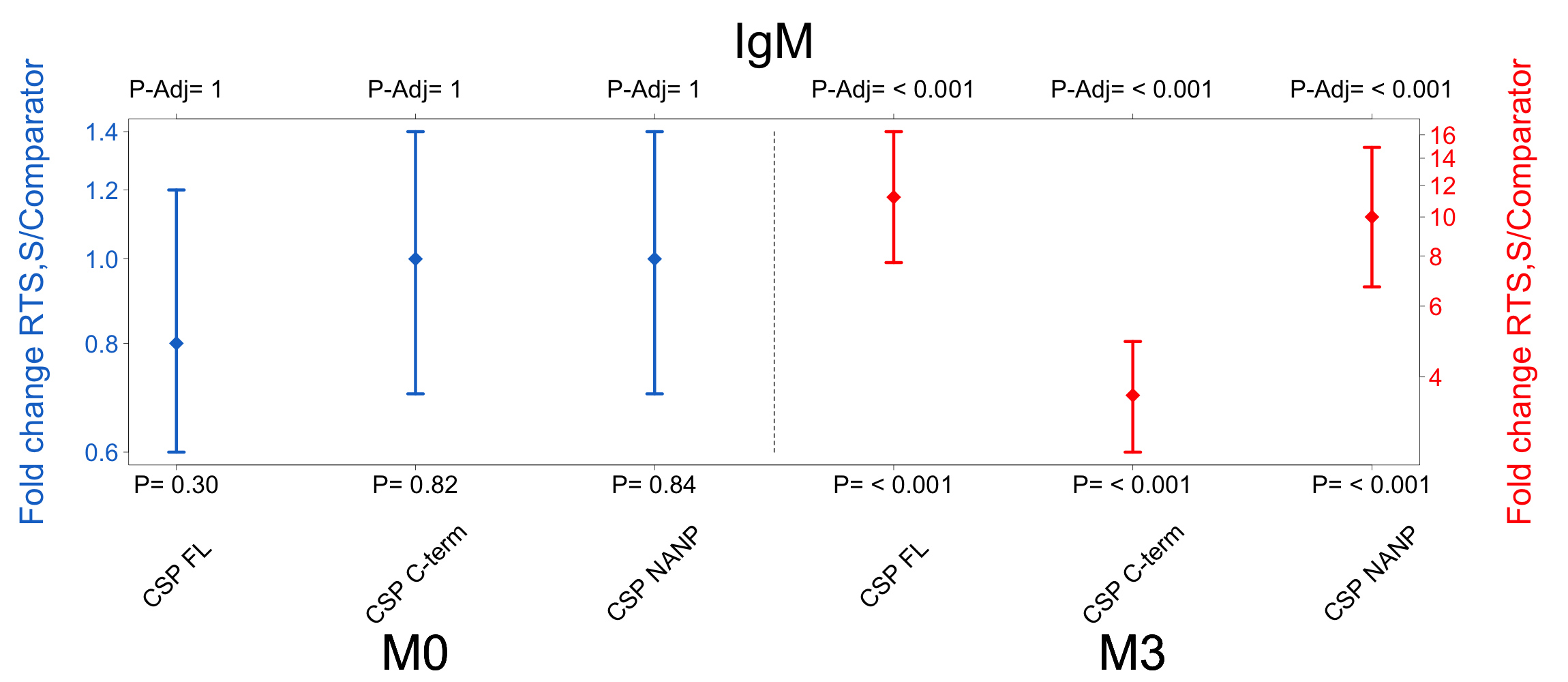
**

**C**

**
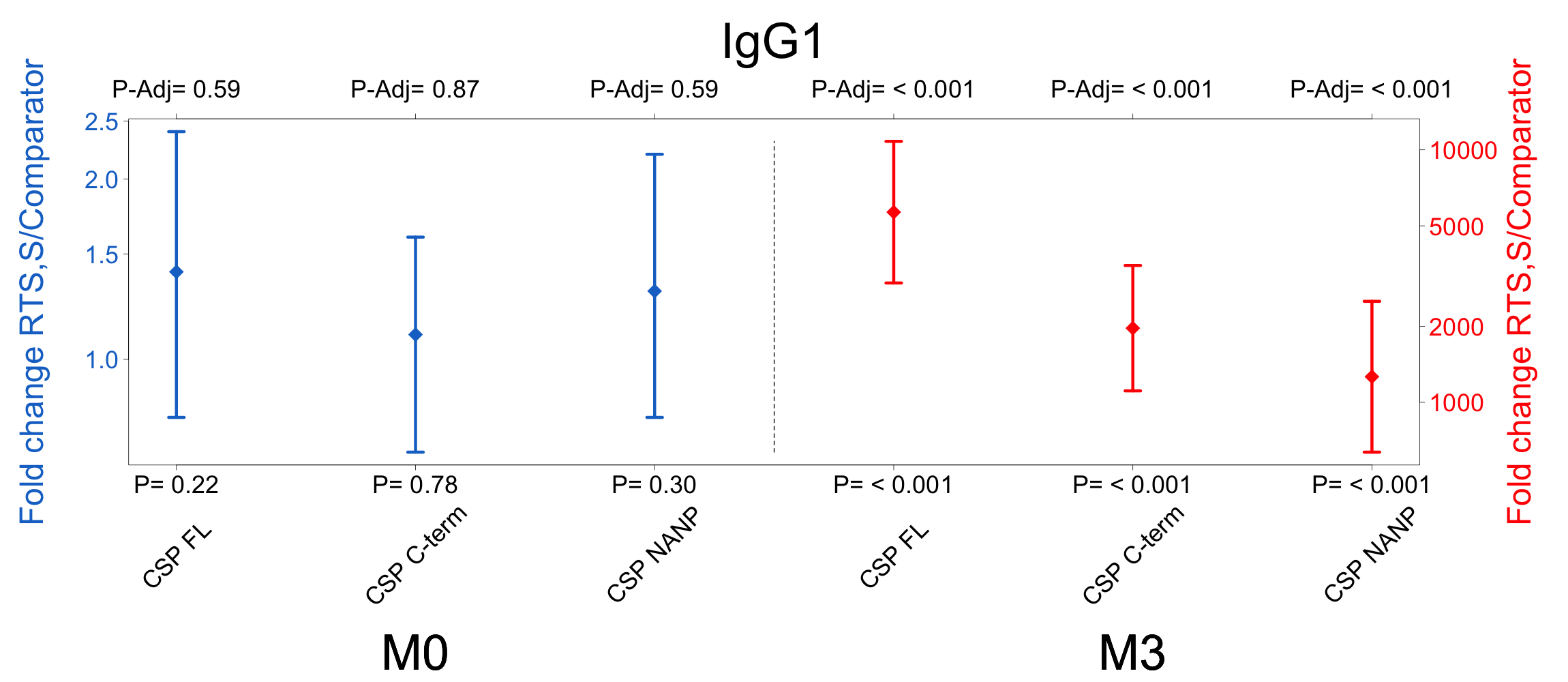
**

**D**

**
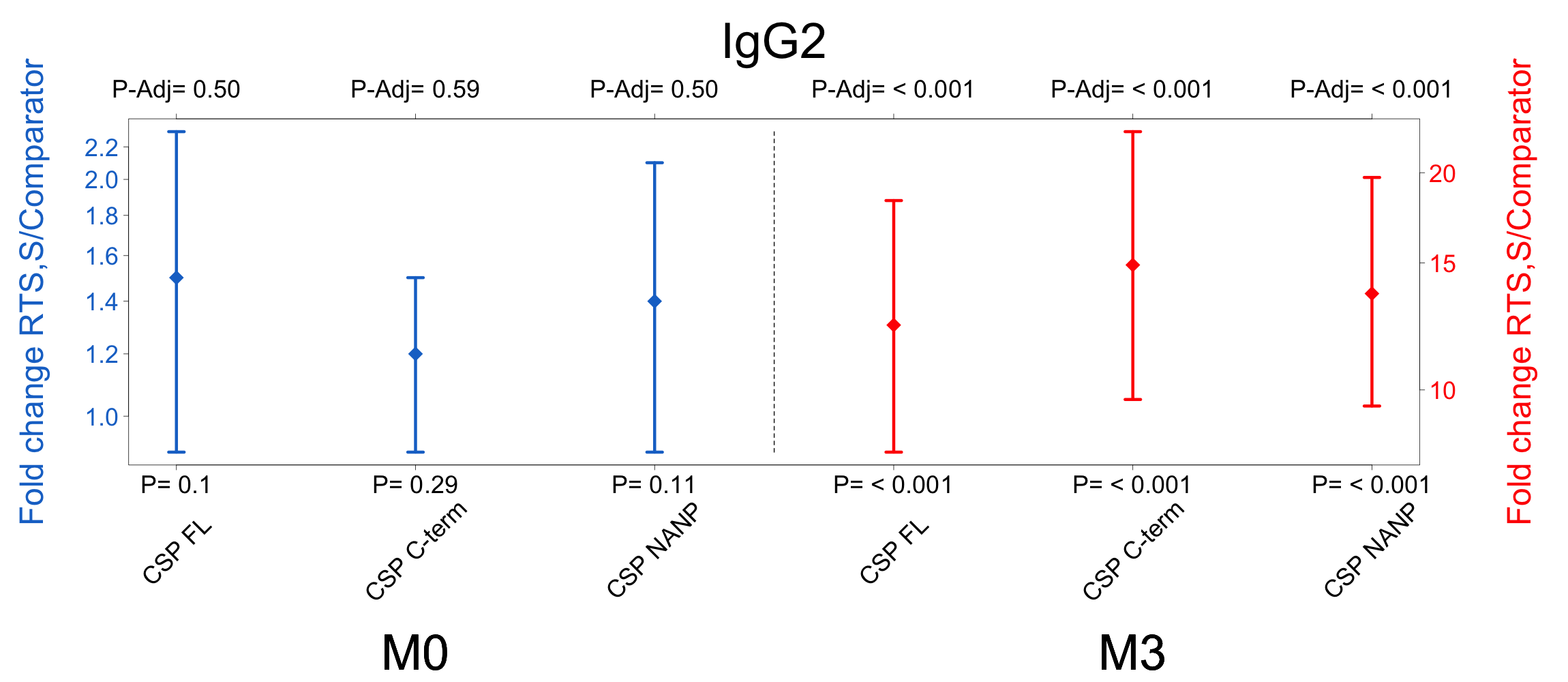
**

**E**

**
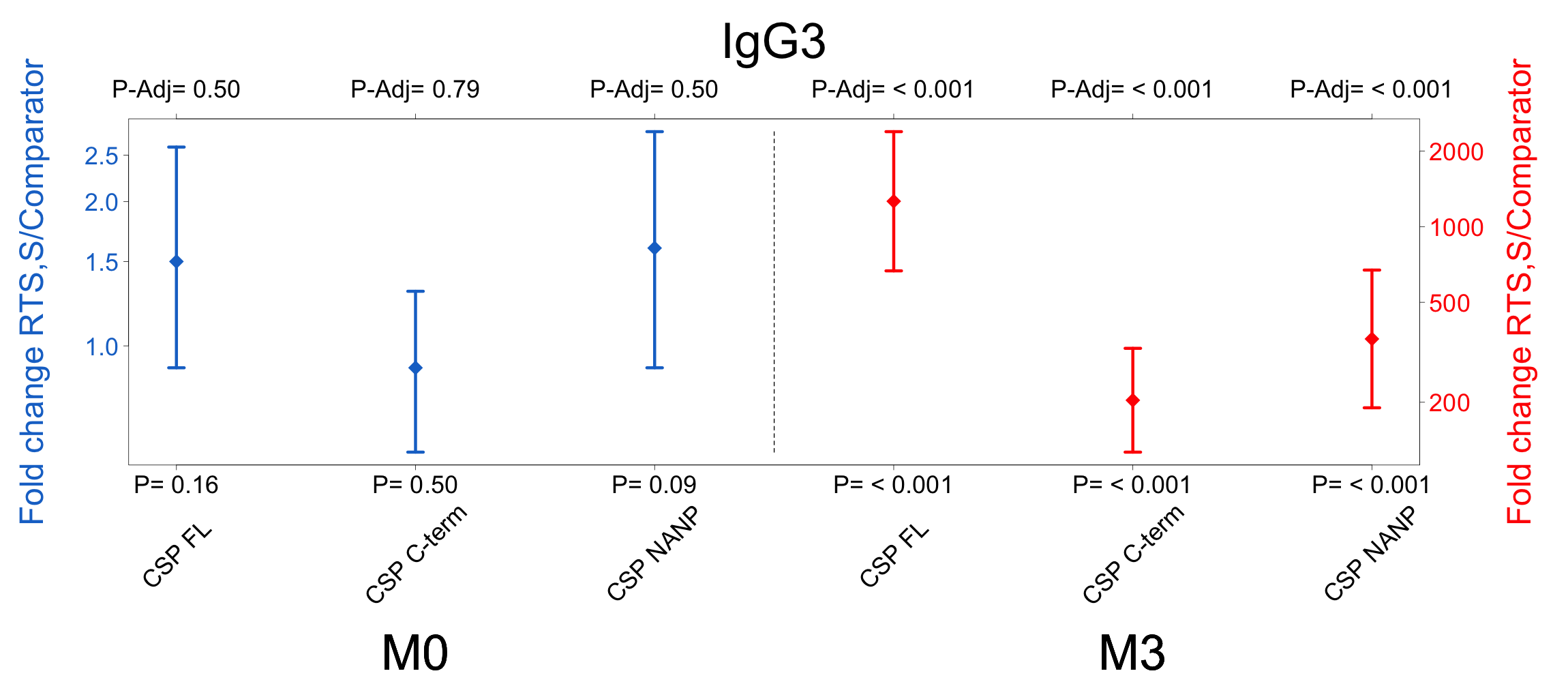
**

**F**

**
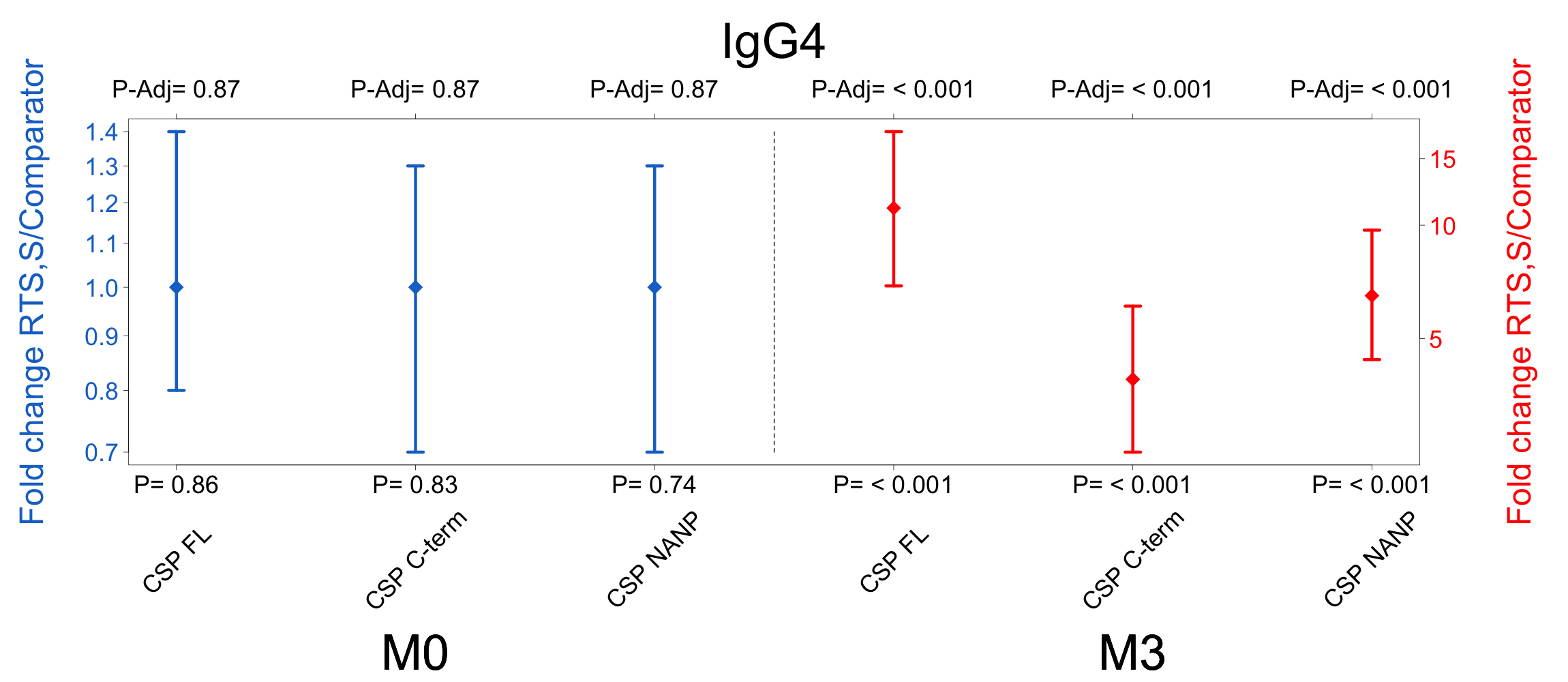
**

**G**

**
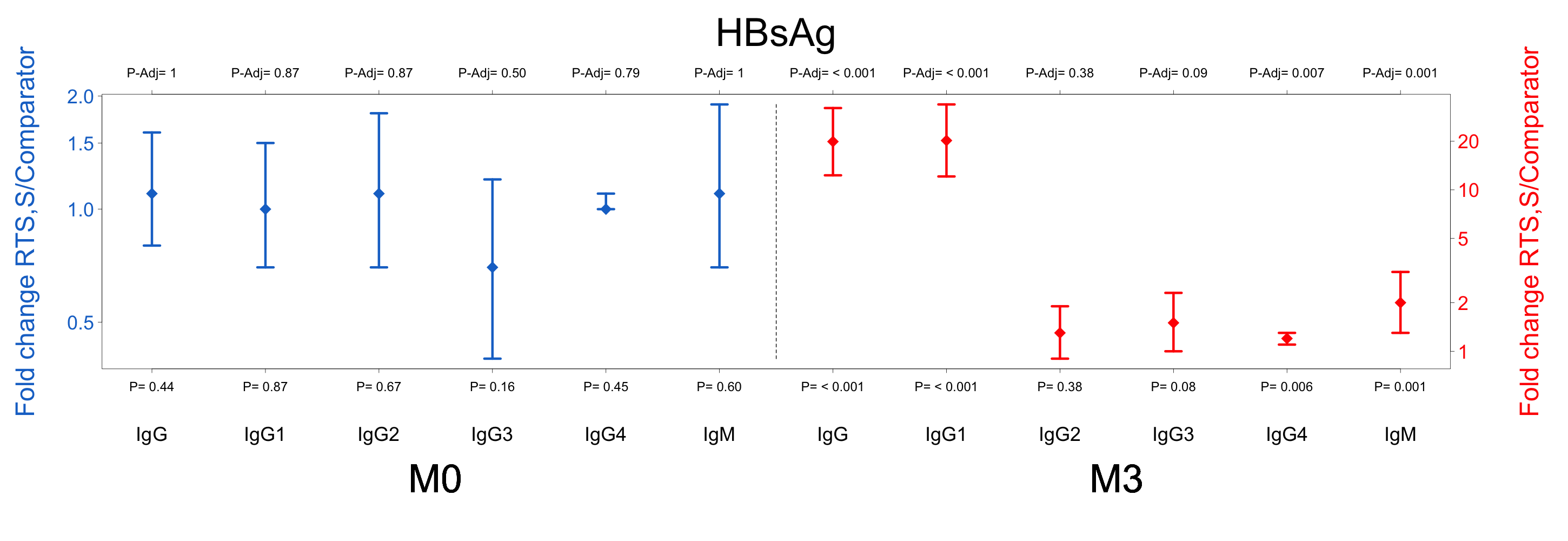
**

**Figure S4. Ratios between mean log_10_MFI antibodies in RTS,S/AS01E- versus comparator-vaccinees before (M0) and after (M3) vaccination. (A)** IgG CSP **(B)** IgM CSP **(C)** IgG1 CSP **(D)** IgG2 CSP **(E)** IgG3 CSP **(F)** IgG4 CSP **(G)** all Ig isotypes/subclasses to HBsAg. P-values obtained in mixed models adjusting for study site were corrected for multiple testing (P-Adj) through Holm for IgG and IgM across antigens within each isotype, and Benjamin-Hochberg for the IgG_1-4_ subclasses across all antigens/subclasses. Note the different scale in M0 (left) vs M3 (right). Diamonds represent the coefficient of the regression and the error bars the 95% confidence interval of the coefficients.

**CSP FL IgG IgG1 IgG2 IgG3 IgG4 IgM**

**
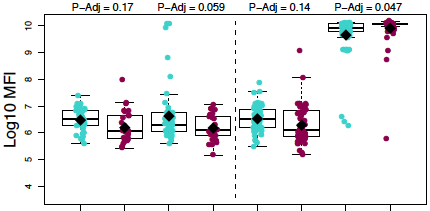
** **
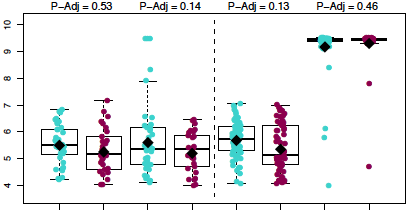
**
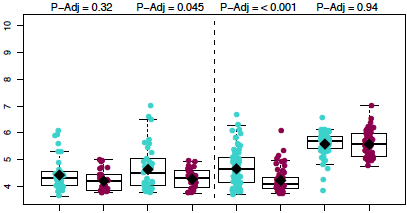

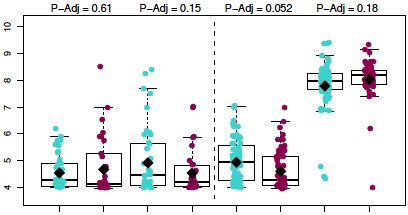

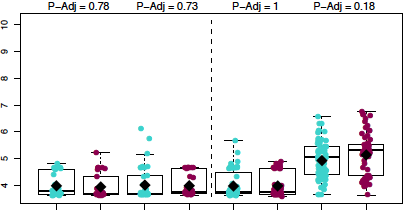

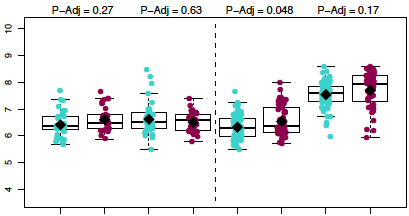


**CSP NANP**

**
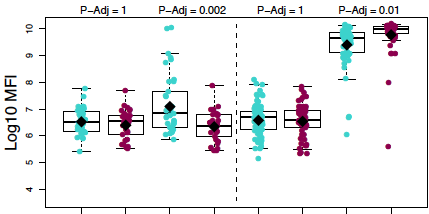
** **
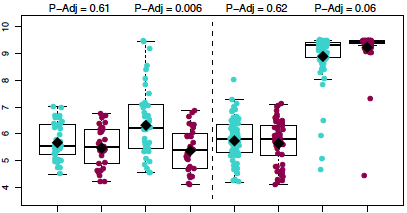
**
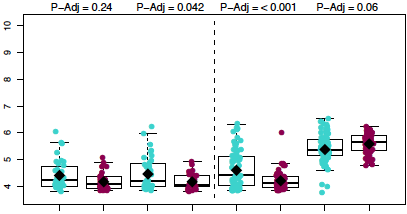

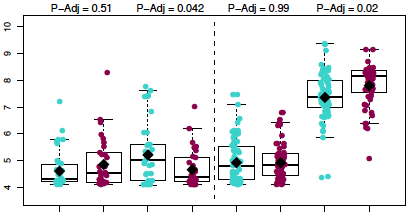

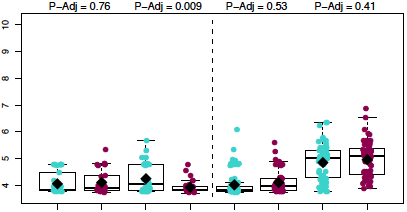

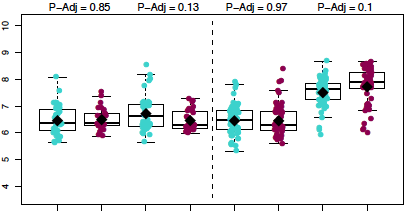


**CSP C-term**

**
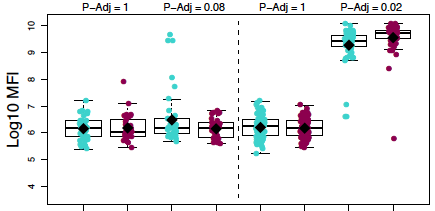
** **
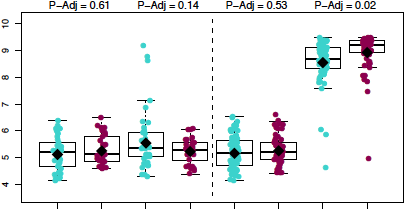
**
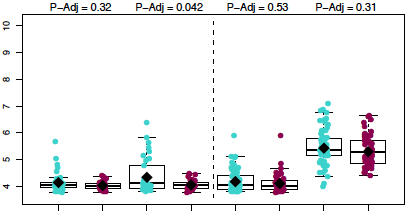

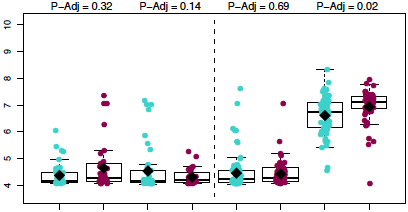

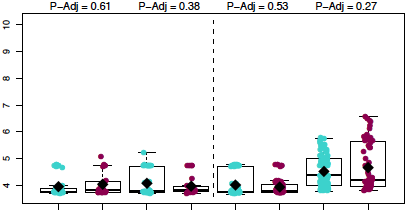

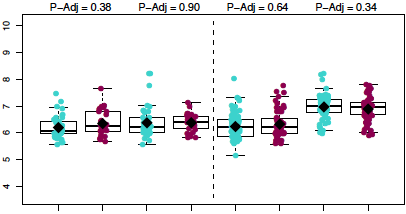


**HBsAg**

**
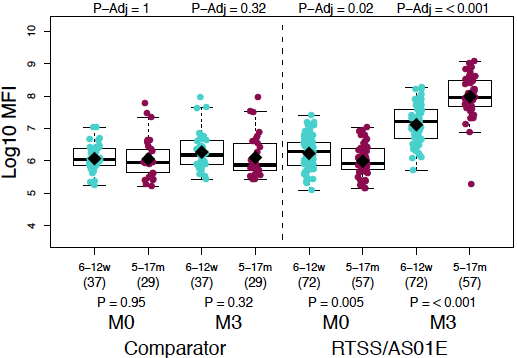
** **
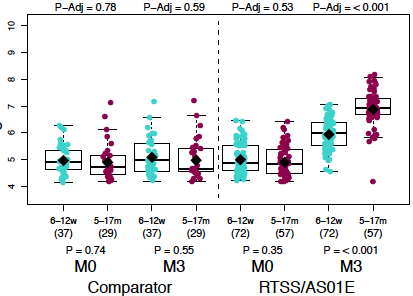
**
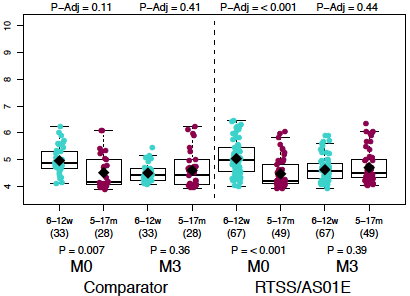

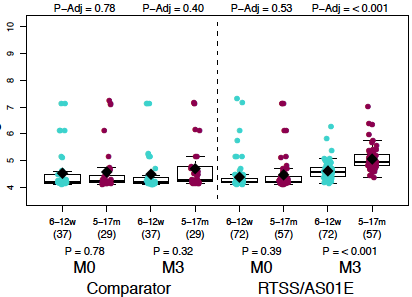

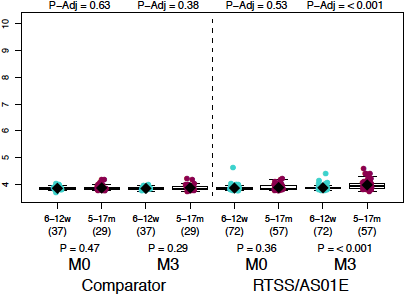

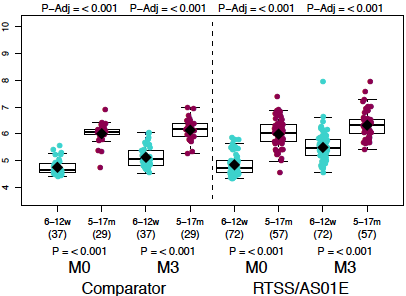


**Figure S5. Levels of antibodies to RTS,S/AS01E antigens by age cohort.** Before (M0) and after (M3) vaccination with RTS,S/AS01E or comparator stratified. Children (5-17 months old) and infants (6-12 weeks old) at baseline. Groups were compared through t-tests and p-values adjusted by Holm for IgG and IgM and by Benjamini-Hochberg for IgG_1-4_

**D) IgM CSP-FL**

**B) IgG CSP-C-term**

**C) IgG CSP-NANP**

**A) IgG CSP-FL**

**E) IgM CSP-C-term**

**F) IgM CSP-NANP**

**G) IgG1 CSP-FL**

**H) IgG1 CSP C-term**

**I) IgG1 CSP-NANP**

**J) IgG3 CSP-FL**

**K) IgG3 CSP-C-term**

**L) IgG3 CSP-NANP**

**N) IgG2 CSP-C-term**

**O) IgG2 CSP-NANP**

**M) IgG2 CSP-FL**

**R) IgG3 CSP-NANP**

**Q) IgG3 CSP-C-term**

**P) IgG3 CSP-FL**

**S) IgG HBS**

**V) IgM HBS**

**T) IgG1 HBS**

**U) IgG2 HBS**

**X) IgG3 HBS**

**Y) IgG4 HBS**

**Figure S6.** **Crude trends in antibody levels from pre- to post-vaccination.** RTS,S/AS01E vaccinated children (dashed blue line) and infants (solid blue line); comparator children (dashed red line) and infants (solid red line). Confidence intervals of pre-vaccination levels for each subgroup are shown.

**P-values for comparing trends from pre- to post-vaccination between RTS,S and comparator vaccinees, children and infants, and differences in the trends between RTS,S and comparator vaccinees across age groups:**

|  | **INFANTS** | | | **CHILDREN** | | |  |  |
| --- | --- | --- | --- | --- | --- | --- | --- | --- |
| **Antibody** | **Change in Post- / Unit Change in Pre-vaccination** | **P** | **P-Adjust** | **Change in Post- / Unit Change in Pre-vaccination** | **P** | **P-Adjust** | **P-testing Impact of Age Pre- with Post-Vaccination Association** | **P-Adjust** |
| IgG |  |  |  |  |  |  |  |  |
| FL | -0.02 | 0.93 | 1 | -0.18 | 0.11 | 0.34 | 0.45 | 0.73 |
| Cterm | -0.32 | 0.07 | 0.20 | 0.01 | 0.98 | 0.98 | 0.24 | 0.73 |
| NANP | **-0.36** | **0.01** | **0.05** | -0.14 | 0.35 | 0.71 | 0.28 | 0.73 |
| IgM |  |  |  |  |  |  |  |  |
| FL | -0.08 | 0.55 | 1 | 0.29 | 0.07 | 0.21 | 0.07 | 0.21 |
| Cterm | -0.03 | 0.82 | 1 | 0.2 | 0.09 | 0.21 | 0.16 | 0.31 |
| NANP | -0.09 | 0.44 | 1 | 0.17 | 0.24 | 0.24 | 0.16 | 0.31 |
| IgG1 |  |  |  |  |  |  |  |  |
| FL | 0.178 | 0.25 | 0.50 | -0.11 | 0.29 | 0.65 | 0.12 | 0.40 |
| Cterm | -0.075 | 0.63 | 0.71 | 0.11 | 0.55 | 0.69 | 0.46 | 0.77 |
| NANP | **-0.296** | **0.04** | 0.20 | -0.14 | 0.24 | 0.65 | 0.40 | 0.77 |
| IgG2 |  |  |  |  |  |  |  |  |
| FL | 0.127 | 0.1 | 0.31 | **0.45** | **0.002** | **0.01** | 0.04 | 0.21 |
| Cterm | 0.281 | 0.15 | 0.35 | 0.19 | 0.44 | 0.69 | 0.76 | 0.87 |
| NANP | **0.251** | **0.008** | 0.07 | 0.13 | 0.37 | 0.65 | 0.53 | 0.77 |
| IgG3 |  |  |  |  |  |  |  |  |
| FL | 0.06 | 0.65 | 0.71 | 0.08 | 0.56 | 0.69 | 0.93 | 0.96 |
| Cterm | 0.044 | 0.72 | 0.72 | -0.12 | 0.49 | 0.69 | 0.45 | 0.77 |
| NANP | -0.127 | 0.32 | 0.56 | 0.01 | 0.94 | 0.94 | 0.48 | 0.77 |
| IgG4 |  |  |  |  |  |  |  |  |
| FL | 0.075 | 0.67 | 0.71 | 0.28 | 0.25 | 0.65 | 0.48 | 0.77 |
| Cterm | -0.28 | 0.08 | 0.31 | -0.14 | 0.72 | 0.76 | 0.70 | 0.86 |
| NANP | 0.089 | 0.58 | 0.71 | 0.07 | 0.71 | 0.76 | 0.96 | 0.96 |
| **HBsAg** |  |  |  |  |  |  |  |  |
| IgG | -0.089 | 0.51 | 1 | **0.46** | **0.01** | **0.04** | **0.01** | **0.052** |
| IgM | 0.026 | 0.87 | 1 | **0.543** | **< 0.001** | **< 0.001** | **0.007** | **0.03** |
| IgG1 | -0.188 | 0.11 | 0.31 | 0.287 | 0.07 | 0.29 | 0.40 | 0.77 |
| IgG2 | **0.565** | **< 0.001** | **< 0.001** | **0.893** | **< 0.001** | **< 0.001** | 0.53 | 0.77 |
| IgG3 | -0.038 | 0.59 | 0.71 | -0.085 | 0.33 | 0.65 | 0.48 | 0.77 |
| IgG4 | 0.071 | 0.48 | 0.71 | 0.414 | 0.057 | 0.29 | 0.12 | 0.40 |

**A**

**
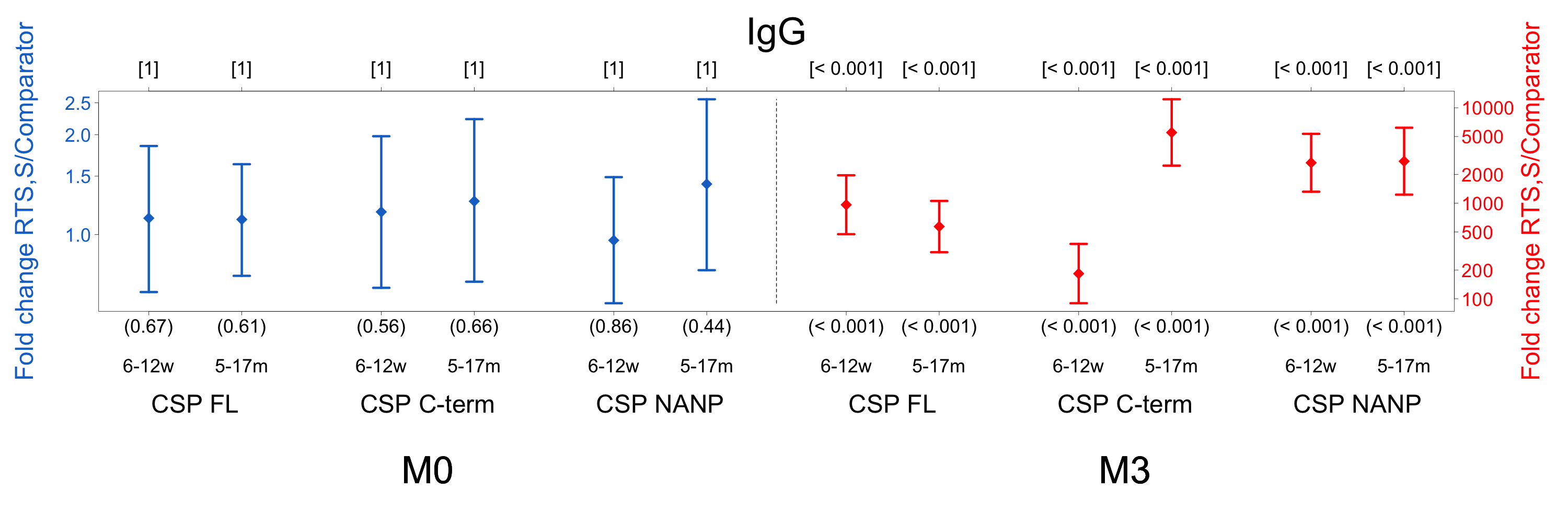
**

**B**

**
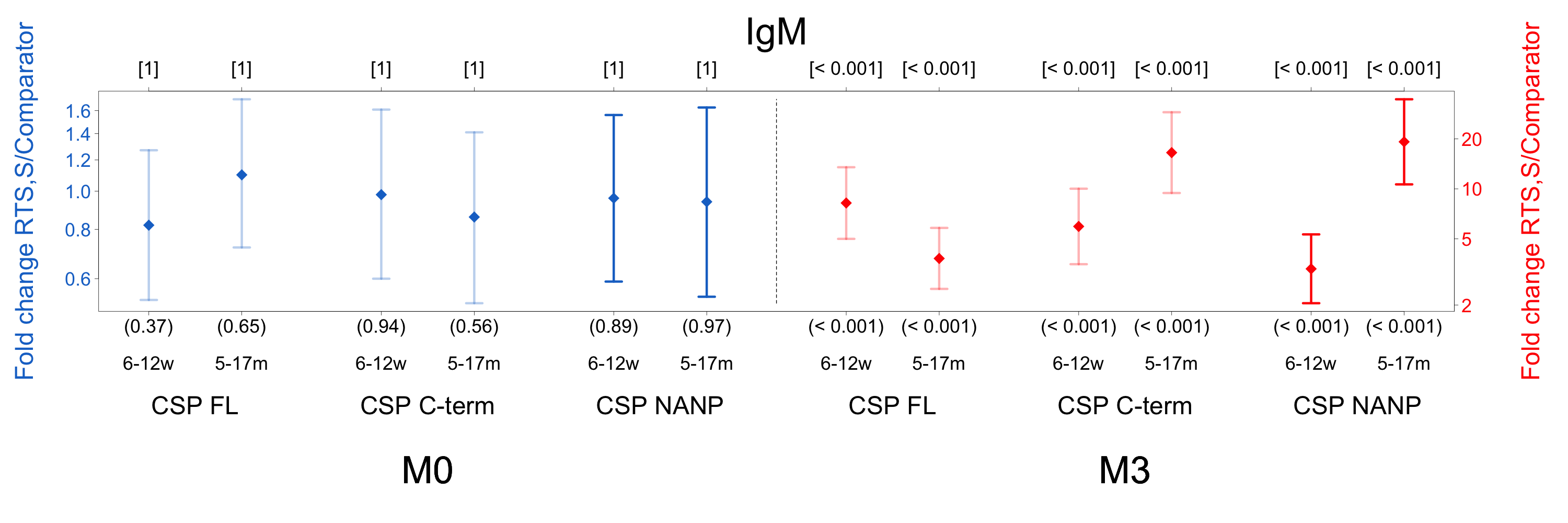
**

**C**

**
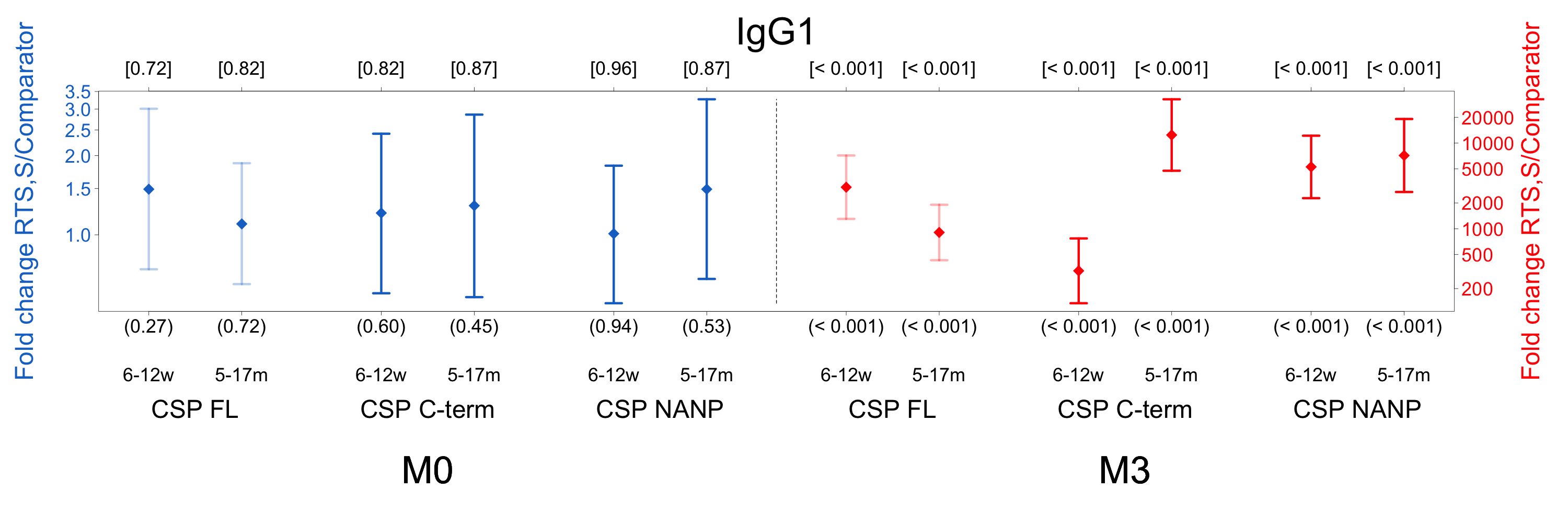
**

**D**

**
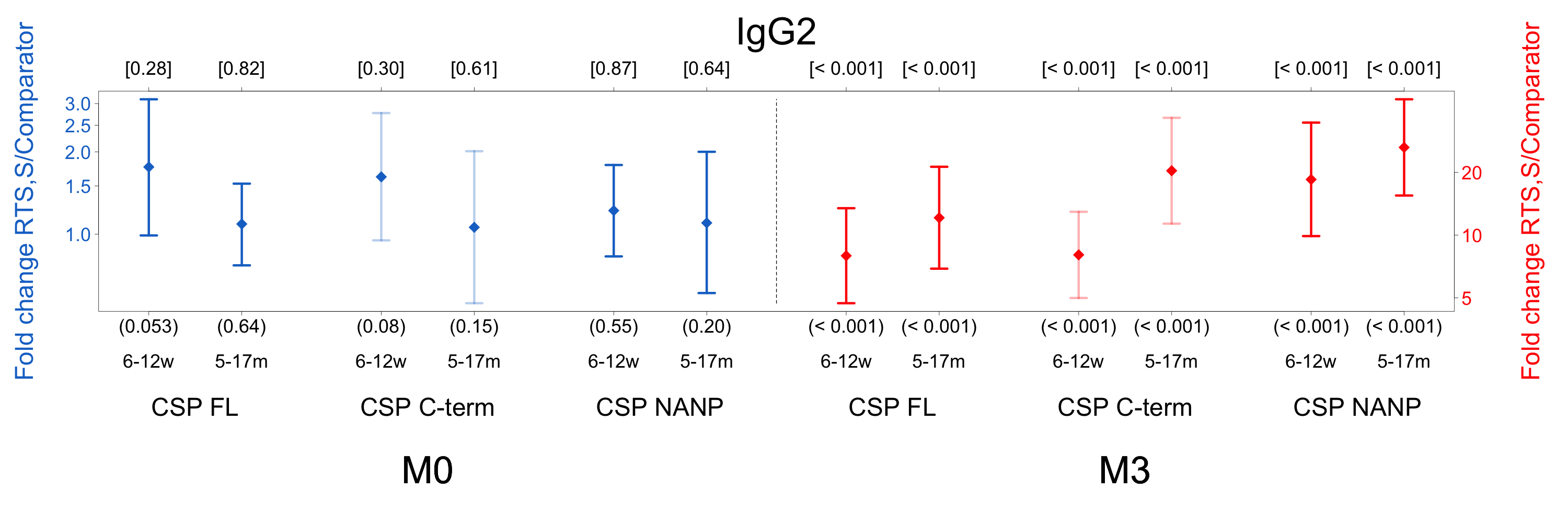
**

**E**

**
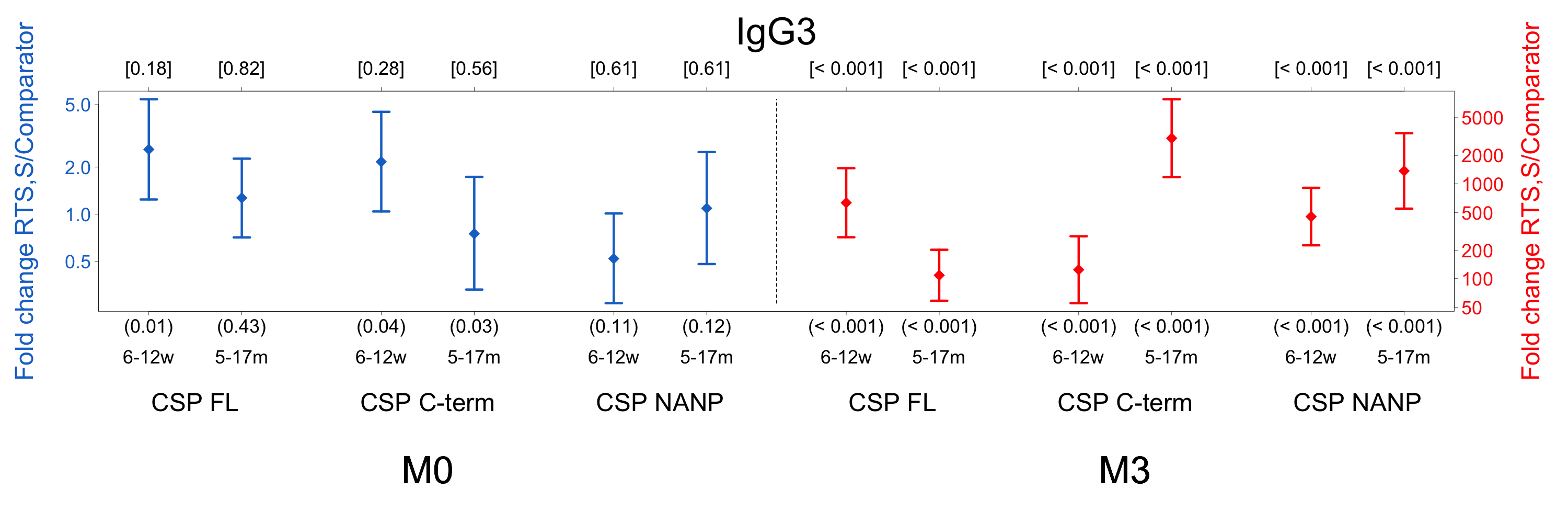
**

**F**

**
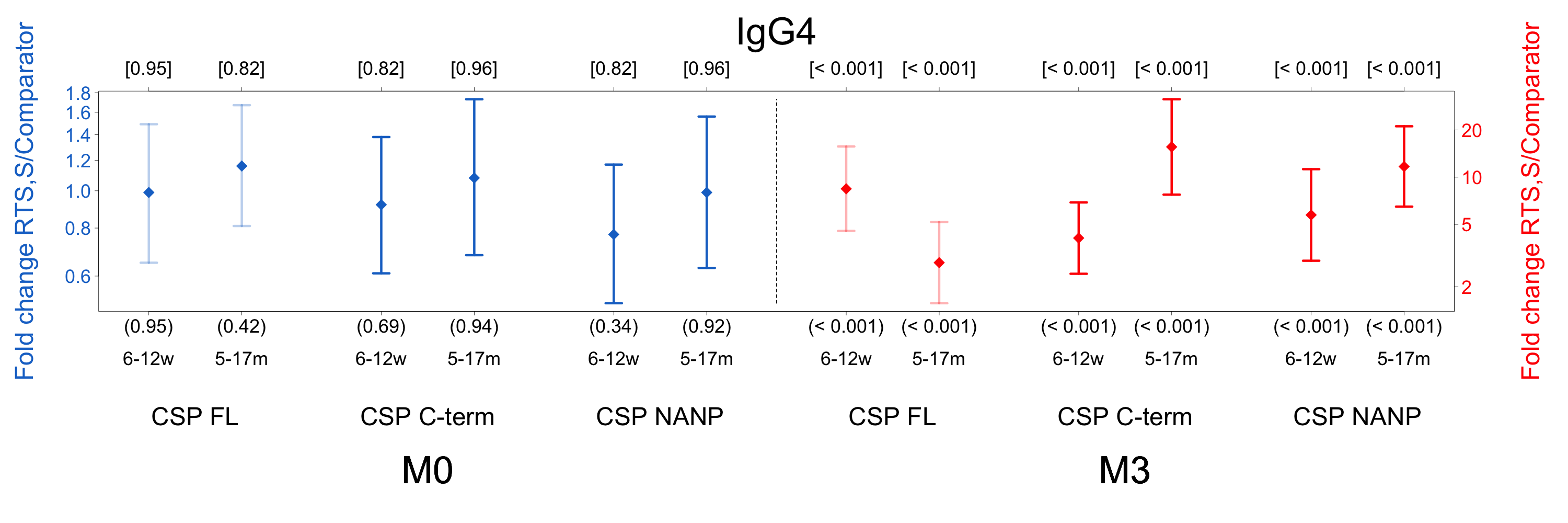
**

**G**

**
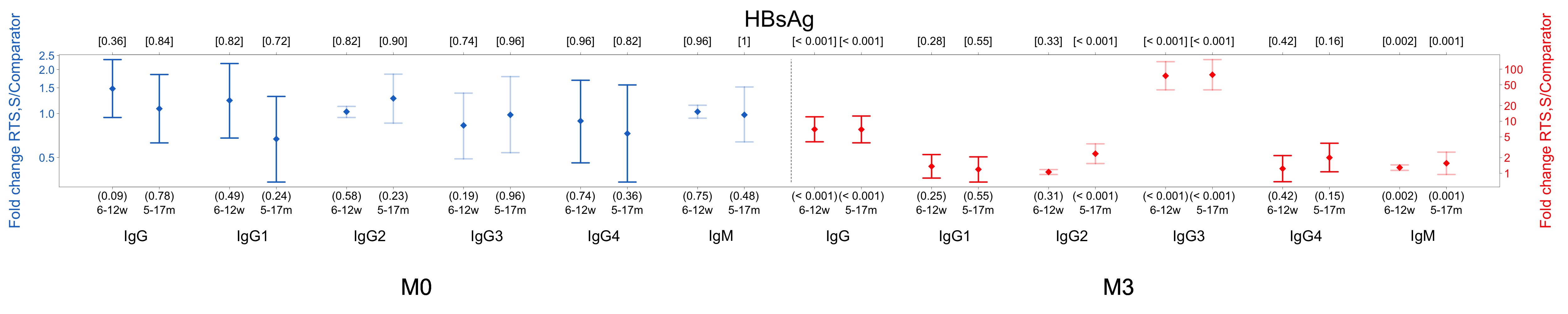
**

**Figure S7. Ratios between mean antibody levels in RTS,S/AS01E- versus comparator-vaccinees stratified by age group. (A)** IgG CSP **(B)** IgM CSP **(C)** IgG1 CSP **(D)** IgG2 CSP **(E)** IgG3 CSP **(F)** IgG4 CSP **(G)** all Ig isotypes/subclasses to HBsAg. Before (M0) and after (M3) vaccination. Antibody levels expressed as log_10_MFI. P-values obtained in mixed models adjusting for study site were corrected for multiple testing (P-Adj, top values) through Holm for IgG and IgM across antigens within each isotype, and Benjamin-Hochberg for the IgG_1-4_ subclasses across all antigens/subclasses. Note the different scale in M0 (left) vs M3 (right). Diamonds represent the coefficient of the regression and the error bars the 95% confidence interval of the coefficients.

**CSP FL IgG IgG1 IgG2 IgG3 IgG4 IgM**

**
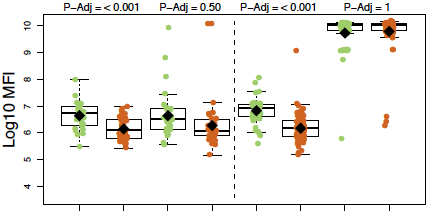

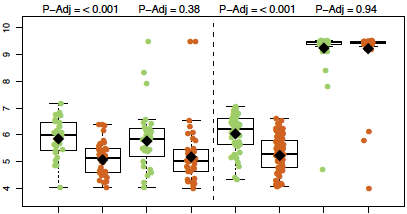

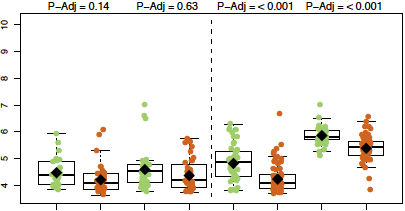

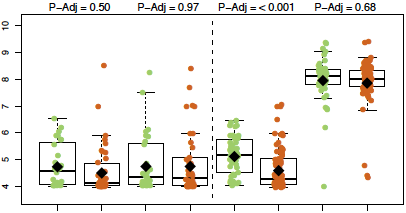

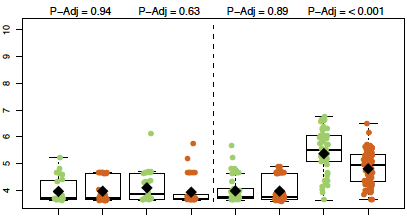

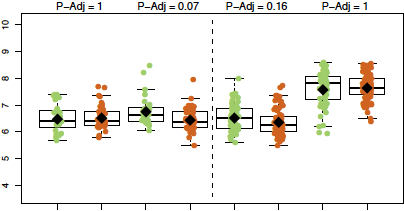
**

**CSP NANP**

**
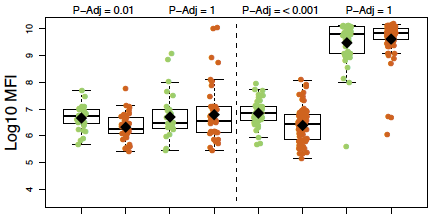

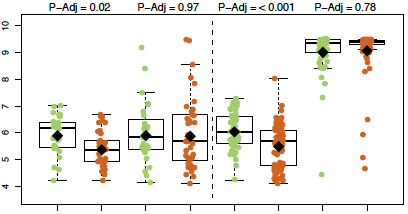

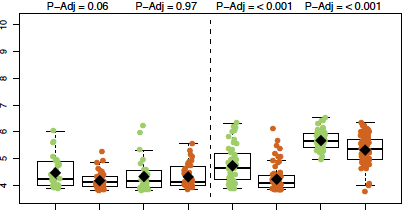

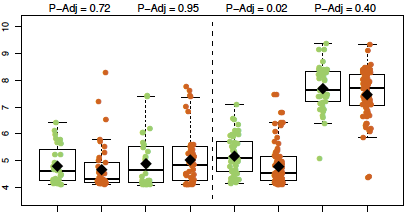

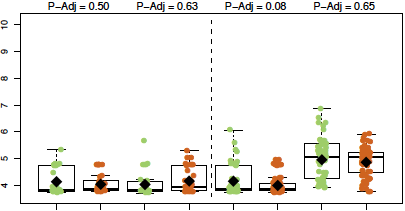

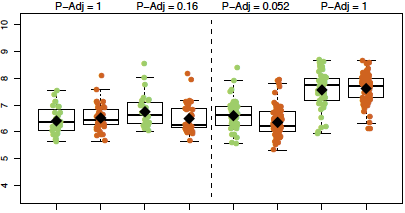
**

**CSP C-term**

**
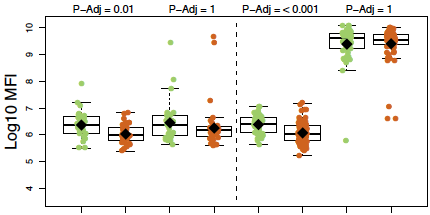

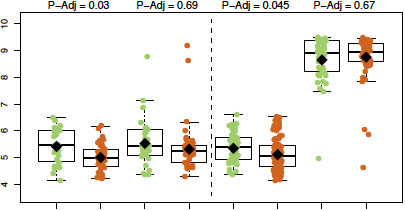

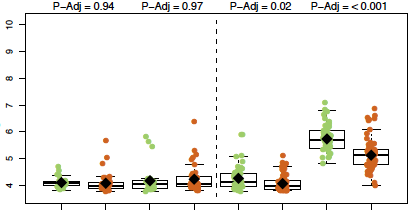

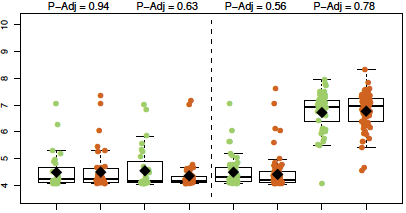

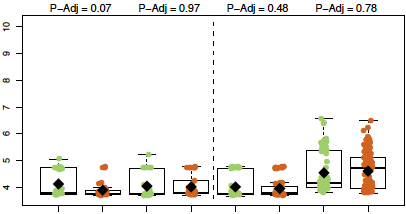

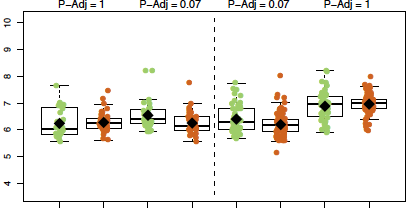
**

**HBsAg**

**
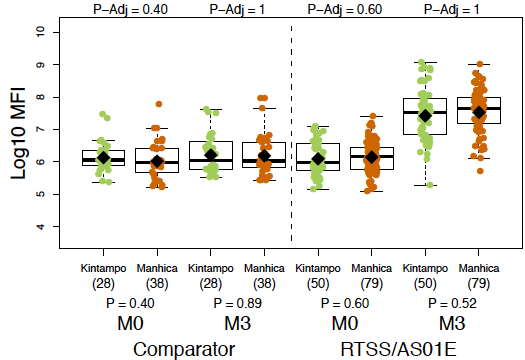

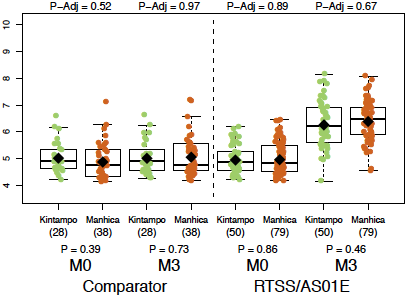

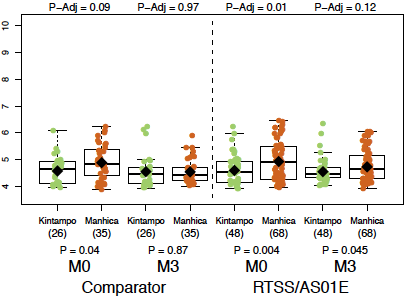

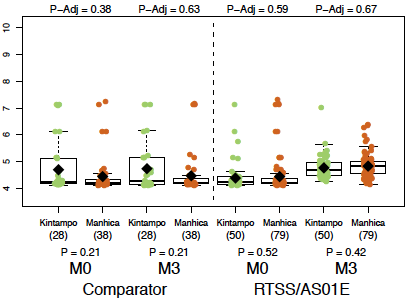

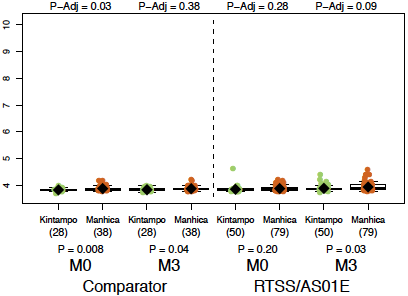

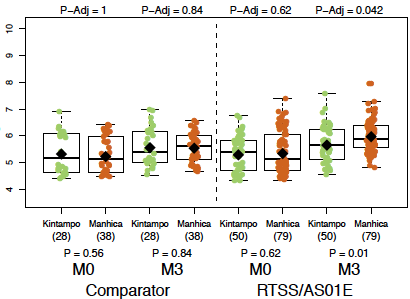
**

**Figure S8. CSP and HBsAg antibody responses in RTS,S and comparator vaccines comparing between sites.** Kintampo (higher malaria transmission intensity) and Manhiça (lower malaria transmission intensity). Groups were compared through t-tests and p-values adjusted by Holm for IgG and IgM and by Benjamini-Hochberg for IgG_1-4_, as explained in the methods section.

**CSP FL IgG IgM**

**
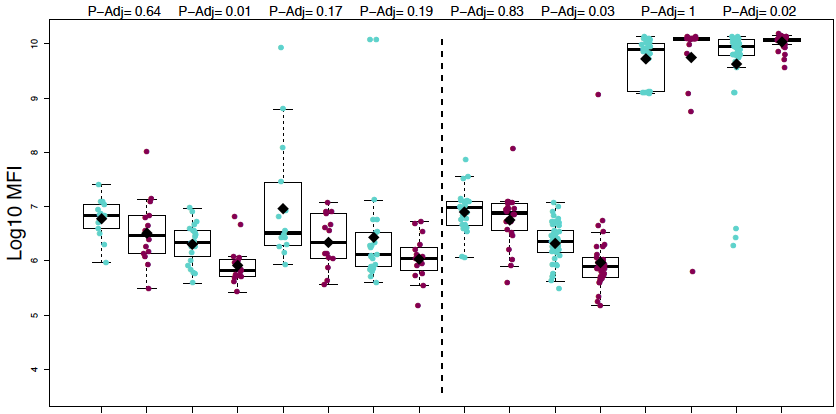
** **
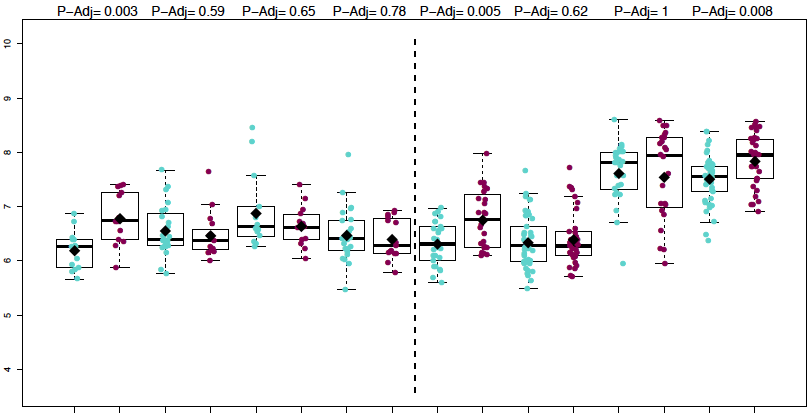
**

**IgG1 IgG3**


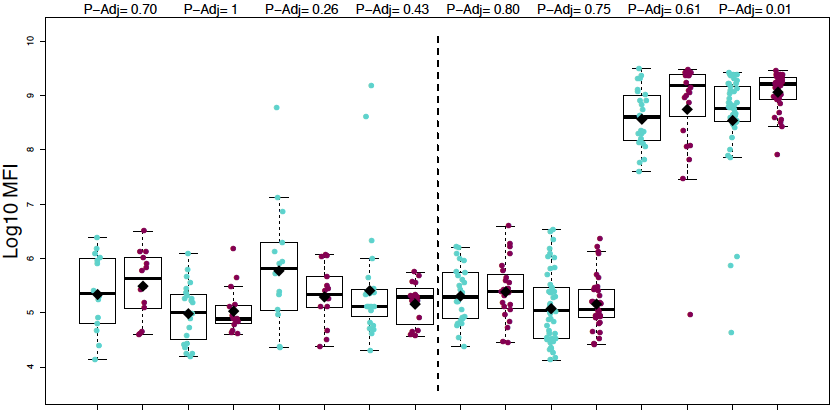

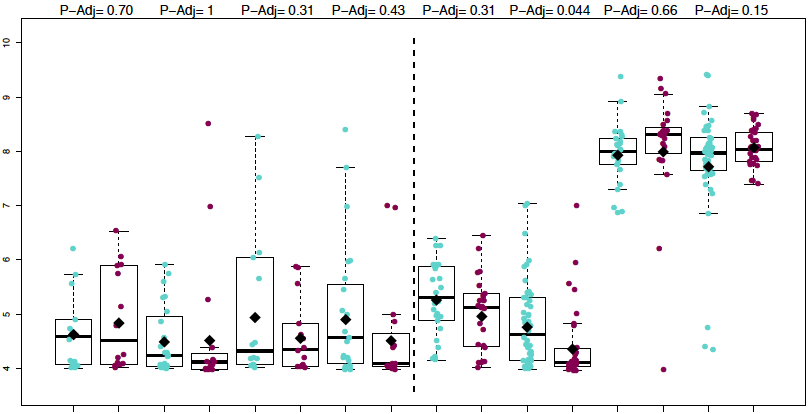


**IgG2 IgG4**


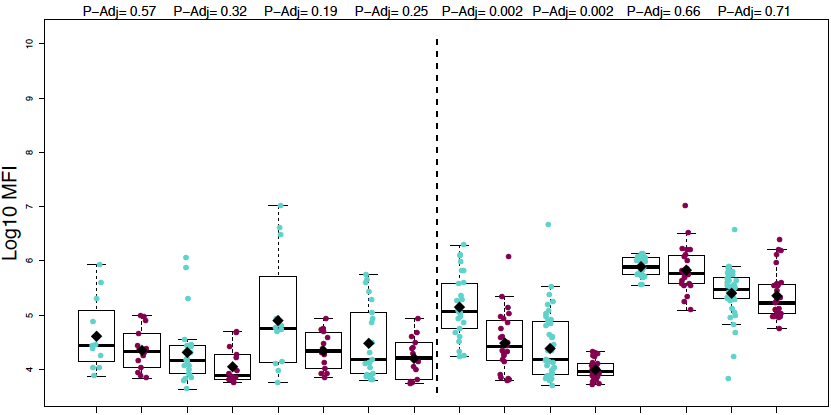

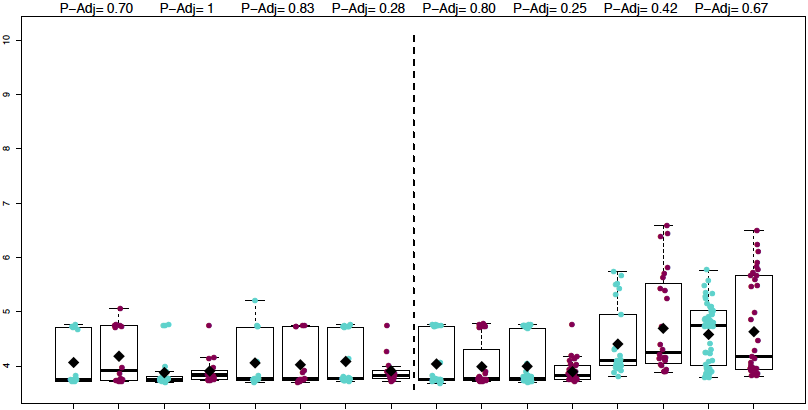


**HBsAg IgG IgM**


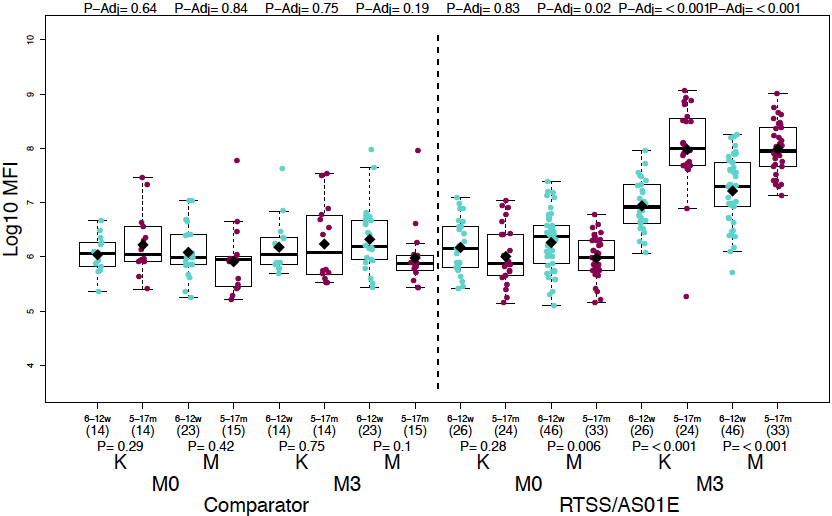

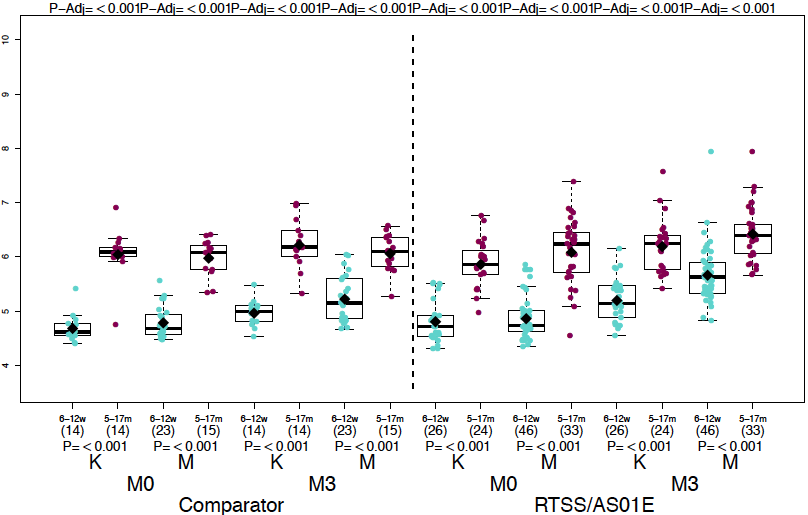


**Figure S9. CSP and HBsAg antibody responses in RTS,S and comparator vaccinees by site and comparing between age cohorts.** Selected antibodies and antigens shown as examples. Groups were compared through t-tests and p-values adjusted by Holm for IgG and IgM and by Benjamini-Hochberg for IgG_1-4_, as explained in the methods. Age group, infants (6-12w) and children (5-17m). K = Kintampo, M = Manhiça.

**A**


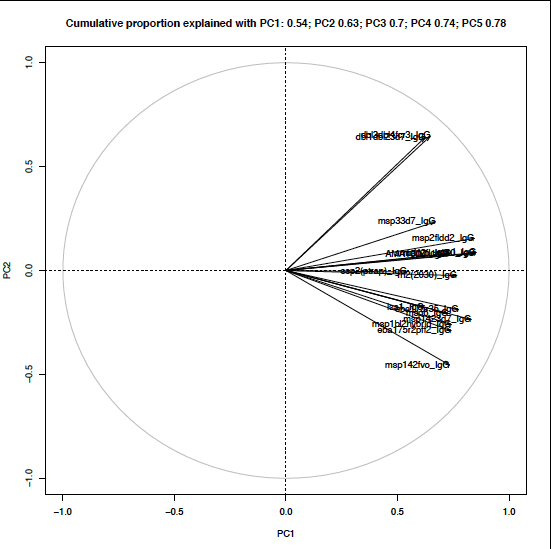


**B**


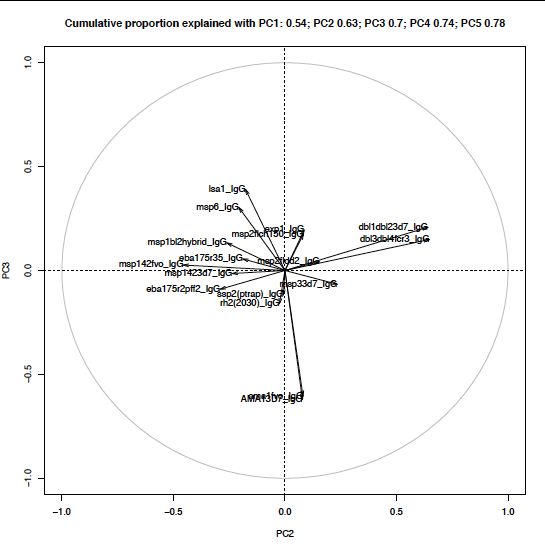


**C**


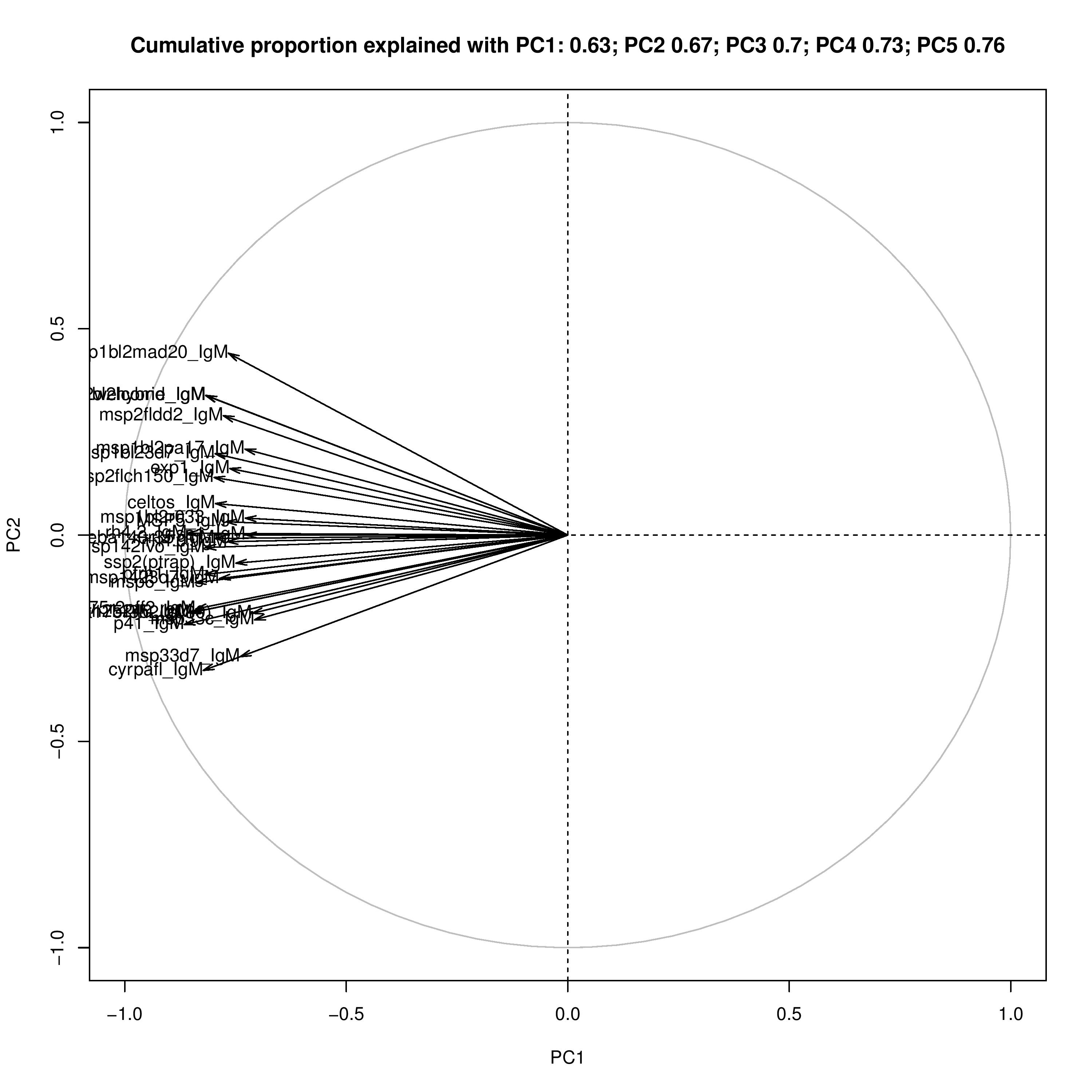


**D**


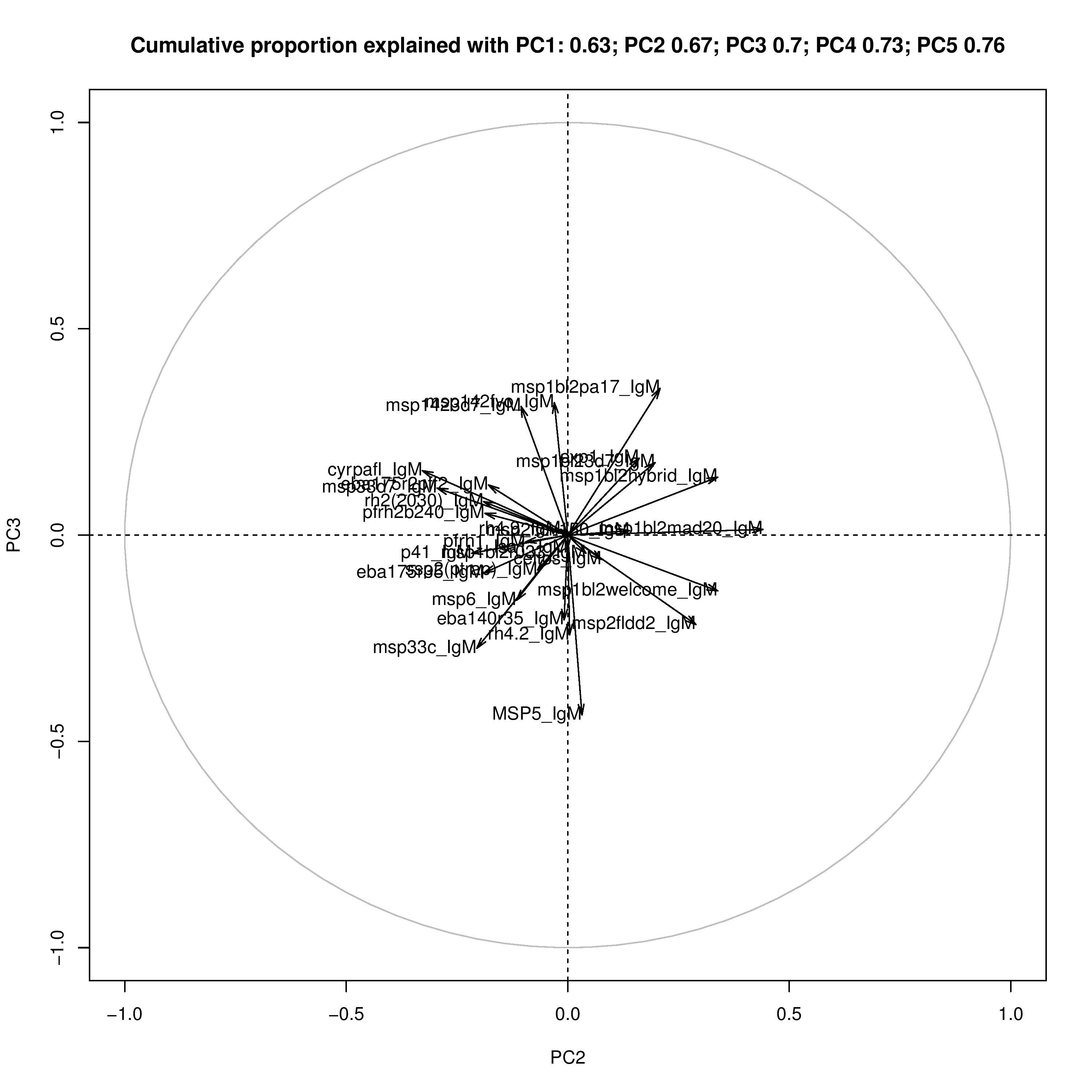


**E**


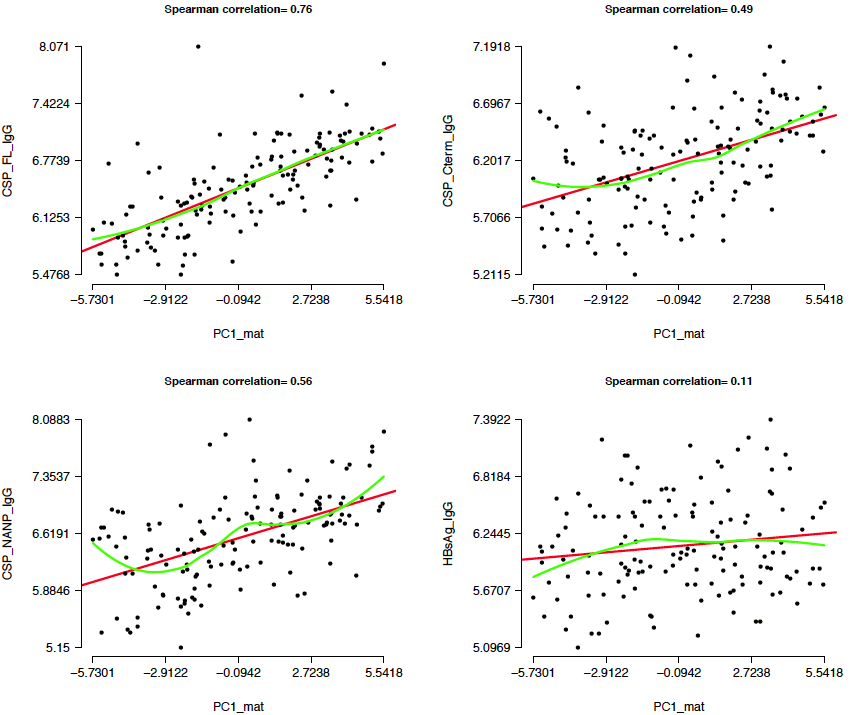


**F**


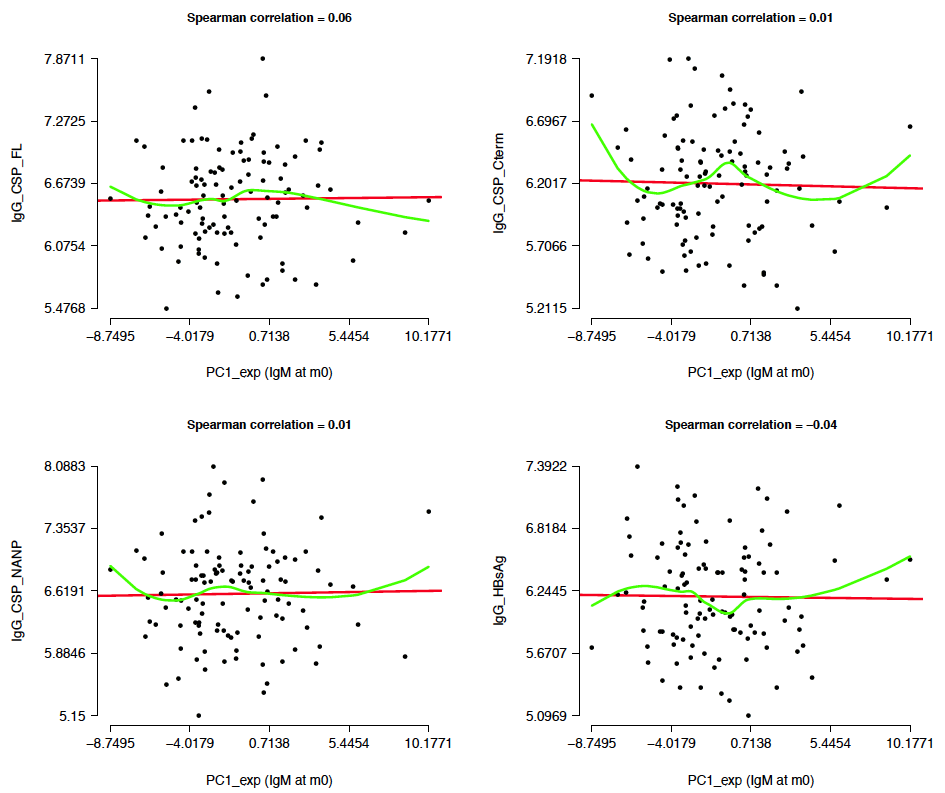


**G**


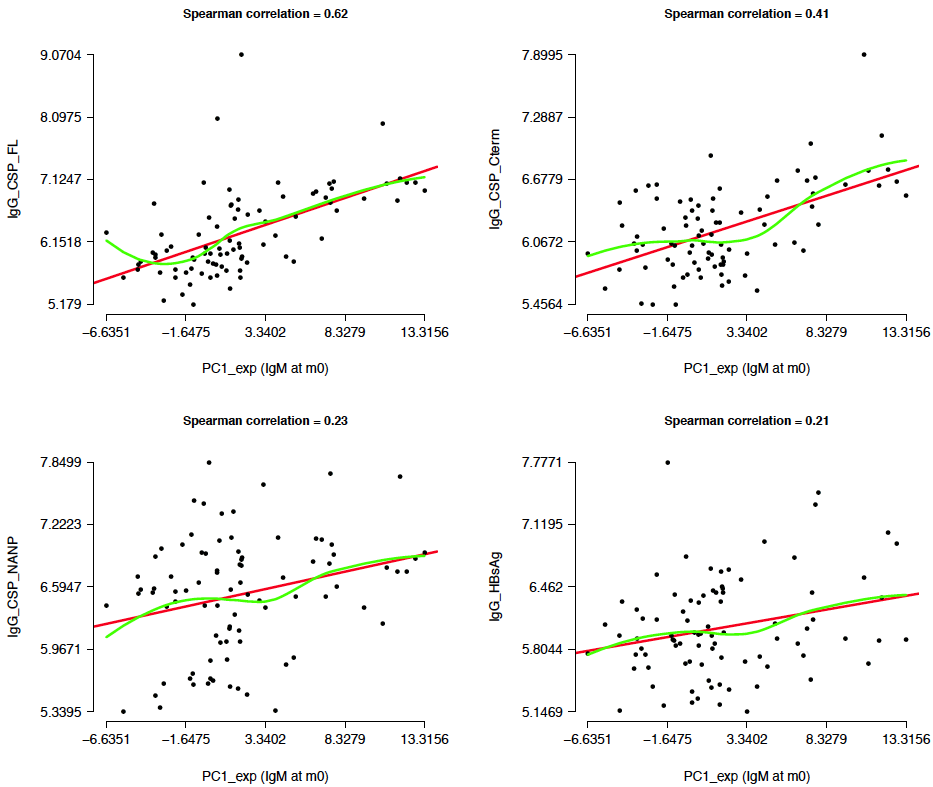


**H**

**
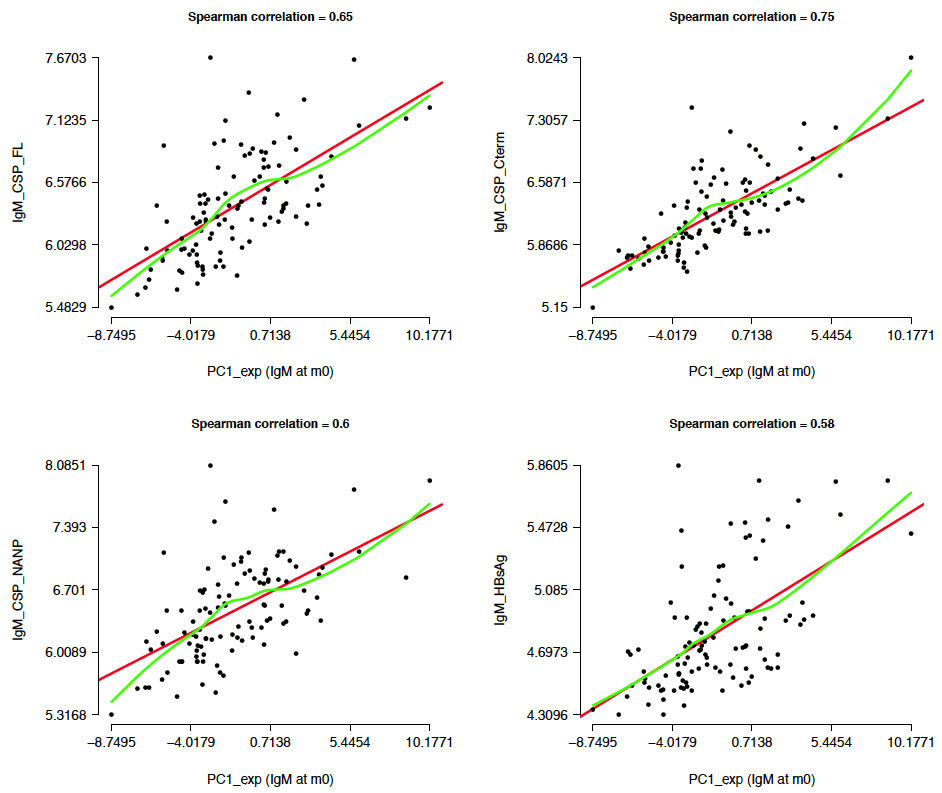
**

**I**

**
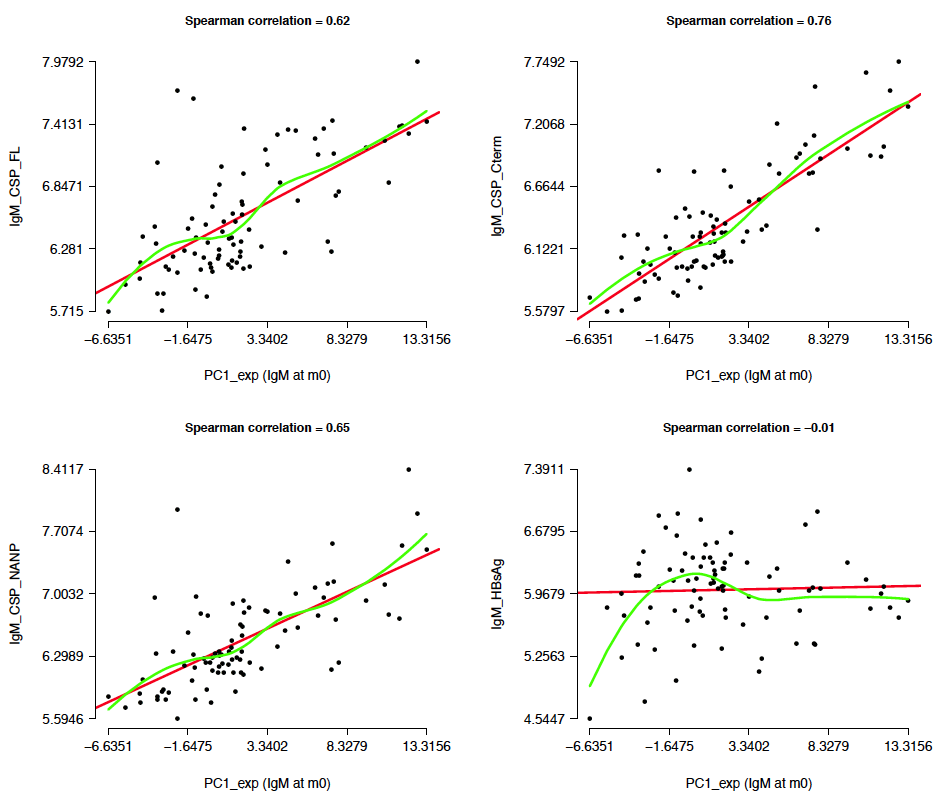
**

**Figure S10.** **Markers of antimalarial maternal antibodies and exposure at baseline**. Associations between antigens in components obtained in a principal component analysis (Table S8) aiming to estimate summary scores for “maternal antibodies” and “malaria exposure” indices at pre-vaccination, and correlation with baseline CSP antibody levels. **A)** Component 1 vs component 2 for IgG maternal antibodies in infants and children <10 months at baseline. **B)** Component 2 vs component 3 for IgG maternal antibodies in infants and children <10 months at baseline. **C)** Component 1 vs component 2 for IgM exposure antibodies. **D)** Component 2 vs component 3 for IgM exposure antibodies. **E)** Correlations between baseline RTS,S Ig levels and *P. falciparum* maternal antibodies index (PC1) at baseline. **F)** Correlations between baseline RTS,S Ig levels and malaria exposure index (PC1) at baseline, IgG in infants, **G)** IgG in children, **H)** IgM in infants, **I)** IgM in children.

**CSP FL IgG IgG1 IgG2 IgG3 IgG4**

**
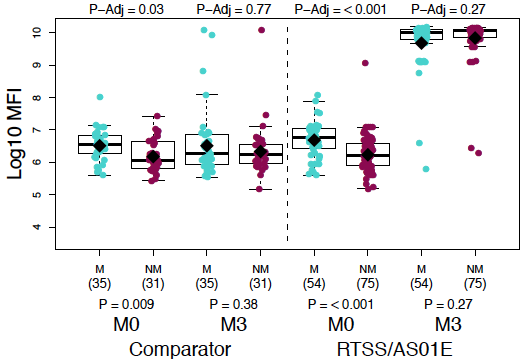

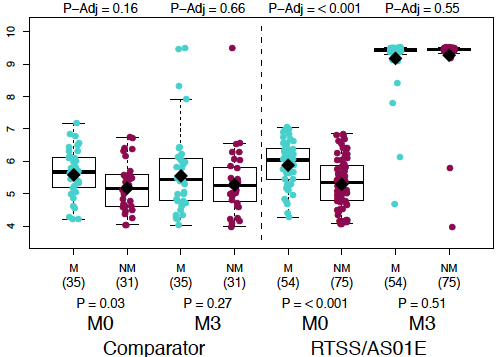

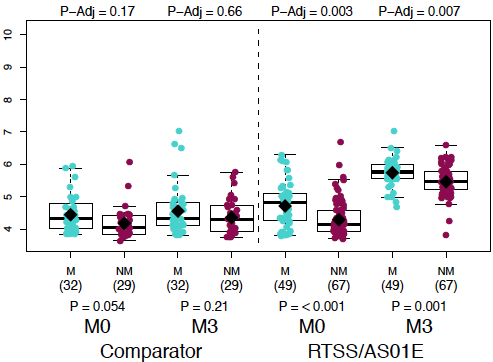
** **
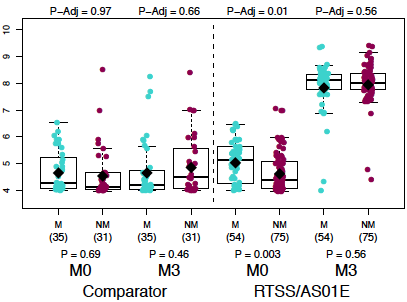
**
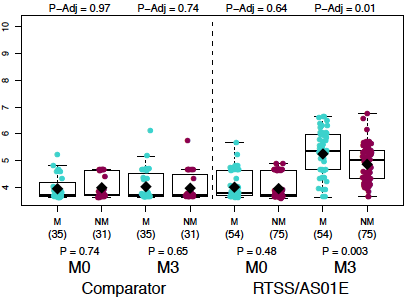


**CSP NANP**

**CSP C-term**

**HBsAg IgG IgG1 IgG2 IgG3 IgG4**

**IgM**

**Figure S11. Levels of antibodies to RTS,S/AS01E antigens by malaria protection status.** Crude levels (log_10_ MFI) of antibodies against CSP and HBsAg in clinical malaria cases (M) and no clinical malaria controls (NM) stratified by vaccine group and timepoint. Groups were compared through t-tests and p-values adjusted by Holm for IgG and IgM and by Benjamini-Hochberg for IgG_1-4_.

**A**

**IgG IgG1 IgG2 IgM**

**B**

**Figure S12. Levels of antibodies to RTS,S/AS01E antigens by clinical malaria protection status stratified by age (A) or by site (B).** Selected antigens shown as examples. Crude levels (log_10_MFI) of antibodies against CSP and HBsAg in clinical malaria cases (M) and no clinical malaria controls (NM) stratified by vaccine group and timepoint. Groups were compared through t-tests and p-values adjusted by Holm for IgG and IgM and by Benjamini-Hochberg for IgG_1-4_. K = Kintampo, M = Manhiça.

**Figure S13. Levels of antibodies to RTS,S/AS01E antigens by clinical malaria protection status stratified by age and site.** Selected antigens shown as examples. Crude levels (log_10_MFI) of antibodies against CSP and HBsAg in clinical malaria cases (M) and no clinical malaria controls (NM) stratified by vaccine group and timepoint. Groups were compared through t-tests and p-values adjusted by Holm for IgG and IgM and by Benjamini-Hochberg for IgG_1-4_. K = Kintampo, M = Manhiça.

**CSP FL IgG1+ IgG3/IgG2+IgG4 IgG (M3-M0**)

**CSP NANP IgG1+ IgG3/IgG2+IgG4 IgG (M3-M0)**

**CSP C-term IgG1+ IgG3/IgG2+IgG4 IgG (M3-M0)**

**Figure S14. Ratios and increments of antibodies to RTS,S/AS01E antigens by clinical malaria protection status stratified by age.** Selected antigens shown as examples. IgG cytophilic/non-cytophilic ratios and increment post-vaccination *vs* pre-vaccination levels. Age group, infants (6-12w) and children (5-17m). M = Clinical malaria cases (non-protected). NM = No clinical malaria (controls). Groups were compared through t-tests and p-values adjusted by Holm.

**CSP FL IgG1+ IgG3/IgG2+IgG4 IgG (M3-M0)**

**CSP NANP IgG1+ IgG3/IgG2+IgG4 IgG (M3-M0)**

**CSP C-term IgG1+ IgG3/IgG2+IgG4 IgG (M3-M0)**

**Figure S15. Ratios and increments of antibodies to RTS,S/AS01E antigens by clinical malaria protection status stratified by site.** Selected antigens shown as examples. IgG cytophilic/non-cytophilic ratios and increment post-vaccination *vs* pre-vaccination levels. M = Clinical malaria cases (non-protected) *vs* NM = No clinical malaria (controls). Groups were compared through t-tests and p-values adjusted by Holm. Site: K = Kintampo; M = Manhiça.

**Figure S16. Ratios and increments of antibodies to RTS,S/AS01E antigens by clinical malaria protection status stratified by age and site.** Selected antigens shown as examples. IgG cytophilic to non-cytophilic ratios and month 0 to month 3 increments stratified by age and site (K = Kintampo; M = Manhiça) at M3. M = Clinical malaria cases (non-protected) *vs* NM = No clinical malaria (controls). Groups were compared through t-tests and p-values adjusted by Holm.

**Table S1.** **Demographic and clinical characteristics of the study population at baseline.** Categorical variables with % subjects in each vaccination category are compared through Chi-square (or Fisher). Continuous variables are summarized through means (and SD) or medians (and IQR) and compared across vaccination groups through t-tests or Wilcoxon rank-sum tests, as appropriate.

|  | RTS,S/AS01E | Comparator | P value |
| --- | --- | --- | --- |
|  | **(N = 129)** | **(N = 66)** |  |
| **Age at baseline, *median (IQR)*, weeks** | 10 (8;46) | 10 (9;43) | 0.65 |
| **Age category, *n (%),* infants (*vs* children)** | 72 (56) | 37 (56) | 1 |
| **Site, *n (%)*** |  |  |  |
| Kintampo | 50 (39) | 28 (42) | 0.64 |
| Manhiça | 79 (61) | 38 (58) |  |
| **Sex, *n (%),* male** | 68 (53) | 29 (44) | 0.29 |
| **Weight, *mean ± SD*, kg** | 6.5 ± 1.96 | 6.6 ± 1.87 | 0.83 |
| **Weight-for-age Z-score, *mean ± SD*** | -0.57 ± 1.06 | -0.45 ± 1.19 | 0.48 |
| **Height, *median (IQR)*, cm** | 58 (55;7) | 59 (55;7)^1^ | 0.80 |
| **Length/height-for-age Z-score, *mean ± SD*** | -1.2 ± 1.07 | -1.02 ± 1.32 | 0.34 |
| **Any vaccination from baseline to M3 other than RTS,S and EPI co-administration in infants, *n (%)*, yes** | 14 (11) | 5 (8) | 0.59 |
| **Vitamin A administration from baseline to M3, *n (%)*, yes** | 19 (15) | 5 (8) | 0.17 |
| **Occurrence of clinical malaria episode from baseline to start of follow-up, *n (%)*, yes** | 12 (9) | 9 (14) | 0.45 |
| **Distance to closest health facility, *median (IQR)*, km** | 2.1 (1.1;9) | 2.15 (1.05;11) | 0.96 |
| **Transmission season when post-vaccination sample was collected, *n (%)*, low (vs high)** | 12 (9) | 9 (14) | 0.47 |
| **Anemia, *n (%)^2^*** |  |  | 0.66 |
| No Anemia | 22 (17) | 15 (23) |  |
| Mild | 51 (40) | 24 (36) |  |
| Moderate or severe | 56 (43) | 27 (41) |  |
| **Baseline hemoglobin level, *mean ± SD*, g/dL** | 9.94 ± 1.39 | 10 ± 1.42 | 0.77 |
| **Parasite density in clinical malaria cases, *Geometric mean (SD)*** | 14532.52 (11.86) | 17989.1 (9.16) | 0.65 |
| **Follow up time to first clinical malaria episode or M12, *median (IQR)*, days** | 365 (176;365) | 320.5 (73.75;365) | 0.01 |

IQR=Interquartile range; SD=standard deviation; kg=kilograms; cm=centimeters.

There are two subjects missing.

^2^ Anemia was defined based on the established cutoff values for baseline hemoglobin: http://www.who.int/vmnis/indicators/haemoglobin.pdf?ua=1

**Table S2. Pairwise correlations (Spearman correlation coefficients)** **(A)** between CSP and HBsAg for each antibody isotype and subclass; **(B)** between isotypes and subclases for each CSP construct and for HBsAg. Some correlations below shall be interpreted with caution because they were estimated based on associations with questionable monotonicity, as illustrated in Fig. S2.

**A**

|  | **CSP NANP** | **CSP C-terminus** | **HBsAg** |
| --- | --- | --- | --- |
| **IgG - CSP** |  |  |  |
| **Full length** | 0.88 | 0.85 | 0.69 |
| **NANP** |  | 0.76 | 0.65 |
| **C-terminus** |  |  | 0.67 |
| **IgM - CSP** |  |  |  |
| **Full length** | 0.85 | 0.79 | 0.37 |
| **NANP** |  | 0.76 | 0.30 |
| **C-terminus** |  |  | 0.32 |
| **IgG1 - CSP** |  |  |  |
| **Full length** | 0.85 | 0.85 | 0.70 |
| **NANP** |  | 0.77 | 0.70 |
| **C-terminus** |  |  | 0.79 |
| **IgG2 - CSP** |  |  |  |
| **Full length** | 0.83 | 0.79 | 0.09 |
| **NANP** |  | 0.74 | 0.08 |
| **C-terminus** |  |  | 0.08 |
| **IgG3 - CSP** |  |  |  |
| **Full length** | 0.78 | 0.80 | 0.44 |
| **NANP** |  | 0.74 | 0.49 |
| **C-terminus** |  |  | 0.43 |
| **IgG4 - CSP** |  |  |  |
| **Full length** | 0.55 | 0.41 | 0.25 |
| **NANP** |  | 0.40 | 0.29 |
| **C-terminus** |  |  | 0.34 |

CSP = circumsporozoite protein; HBsAg = Hepatitis B Surface antigen

**B**

| **Isotypes /Subclasses** | | **Antigen** | | | |
| --- | --- | --- | --- | --- | --- |
|  |  | **CSP-FL** | **CSP C-term** | **CSP NANP** | **HBsAg** |
| **IgG** | **IgG1** | 0.92 | 0.93 | 0.94 | 0.89 |
|  | **IgG2** | 0.81 | 0.78 | 0.74 | 0.32 |
|  | **IgG3** | 0.85 | 0.78 | 0.88 | 0.46 |
|  | **IgG4** | 0.60 | 0.43 | 0.61 | 0.19 |
|  | **IgM** | 0.64 | 0.55 | 0.66 | 0.31 |
| **IgG1** | **IgG** | 0.92 | 0.93 | 0.94 | 0.89 |
|  | **IgG2** | 0.77 | 0.72 | 0.69 | 0.24 |
|  | **IgG3** | 0.81 | 0.74 | 0.83 | 0.44 |
|  | **IgG4** | 0.60 | 0.41 | 0.58 | 0.24 |
|  | **IgM** | 0.61 | 0.52 | 0.65 | 0.30 |
| **IgG2** | **IgG** | 0.81 | 0.78 | 0.74 | 0.32 |
|  | **IgG1** | 0.77 | 0.72 | 0.69 | 0.24 |
|  | **IgG3** | 0.74 | 0.73 | 0.69 | 0.07 |
|  | **IgG4** | 0.54 | 0.43 | 0.49 | 0.12 |
|  | **IgM** | 0.48 | 0.40 | 0.46 | 0.31 |
| **IgG3** | **IgG** | 0.85 | 0.78 | 0.88 | 0.46 |
|  | **IgG1** | 0.81 | 0.74 | 0.83 | 0.44 |
|  | **IgG2** | 0.74 | 0.73 | 0.69 | 0.07 |
|  | **IgG4** | 0.61 | 0.44 | 0.64 | 0.26 |
|  | **IgM** | 0.63 | 0.49 | 0.64 | 0.31 |
| **IgG4** | **IgG** | 0.60 | 0.43 | 0.61 | 0.19 |
|  | **IgG1** | 0.60 | 0.41 | 0.58 | 0.24 |
|  | **IgG2** | 0.54 | 0.43 | 0.49 | 0.12 |
|  | **IgG3** | 0.61 | 0.44 | 0.64 | 0.26 |
|  | **IgM** | 0.64 | 0.55 | 0.66 | 0.10 |
| **IgM** | **IgG** | 0.64 | 0.55 | 0.66 | 0.31 |
|  | **IgG1** | 0.61 | 0.52 | 0.65 | 0.30 |
|  | **IgG3** | 0.63 | 0.49 | 0.64 | 0.31 |
|  | **IgG2** | 0.48 | 0.40 | 0.46 | -0.09 |
|  | **IgG4** | 0.48 | 0.30 | 0.47 | 0.10 |

CSP = circumsporozoite protein; FL= full length; C-term= C-terminus; HBsAg = Hepatitis B Surface antigen.

**Table S3. Effect of RTS,S/AS01E vaccination on antibody responses overall and by age cohort.** Fold-change and 95% confidence interval (CI) in antibody levels (log_10_MFI) from pre-vaccination (M0) to post-vaccination (M3) in RTS,S/AS01E and comparator vaccinees. Estimates were obtained in random intercept models including pre-and post-vaccination antibody levels as outcomes, and indicators for age, post-vaccination visit, and RTS,S/AS01E (*vs* comparator) vaccination. Changes in antibodies from M0 to M3 were significant when 95% CI did not include 1. In models stratified by age group, those estimates in which the interaction of vaccine with post- vs. pre- and age was statistically significant, the adjusted p-value is shown in bold.

|  | **RTS,S/AS01E** | | | | | | | | | | | | | | **Comparator** | | | | | | | | | | | | | | | | | |  |
| --- | --- | --- | --- | --- | --- | --- | --- | --- | --- | --- | --- | --- | --- | --- | --- | --- | --- | --- | --- | --- | --- | --- | --- | --- | --- | --- | --- | --- | --- | --- | --- | --- | --- |
|  | | **Infants** | | | | **Children** | | | | **Overall** | | | | | | | **Infants** | | | | | **Children** | | | | | **Overall** | | | | | |  |
| **Antibody** | **Change M0-M3** | | **95% CI** | | **Change M0-M3** | | **95% CI** | P value^†^ | | | **Change M0-M3** | | | **95% CI** | | **Change M0-M3** | | | **95% CI** | | **Change M0-M3** | | | **95% CI** | | P value | | | **Change M0-M3** | | **95% CI** | | |
| IgG |  | |  | |  | |  | |  | | |  |  | | |  | |  | |  | | |  | |  | | | |  | |  | |  |
| FL | 1433 | | 860; 2388 | | 4123 | | 2323; 7319 | | **0.04** | | | 2286 | 1548; 3375 | | | 2 | | 0.8; 3 | | 0.9 | | | 0.4; 2 | | 0.49 | | | | 1.3 | | 0.8 ; 2.2 | |  |
| C-term | 1224 | | 804; 1862 | | 2516 | | 1569; 4034 | | 0.13 | | | 1683 | 1220; 2320 | | | 2 | | 1; 4 | | 0.9 | | | 0.5; 2 | | 0.14 | | | | 1.6 | | 1 ; 2.4 | |  |
| NANP | 673 | | 393; 1152 | | 1728 | | 944; 3162 | | 0.13 | | | 1020 | 674; 1545 | | | 4 | | 2; 9 | | 0.9 | | | 0.4; 2 | | 0.04 | | | | 2.1 | | 1.2 ; 3.8 | |  |
| IgM |  | |  | |  | |  | |  | | |  |  | | |  | |  | |  | | |  | |  | | | |  | |  | |  |
| FL | 17 | | 12; 24 | | 15 | | 10; 23 | | 0.74 | | | 16 | 12; 21 | | | 2 | | 1; 3 | | 0.8 | | | 0.4; 1 | | 0.26 | | | | 1.2 | | 0.8 ; 1.7 | |  |
| C-term | 5 | | 4; 8 | | 4 | | 3; 5 | | 0.46 | | | 5 | 3.5; 6 | | | 2 | | 1; 3 | | 1 | | | 0.6; 2 | | 0.63 | | | | 1.3 | | 0.9 ; 1.9 | |  |
| NANP | 11 | | 8; 17 | | 19 | | 12; 30 | | 0.46 | | | 14 | 10.5; 19 | | | 2 | | 1; 3 | | 0.9 | | | 0.5; 2 | | 0.46 | | | | 1.4 | | 0.9 ; 2.1 | |  |
| IgG1 |  | |  | |  | |  | |  | | |  |  | | |  | |  | |  | | |  | |  | | | |  | |  | |  |
| FL | 3303 | | 1800; 6061 | | 8366 | | 4229; 16549 | | 0.09 | | | 4980 | 3147; 7882 | | | 2 | | 0.7; 4 | | 0.9 | | | 0.3; 2 | | 0.52 | | | | 1.2 | | 0.6 ; 2.3 | |  |
| C-term | 2538 | | 1556; 4140 | | 4867 | | 2808; 8436 | | 0.16 | | | 3384 | 2330; 4915 | | | 3 | | 2; 6 | | 0.9 | | | 0.4; 2 | | 0.04 | | | | 1.8 | | 1.1 ; 3.1 | |  |
| NANP | 1479 | | 766; 2855 | | 3843 | | 1835; 8048 | | 0.13 | | | 2256 | 1361; 3738 | | | 6 | | 2; 14 | | 0.8 | | | 0.3; 2 | | 0.02 | | | | 2.4 | | 1.2 ; 4.8 | |  |
| IgG2 |  | |  | |  | |  | |  | | |  |  | | |  | |  | |  | | |  | |  | | | |  | |  | |  |
| FL | 8 | | 5; 12 | | 23 | | 14; 35 | | **0.003** | | | 12 | 9; 17 | | | 2 | | 1; 3 | | 1 | | | 0.6; 2 | | 0.52 | | | | 1.4 | | 1 ; 2.2 | |  |
| C-term | 19 | | 13; 27 | | 16 | | 11; 25 | | 0.75 | | | 18 | 13; 23 | | | 2 | | 1; 3 | | 1 | | | 0.6; 2 | | 0.25 | | | | 1.4 | | 0.9 ; 2 | |  |
| NANP | 6 | | 4; 8 | | 24 | | 16; 36 | | **<0.001** | | | 10 | 8; 14 | | | 1 | | 0.7; 2 | | 1 | | | 0.6; 2 | | 0.75 | | | | 1.1 | | 0.7 ; 1.6 | |  |
| IgG3 |  | |  | |  | |  | |  | | |  |  | | |  | |  | |  | | |  | |  | | | |  | |  | |  |
| FL | 723 | | 378; 1380 | | 2669 | | 1290; 5524 | | **0.02** | | | 1287 | 782; 2117 | | | 3 | | 1; 7 | | 0.7 | | | 0.2; 2 | | 0.15 | | | | 1.5 | | 0.8 ; 3.1 | |  |
| C-term | 142 | | 87; 234 | | 338 | | 193.7; 590 | | **0.04** | | | 209 | 142; 305 | | | 2 | | 0.8; 3 | | 0.4 | | | 0.2; 0.9 | | 0.04 | | | | 0.9 | | 0.5 ; 1.5 | |  |
| NANP | 273 | | 146; 508 | | 743 | | 369; 1496 | | 0.09 | | | 425 | 262; 688 | | | 5 | | 2; 11 | | 0.6 | | | 0.2; 2 | | 0.01 | | | | 1.9 | | 1 ; 3.7 | |  |
| IgG4 |  | |  | |  | |  | |  | | |  |  | | |  | |  | |  | | |  | |  | | | |  | |  | |  |
| FL | 10 | | 6; 14 | | 16 | | 10; 25 | | 0.21 | | | 12 | 9; 16 | | | 1 | | 0.6; 2 | | 1 | | | 0.6; 2 | | 0.98 | | | | 1.1 | | 0.7 ; 1.7 | |  |
| C-term | 3 | | 2; 5 | | 6 | | 3.9; 10 | | 0.15 | | | 4 | 3; 6 | | | 1 | | 0.8; 2 | | 0.8 | | | 0.4; 2 | | 0.23 | | | | 1.1 | | 0.7 ; 1.7 | |  |
| NANP | 7 | | 5; 10 | | 8 | | 4.9; 12 | | 0.88 | | | 7 | 6; 10 | | | 2 | | 0.9; 2.7 | | 0.6 | | | 0.4; 1 | | 0.03 | | | | 1.1 | | 0.7 ; 1.6 | |  |
| HBsAg |  | | | | | | | | | | | | | | | | | | | | | | | | | | |  | |  | |  | |
| IgG | 8 | | 5; 11 | 100 | | | 66.7; 151 | | **< 0.001** | | | 24 | 17; 33 | | | 2 | | 1; 2.7 | | 1 | | | 0.6; 2 | | 0.49 | | | | 1.4 | | 0.9 ; 2.2 | |  |
| IgM | 9 | | 6; 13 | 96 | | | 60.1; 153 | | **0.02** | | | 25 | 18; 36 | | | 1 | | 0.8; 2 | | 1 | | | 0.6; 2 | | 0.07 | | | | 1.9 | | 1.4 ; 2.5 | |  |
| IgG1 | 0.4 | | 0.3; 0.5 | 2 | | | 1; 2 | | **<0.001** | | | 0.7 | 0.6; 0.9 | | | 0.3 | | 0.2; 0.5 | | 1 | | | 0.9; 2 | | 0.80 | | | | 1.3 | | 0.8 ; 2.1 | |  |
| IgG2 | 2 | | 1; 3 | 4 | | | 2; 7 | | **<0.001** | | | 2 | 2; 4 | | | 0.9 | | 0.4; 2 | | 1 | | | 0.6; 3 | | <0.001 | | | | 0.6 | | 0.5 ; 0.8 | |  |
| IgG3 | 1 | | 1; 1 | 1 | | | 1; 1 | | **0.04** | | | 1 | 1.1; 1.2 | | | 1 | | 0.9; 1.1 | | 1 | | | 0.9; 1.1 | | 0.75 | | | | 1.1 | | 0.6 ; 1.9 | |  |
| IgG4 | 5 | | 3; 6 | 2 | | | 2; 3 | | **0.006** | | | 3 | 2.6; 4 | | | 2 | | 1.6; 34 | | 1 | | | 0.9; 2 | | 0.75 | | | | 1 | | 0.9 ; 1.1 | |  |

CSP = circumsporozoite protein; FL = full length; HBsAg = Hepatitis B surface antigen.

^†^P values from the Wald test of the interaction age:visit in stratified models; the predictors were: agec_num + visit_num + visit_num:agec_num. Differences between infants and children in the effect of vaccination on antibody responses were statistically significant (unadjusted p-value of interaction with age group =0.006 to <0.001) for all antigens/isotypes/subclasses, except for CSP C-term IgG2, CSP FL IgG4, and CSP FL and C-term IgM (p>0.17) that had non-significant unadjusted p-values, and for CSP FL IgG and IgG1, CSP NANP and C-term IgG4, and CSP NANP IgM (p=0.02 to 0.06) that had borderline unadjusted p-values. In the case of HBsAg, differences were only statistically significant for IgG and IgG1 (p<0.001) and borderline significant for IgG4 (p=0.04).

**Table S4. Effect of age as continuous variable on RTS,S/AS01E immunogenicity**. A linear model was fitted within RTS,S vaccinees at month 3 (M3) and the main effect of the covariate assessed with Coef (coefficient) and CI (confidence interval), adjusted by site. Each cohort is shown separately in each row: infants (n=72)/ children (n=57). The interpretation of the outcome variable is in the MFI scale (percent change for a unit change in the predictor [weeks]). Sample size for IgG2 was smaller (N=116 *vs* N=129 for the rest). Multiple testing adjustments (P-Adj) took into account the two broken stick model partitions as well as the three strata of the data (RTS,S M3, Comparators M3, all M0).

| **Isotype** | **Antigen** | **Coef(CI)** | **P** | **P-Adj** |
| --- | --- | --- | --- | --- |
| IgG | CSP FL | 67.7(-36.9;345.67)/ 118.48(-8.8;423.41) | 0.30/  0.08 | 0.40/  0.15 |
|  | CSP C-term | 128.68(-3.64;442.72)/ 104.98(-5.33;343.84) | 0.06/  0.07 | 0.15/  0.15 |
|  | CSP NANP | 167.33(-2.35;631.87)/ 198.16(21.19;633.53) | 0.056/  **0.02** | 0.15/  0.053 |
|  | HBsAg | 905.4(332.96;2234.68)/ 962.31(400.26;2155.86) | **< 0.001/**  **< 0.001** | **< 0.001/**  **< 0.001** |
| IgG1 | CSP FL | 17.66(-61.16;256.44)/ 47.71(-45.15;297.8) | 0.77/  0.44 | 0.83/  0.61 |
|  | CSP C-term | 250.17(19.24;928.39)/  134.5(-10.48;514.29) | 0.02/  0.08 | 0.11/  0.26 |
|  | CSP NANP | 137.24(-28.36;685.67)/ 150.98(-13.94;631.98) | 0.16/  0.09 | 0.31/  0.27 |
|  | HBsAg | 1148.16(424.93;2867.81)/ 1203.56(501.02;2727.35) | **< 0.001/**  **< 0.001** | **< 0.001/**  **< 0.001** |
| IgG2 | CSP FL | -25.5(-59.08;35.64)/  3.38(-41.23;81.87) | 0.33/  0.91 | 0.51/  0.93 |
|  | CSP C-term | -54.14(-79.67;3.42)/  -21.37(-63.47;69.25) | 0.06/  0.54 | 0.21/  0.66 |
|  | CSP NANP | 29.87(-35.16;160.12)/ 66.75(-13.36;220.94) | 0.46/  0.12 | 0.61/  0.29 |
|  | HBsAg | -45.93(-74.91;16.53)/ 195.87(43.46;510.2) | 0.12/  **0.004** | 0.28/  **0.04** |
| IgG3 | CSP FL | 102.41(-35.99;540.06)/ 65.93(-40.71;364.35) | 0.23/  0.33 | 0.40/  0.51 |
|  | CSP C-term | 145.7(-3.08;522.85)/ 134.23(1.99;437.97) | 0.058/  **0.045** | 0.21/  0.19 |
|  | CSP NANP | 374.54(45.36;1449.17)/  109.06(-27.39;501.96) | **0.01**/  0.17 | 0.06/  0.33 |
|  | HBsAg | 238.86(90.3;503.38)/  233.7(99.24;458.9) | **< 0.001/**  **< 0.001** | **< 0.001/**  **< 0.001** |
| IgG4 | CSP FL | 45.22(-45.34;285.79)/  91.25(-20.15;358.04) | 0.45/  0.14 | 0.61/  0.30 |
|  | CSP C-term | 25.92(-56.19;261.92)/  103(-21;421.61) | 0.67/  0.14 | 0.76/  0.30 |
|  | CSP NANP | 42.34(-42.28;250.99)/  30.64(-41.7;192.73) | 0.44/  0.51 | 0.61/  0.64 |
|  | HBsAg | 22.93(2.2;47.87)/  42.95(21.19;68.6) | **0.03/**  **< 0.001** | 0.13/  **< 0.001** |
| IgM | CSP FL | 58.98(-28.2;252.02)/  57.11(-22.8;219.73) | 0.25/  0.21 | 0.35/  0.34 |
|  | CSP C-term | -20.05(-57.27;49.59)/  -12.3(-49.91;53.55) | 0.48/  0.64 | 0.58/  0.67 |
|  | CSP NANP | 67.46(-26.44;281.22)/  96.26(-5.92;309.42) | 0.22/  0.07 | 0.34/  0.22 |
|  | HBsAg | 545.71(225.92;1179.29)/ 1173.88(591.37;2247.17) | **< 0.001/**  **< 0.001** | **< 0.001/**  **< 0.001** |

**Table S5. Percentage seropositivity after vaccination.**

|  | | **All** | | | **Infants** | | | **Children** | | |
| --- | --- | --- | --- | --- | --- | --- | --- | --- | --- | --- |
|  | | **RTSS/AS01E** | **Comparator** | **Chi** | **RTSS/AS01E** | **Comparator** | **Chi** | **RTSS/AS01E** | **Comparator** | **Chi** |
| **Isotype** | **Antigen** | **N/Total (%)** | **N/Total (%)** |  | **N/Total (%)** | **N/Total (%)** |  | **N/Total (%)** | **N/Total (%)** |  |
| IgG | CSP_FL | 125/129 (97) | 7/66 (11) | **<0.001** | 69/72 (96) | 7/37 (19) | **<0.001** | 56/57 (98) | 0/29 (0) | **<0.001** |
|  | CSP_Cterm | 126/129 (98) | 6/66 (9) | **<0.001** | 70/72 (97) | 6/37 (16) | **<0.001** | 56/57 (98) | 0/29 (0) | **<0.001** |
|  | CSP_NANP | 125/129 (97) | 23/66 (35) | **<0.001** | 69/72 (96) | 18/37 (49) | **<0.001** | 56/57 (98) | 5/29 (17) | **<0.001** |
|  | HBsAg | 85/129 (66) | 6/66 (9) | **<0.001** | 31/72 (43) | 3/37 (8) | **<0.001** | 54/57 (95) | 3/29 (10) | **<0.001** |
| IgG1 | CSP_FL | 125/129 (97) | 9/66 (14) | **<0.001** | 69/72 (96) | 7/37 (19) | **<0.001** | 56/57 (98) | 2/29 (7) | **<0.001** |
|  | CSP_Cterm | 125/129 (97) | 8/66 (12) | **<0.001** | 69/72 (96) | 8/37 (22) | **<0.001** | 56/57 (98) | 0/29 (0) | **<0.001** |
|  | CSP_NANP | 125/129 (97) | 27/66 (41) | **<0.001** | 69/72 (96) | 20/37 (54) | **<0.001** | 56/57 (98) | 7/29 (24) | **<0.001** |
|  | HBsAg | 67/129 (52) | 4/66 (6) | **<0.001** | 18/72 (25) | 2/37 (5) | **0.02** | 49/57 (86) | 2/29 (7) | **<0.001** |
| IgG2 | CSP_FL | 40/116 (34) | 3/61 (5) | **<0.001** | 24/67 (36) | 3/33 (9) | **0.007** | 16/49 (33) | 0/28 (0) | **0.002** |
|  | CSP_Cterm | 51/116 (44) | 4/61 (7) | **<0.001** | 32/67 (48) | 4/33 (12) | **<0.001** | 19/49 (39) | 0/28 (0) | **0.001** |
|  | CSP_NANP | 36/116 (31) | 2/61 (3) | **<0.001** | 17/67 (25) | 2/33 (6) | **0.03** | 19/49 (39) | 0/28 (0) | **<0.001** |
|  | HBsAg | 1/116 (1) | 1/61 (2) | 1 | 0/67 (0) | 0/33 (0) | 1 | 1/49 (2) | 1/28 (4) | 1 |
| IgG3 | CSP_FL | 125/129 (97) | 16/66 (24) | **<0.001** | 69/72 (96) | 11/37 (30) | **<0.001** | 56/57 (98) | 5/29 (17) | **<0.001** |
|  | CSP_Cterm | 126/129 (98) | 9/66 (14) | **<0.001** | 70/72 (97) | 7/37 (19) | **<0.001** | 56/57 (98) | 2/29 (7) | **<0.001** |
|  | CSP_NANP | 126/129 (98) | 19/66 (29) | **<0.001** | 70/72 (97) | 14/37 (38) | **<0.001** | 56/57 (98) | 5/29 (17) | **<0.001** |
|  | HBsAg | 9/129 (7) | 8/66 (12) | 0.28 | 2/72 (3) | 3/37 (8) | 0.34 | 7/57 (12) | 5/29 (17) | 0.75 |
| IgG4 | CSP_FL | 124/129 (96) | 22/66 (33) | **<0.001** | 68/72 (94) | 12/37 (32) | **<0.001** | 56/57 (98) | 10/29 (34) | **<0.001** |
|  | CSP_Cterm | 34/129 (26) | 1/66 (2) | **< 0.001** | 13/72 (18) | 1/37 (3) | **0.03** | 21/57 (37) | 0/29 (0) | **<0.001** |
|  | CSP_NANP | 110/129 (85) | 21/66 (32) | **< 0.001** | 60/72 (83) | 16/37 (43) | **< 0.001** | 50/57 (88) | 5/29 (17) | **<0.001** |
|  | HBsAg | 0/129 (0) | 0/66 (0) | 1 | 0/72 (0) | 0/37 (0) | 1 | 0/57 (0) | 0/29 (0) | 1 |
| IgM | CSP_FL | 122/129 (95) | 30/66 (45) | **< 0.001** | 69/72 (96) | 15/37 (41) | **< 0.001** | 53/57 (93) | 15/29 (52) | **< 0.001** |
|  | CSP_Cterm | 107/129 (83) | 21/66 (32) | **< 0.001** | 61/72 (85) | 11/37 (30) | **< 0.001** | 46/57 (81) | 10/29 (34) | **< 0.001** |
|  | CSP_NANP | 121/129 (94) | 30/66 (45) | **< 0.001** | 69/72 (96) | 19/37 (51) | **< 0.001** | 52/57 (91) | 11/29 (38) | **< 0.001** |
|  | HBsAg | 15/129 (12) | 3/66 (5) | 0.11 | 2/72 (3) | 0/37 (0) | 0.55 | 13/57 (23) | 3/29 (10) | 0.24 |

**Table S6. Effect of site on RTS,S/AS01E immunogenicity**. A linear model was fitted within RTS,S vaccinees at month 3 (M3) and the main effect of the covariate assessed with Coef (coefficient) and CI (confidence interval). The interpretation of the outcome variable is in the MFI scale (percent change Manhiça over Kintampo: effect of being a subject from Manhiça taking Kintampo as the baseline or reference site). Sample size for IgG2 was smaller (N=116 *vs* N=129 for the rest). Multiple testing adjustments (P-Adj) took into account the three strata of the data (RTS,S M3, Comparators M3, all M0).

|  |  | **All (N=129)** | | | **Infants (N=72)** | | | **Children (N= 57)** | | |
| --- | --- | --- | --- | --- | --- | --- | --- | --- | --- | --- |
| **Isotype** | **Antigen** | **Coef(CI)** | **P** | **P-Adj** | **Coef(CI)** | **P** | **P-Adj** | **Coef(CI)** | **P** | **P-Adj** |
| IgG | CSP FL | 15.14 (-35.4;105.23) | 0.63 | 1 | -11.63 (-62.35;107.43) | 0.77 | 1 | 70.66 (-18.96;259.4) | 0.16 | 1 |
|  | CSP C-term | 2.14 (-39.07;71.23) | 0.94 | 1 | -20.35 (-60.63;61.16) | 0.52 | 1 | 50.3 (-28.1;214.2) | 0.27 | 1 |
|  | CSP NANP | 35.07 (-26.46;148.1) | 0.33 | 1 | 43.77 (-38.5;236.12) | 0.40 | 1 | 41.34 (-38.54;225.01) | 0.41 | 1 |
|  | HBsAg | 23.23 (-33.2;127.34) | 0.50 | 1 | 92.59 (0.85;267.77) | **0.047** | 0.42 | -4.74 (-56.49;108.59) | 0.90 | 1 |
| IgG1 | CSP FL | -2.27 (-48.78;86.47) | 0.94 | 0.97 | -37.8 (-76.47;64.43) | 0.33 | 0.62 | 73.43 (-23.72;294.33) | 0.18 | 0.42 |
|  | CSP C-term | 27.64 (-33.2;143.91) | 0.46 | 0.69 | 5.87 (-57.6;164.39) | 0.90 | 0.97 | 79.66 (-25.12;331.08) | 0.19 | 0.42 |
|  | CSP NANP | 16.44 (-42.76;136.87) | 0.67 | 0.87 | -8.57 (-68.4;164.49) | 0.87 | 0.97 | 72.24 (-29.14;318.7) | 0.23 | 0.46 |
|  | HBsAg | 28.86 (-32.28;145.23) | 0.44 | 0.69 | 89.73 (0.84;256.99) | **0.047** | 0.15 | 9.51 (-53.51;158) | 0.83 | 0.97 |
| IgG2 | CSP FL | -65.71 (-75.82;-51.38) | **<0.001** | **<0.001** | -66.3 (-78.42;-47.38) | **<0.001** | **<0.001** | -65.85 (-80.99;-38.66) | **<0.001** | **0.006** |
|  | CSP C-term | -74.77 (-84.4;-59.2) | **<0.001** | **<0.001** | -73.82 (-86.91;-47.66) | **<0.001** | **0.002** | -78.26 (-88.67;-58.3) | **<0.001** | **<0.001** |
|  | CSP NANP | -54.18 (-69.55;-31.05) | **<0.001** | **0.001** | -67.34 (-82.09;-40.47) | **< 0.001** | **0.003** | -23.19 (-53.47;26.8) | 0.30 | 0.54 |
|  | HBsAg | 57.51 (-1.21;151.16) | 0.056 | 0.15 | 19.77 (-31.27;108.72) | 0.52 | 0.80 | 137.24 (5.07;435.7) | **0.04** | 0.12 |
| IgG3 | CSP FL | -20.27 (-59.44;56.73) | 0.51 | 0.74 | -29 (-73.59;90.86) | 0.49 | 0.79 | -1.78 (-60.6;144.82) | 0.97 | 0.99 |
|  | CSP C-term | 10.41 (-36.8;92.86) | 0.73 | 0.89 | 36.28 (-36.99;194.78) | 0.43 | 0.73 | -4.87 (-56.44;107.78) | 0.90 | 0.98 |
|  | CSP NANP | -37.9 (-69.53;26.56) | 0.19 | 0.42 | 6.02 (-61.57;192.51) | 0.91 | 0.97 | -62.7 (-85.32;-5.22) | **0.04** | 0.12 |
|  | HBsAg | 16.78 (-20.59;71.75) | 0.43 | 0.69 | 42.39 (-3.12;109.29) | 0.07 | 0.21 | 6.57 (-41.35;93.64) | 0.83 | 0.97 |
| IgG4 | CSP FL | -73.94 (-85.33;-53.72) | **<0.001** | **<0.001** | -72.69 (-86.8;-43.49) | **< 0.001** | **0.004** | -73.86 (-89.76;-33.29) | **0.006** | **0.04** |
|  | CSP C-term | 13.29 (-39.04;110.52) | 0.69 | 0.87 | 47.47 (-24.4;187.66) | 0.25 | 0.56 | -12.3 (-71.66;171.39) | 0.82 | 0.97 |
|  | CSP NANP | -24.86 (-55.63;27.26) | 0.29 | 0.53 | -9.65 (-55.46;83.29) | 0.78 | 0.97 | -37.73 (-72.45;40.76) | 0.25 | 0.48 |
|  | HBsAg | 12.95 (0.46;26.99) | **0.042** | 0.13 | 5.62 (-4.46;16.76) | 0.28 | 0.56 | 26.77 (2.84;56.28) | **0.03** | 0.1 |
| IgM | CSP FL | 11.94 (-29.87;78.68) | 0.63 | 1 | -22.65 (-54.7;32.09) | 0.34 | 1 | 84.43 (-17.23;310.94) | 0.13 | 0.79 |
|  | CSP C-term | 16.56 (-19.11;67.94) | 0.41 | 1 | 12.42 (-31.17;83.6) | 0.64 | 1 | 18.95 (-32.55;109.78) | 0.54 | 1 |
|  | CSP NANP | 9.73 (-32.61;78.69) | 0.71 | 1 | -4.88 (-46.03;67.64) | 0.86 | 1 | 40.16 (-39.03;222.23) | 0.42 | 1 |
|  | HBsAg | 104.2 (19.76;248.18) | **0.009** | **0.11** | 196.08 (72.01;409.65) | **< 0.001** | **0.002** | 71.19 (-7.73;217.61) | 0.09 | 0.70 |

**Table S7. Effect of site on baseline antibodies**. A linear model was fitted at pre-vaccination (M0) and the main effect of the covariates assessed with Coef (coefficient) and 95% confidence interval (CI). Manhiça reference Kintampo. The interpretation of the outcome variable is in the MFI scale (percent change for a unit change in the predictor). Sample size for IgG2 was smaller (N=116 *vs* N=129 for the rest). Multiple testing adjustments (P-Adj) took into account three data strata (RTS,S M3, Comparators M3, all M0).

|  |  | **All (N=195)** | | | **Infants (N=109)** | | | **Children (N= 86)** | | |
| --- | --- | --- | --- | --- | --- | --- | --- | --- | --- | --- |
| **Isotype** | **Antigen** | **Coef(CI)** | **P** | **P-Adj** | **Coef(CI)** | **P** | **P-Adj** | **Coef(CI)** | **P** | **P-Adj** |
| IgG | CSP FL | -74.62 (-81.63;-64.92) | **<0.001** | **<0.001** | -71.19 (-79.43;-59.64) | **<0.001** | **< 0.001** | -80.45 (-88.87;-65.67) | **<0.001** | **<0.001** |
|  | CSP C-term | -52.27 (-63.36;-37.82) | **<0.001** | **<0.001** | -41.3 (-59.9;-14.07) | **0.007** | 0.07 | -63.23 (-74.43;-47.12) | **<0.001** | **<0.001** |
|  | CSP NANP | -60.38 (-72.48;-42.95) | **<0.001** | **<0.001** | -72.86 (-82.83;-57.1) | **<0.001** | **< 0.001** | -40.05 (-66.61;7.63) | 0.09 | 0.70 |
|  | HBsAg | -0.74 (-29.35;39.47) | 0.60 | 1 | 21.16 (-22.49;89.4) | 0.40 | 1 | -26.51 (-56.28;23.53) | 0.24 | 1 |
| IgG1 | CSP FL | -84.07 (-89.89;-74.9) | **<0.001** | **<0.001** | -79.09 (-87.96;-63.69) | **<0.001** | **< 0.001** | -89.9 (-95.1;-79.21) | **<0.001** | **<0.001** |
|  | CSP C-term | -49.49 (-65.38;-26.3) | **0.03** | 0.1 | -46.28 (-69.04;-6.79) | **0.03** | 0.1 | -51.64 (-71.17;-18.9) | **0.006** | **0.04** |
|  | CSP NANP | -70.32 (-81.68;-51.92) | **<0.001** | **<0.001** | -75.94 (-87.06;-55.25) | **<0.001** | **< 0.001** | -64.25 (-83.42;-22.92) | **0.009** | **0.047** |
|  | HBsAg | -7.39 (-37.5;37.23) | 0.86 | 0.95 | 2.56 (-38.66;71.48) | 0.92 | 0.97 | -20.66 (-57.4;47.79) | 0.46 | 0.67 |
| IgG2 | CSP FL | -67.07 (-78.11;-50.46) | **<0.001** | **<0.001** | -75.36 (-86.56;-54.84) | **<0.001** | **< 0.001** | -63.83 (-76.01;-45.46) | **<0.001** | **<0.001** |
|  | CSP C-term | -27.85 (-43.79;-7.37) | **0.004** | **0.02** | -35.82 (-55.88;-6.62) | **0.02** | 0.08 | -22.06 (-43.07;6.71) | 0.12 | 0.30 |
|  | CSP NANP | -64.79 (-75.95;-48.46) | **<0.001** | **<0.001** | -82.05 (-89.58;-69.07) | **<0.001** | **< 0.001** | -37.6 (-57.07;-9.3) | **0.01** | 0.06 |
|  | HBsAg | 121.62 (41.7;246.62) | **0.006** | **0.02** | 99.93 (12.02;256.84) | **0.02** | 0.08 | 88.99 (2.62;248.04) | **0.041** | 0.12 |
| IgG3 | CSP FL | -60.75 (-77;-33.04) | **<0.001** | **0.001** | -56.17 (-77.84;-13.33) | **0.02** | 0.08 | -68.06 (-86.46;-24.66) | **0.01** | **0.047** |
|  | CSP C-term | -10.5 (-41.78;37.57) | 0.46 | 0.69 | -0.97 (-43.26;72.82) | 0.97 | 0.97 | -18.37 (-59;62.51) | 0.56 | 0.75 |
|  | CSP NANP | -47.42 (-69.17;-10.32) | **0.008** | **0.03** | -70.09 (-85.18;-39.65) | **<0.001** | **0.005** | 5.85 (-53.35;140.2) | 0.89 | 0.98 |
|  | HBsAg | -12.13 (-46.52;44.39) | 0.54 | 0.75 | -10.92 (-52.32;66.43) | 0.71 | 0.96 | -10.61 (-60.5;102.5) | 0.79 | 0.97 |
| IgG4 | CSP FL | -0.98 (-26.81;33.96) | 0.89 | 0.95 | -4.98 (-37.55;44.58) | 0.81 | 0.97 | 3.44 (-33.93;61.95) | 0.88 | 0.98 |
|  | CSP C-term | -26.16 (-43.35;-3.77) | 0.34 | 0.59 | -20.19 (-46.01;17.97) | 0.26 | 0.56 | -33.66 (-53.51;-5.34) | **0.02** | 0.1 |
|  | CSP NANP | -30.19 (-47.92;-6.43) | **0.03** | 0.1 | -27.81 (-51.7;7.9) | 0.11 | 0.31 | -31.24 (-55.68;6.66) | 0.09 | 0.26 |
|  | HBsAg | 8.58 (1.7;15.93) | 0.17 | 0.42 | -2.62 (-10.49;5.94) | 0.53 | 0.80 | 24.62 (13.07;37.34) | **<0.001** | **<0.001** |
| IgM | CSP FL | -19.82 (-42.31;11.45) | 0.08 | 0.53 | 39.94 (-7.02;110.64) | 0.11 | 0.75 | -55.28 (-72.74;-26.64) | **0.002** | **0.02** |
|  | CSP C-term | -25.58 (-45.78;2.16) | **0.02** | 0.15 | 42.95 (-5.99;117.34) | 0.09 | 0.75 | -64.51 (-77.13;-44.92) | **<0.001** | **<0.001** |
|  | CSP NANP | -25.69 (-48;6.18) | **0.01** | 0.13 | 25.43 (-22.99;104.29) | 0.36 | 1 | -60.22 (-76.07;-33.85) | **<0.001** | **0.006** |
|  | HBsAg | 1.62 (-37.5;65.22) | 0.63 | 1 | 19.08 (-14.56;65.98) | 0.30 | 1 | 34.16 (-17.41;117.96) | 0.23 | 1 |

**Table S8**. **Antigen targets for maternal antibodies and malaria exposure indices.** Summary score variables for the constructs “maternal antibodies” and “exposure to *Plasmodium falciparum*” obtained in a principal component analysis (PCA), and correlation with baseline CSP IgG responses. See also Fig. S10. **A)** Loadings of “maternal IgG antibodies” at baseline in the subset of subjects <10 months (43.45 weeks) at the date of dose 1 (n= 145 subjects < 10 months at the date of the dose 1, 145/195 = 74.35%). **B)** Loadings of “*P. falciparum* IgM exposure antibodies” at baseline. Numbers correspond to the relative contribution of each antigen (loadings) to components that explained jointly up to 70% of the data variability. The first component of each PCA (PC1) was used for analysis. **C)** Correlations between baseline RTS,S IgG responses and maternal antibodies index (PC1) in age < 10 months. **D)** Correlations between baseline RTS,S IgG responses and malaria exposure index (PC1). Spearman correlation coefficient (rho), with confidence interval at 95%, and p value of the t-test of the individual correlation. Adjusted p values for exposure index by Holm and for maternal index by Benjamini-Hochberg.

**A**

|  | **PC1** | **PC2** | **PC3** |
| --- | --- | --- | --- |
| AMA-1 3D7 | 0.24 | 0.06 | -0.57 |
| AMA-1 FVO | 0.24 | 0.07 | -0.55 |
| DBL1 DBL2 3D7 | 0.21 | 0.53 | 0.19 |
| DBL3 DBL4 FCR3 | 0.21 | 0.54 | 0.14 |
| EBA-175 R2 F2 | 0.24 | -0.24 | -0.08 |
| EBA-175 R3-5 | 0.26 | -0.16 | 0.05 |
| EXP-1 | 0.28 | 0.07 | 0.18 |
| LSA-1 | 0.20 | -0.15 | 0.36 |
| MSP-1_42_ 3D7 | 0.27 | -0.20 | -0.01 |
| MSP-1_42_ FVO | 0.24 | -0.38 | 0.02 |
| MSP-1 block 2 HYBRID | 0.24 | -0.22 | 0.12 |
| MSP-2 full length CH150 | 0.28 | 0.07 | 0.16 |
| MSP-2 full length DD2 | 0.28 | 0.13 | 0.04 |
| MSP-3 3D7 | 0.22 | 0.19 | -0.06 |
| MSP-6 | 0.24 | -0.17 | 0.28 |
| RH2 (2030) | 0.25 | -0.02 | -0.15 |
| SSP2 / TRAP | 0.18 | -0.01 | -0.11 |
| Cumulative Proportion | 0.54 | 0.63 | 0.70 |

**B**

|  | **PC1** | **PC2** | **PC3** |
| --- | --- | --- | --- |
| CELTOS | 0.19 | -0.07 | 0.06 |
| CYRPA full length | 0.20 | 0.31 | -0.16 |
| EBA140 R3-5 | 0.19 | 0.01 | 0.21 |
| EBA175 R2 F2 | 0.20 | 0.17 | -0.12 |
| EBA175 R3-5 | 0.20 | 0.18 | 0.09 |
| EXP-1 | 0.18 | -0.15 | -0.19 |
| LSA-1 | 0.17 | -0.00 | 0.03 |
| MSP-1_42_ 3D7 | 0.19 | 0.10 | -0.32 |
| MSP-1_42_ FVO | 0.20 | 0.03 | -0.33 |
| MSP-1 block 2 3D7 | 0.19 | -0.19 | -0.18 |
| MSP-1 block 2 hybrid | 0.20 | -0.32 | -0.14 |
| MSP-1 block 2 MAD20 | 0.18 | -0.42 | -0.01 |
| MSP-1 block 2 PA17 | 0.17 | -0.20 | -0.36 |
| MSP-1 block 2 R033 | 0.17 | -0.04 | 0.05 |
| MSP-1 block 2 Wellcome | 0.20 | -0.32 | 0.14 |
| MSP-2 full length CH150 | 0.19 | -0.13 | -0.01 |
| MSP-2 full length DD2 | 0.19 | -0.28 | 0.22 |
| MSP-3 3C | 0.17 | 0.20 | 0.28 |
| MSP-3 3D7 | 0.18 | 0.28 | -0.12 |
| MSP-5 | 0.18 | -0.03 | 0.45 |
| MSP-6 | 0.20 | 0.11 | 0.16 |
| P41 | 0.21 | 0.21 | 0.05 |
| PfRH1 | 0.20 | 0.09 | 0.02 |
| PfRH2 B240 | 0.20 | 0.18 | -0.05 |
| RH2 (2030) | 0.17 | 0.18 | -0.08 |
| RH4.2 | 0.21 | -0.00 | 0.25 |
| RH4.9 | 0.18 | 0.02 | -0.01 |
| SSP2 / TRAP | 0.18 | 0.06 | 0.09 |
| Cumulative Proportion | 0.63 | 0.67 | 0.70 |

**C**

|  | **rho** | **95% CI** | **P value** | **P adj** | **rho** | **95% CI** | **P value** | **P adj** |
| --- | --- | --- | --- | --- | --- | --- | --- | --- |
|  | **All volunteers** | | | | **Volunteers without prior malaria** | | | |
| **CSP FL** | 0.76 | 0.68;0.82 | **<0.001** | **<0.001** | 0.74 | 0.65;0.81 | **<0.001** | **<0.001** |
| **CSP C-term** | 0.49 | 0.35;0.6 | **<0.001** | **<0.001** | 0.46 | 0.31;0.58 | **<0.001** | **<0.001** |
| **CSP NANP** | 0.56 | 0.43;0.66 | **<0.001** | **<0.001** | 0.5 | 0.36;0.62 | **<0.001** | **<0.001** |
| **HBsAg** | 0.11 | -0.06;0.26 | 0.21 | 0.41 | 0.16 | -0.01;0.33 | 0.06 | 0.23 |
|  | **Kintampo volunteers** | | | | **Manhiça volunteers** | | | |
| **CSP FL** | 0.6 | 0.4;0.74 | **<0.001** | **<0.001** | 0.61 | 0.46;0.73 | **<0.001** | **<0.001** |
| **CSP C-term** | 0.51 | 0.29;0.68 | **<0.001** | **<0.001** | 0.33 | 0.12;0.5 | **0.002** | **0.02** |
| **CSP NANP** | 0.59 | 0.39;0.73 | **<0.001** | **<0.001** | 0.22 | 0.010.42 | **0.04** | 0.2 |
| **HBsAg** | 0.19 | -0.07;0.42 | 0.15 | 0.32 | 0.15 | -0.070.35 | 0.18 | 0.66 |

**D**

|  | **rho** | **95% CI** | **P value** | **P adj** | **rho** | **95% CI** | **P value** | **P adj** |
| --- | --- | --- | --- | --- | --- | --- | --- | --- |
|  | **All volunteers** | | | | **Volunteers with prior malaria** | | | |
| **CSP FL** | -0.18 | -0.32;-0.04 | **0.01** | **0.042** | 0.1 | -0.35;0.51 | 0.67 | 1 |
| **CSP C-term** | -0.13 | -0.27;0.01 | 0.06 | 0.18 | -0.09 | -0.51;0.35 | 0.68 | 1 |
| **CSP NANP** | -0.08 | -0.22;0.06 | 0.25 | 0.49 | 0.31 | -0.14;0.65 | 0.18 | 0.71 |
| **HBsAg** | -0.01 | -0.15;0.13 | 0.89 | 0.89 | -0.08 | -0.5;0.36 | 0.72 | 1 |
|  | **Kintampo volunteers** | | | | **Manhiça volunteers** | | | |
| **CSP FL** | -0.08 | -0.3;0.14 | 0.47 | 1 | 0.07 | -0.11;0.25 | 0.45 | 1 |
| **CSP C-term** | -0.22 | -0.42;0.01 | 0.057 | 0.23 | 0.1 | -0.08;0.28 | 0.26 | 1 |
| **CSP NANP** | 0.09 | -0.13;0.31 | 0.41 | 1 | 0.02 | -0.17;0.2 | 0.86 | 1 |
| **HBsAg** | -0.1 | -0.31;0.13 | 0.4 | 1 | 0.05 | -0.13;0.23 | 0.59 | 1 |
|  | **Infant volunteers** | | | | **Children volunteers** | | | |
| **CSP FL** | -0.06 | -0.24;0.13 | 0.56 | 1 | -0.62 | -0.74;-0.47 | **<0.001** | **<0.001** |
| **CSP C-term** | -0.01 | -0.2;0.18 | 0.91 | 1 | -0.41 | -0.57;-0.21 | **<0.001** | **<0.001** |
| **CSP NANP** | -0.01 | -0.2;0.18 | 0.92 | 1 | -0.23 | -0.42;-0.02 | **0.04** | 0.07 |
| **HBsAg** | 0.04 | -0.15;0.22 | 0.7 | 1 | -0.21 | -0.4;0.01 | 0.058 | 0.07 |
|  | **Infants Kintampo** | | | | **Children Kintampo** | | | |
| **CSP FL** | 0.01 | -0.3;0.32 | 0.94 | 1 | -0.58 | -0.76;-0.32 | **<0.001** | **<0.001** |
| **CSP C-term** | 0.04 | -0.27;0.35 | 0.80 | 1 | -0.57 | -0.76;-0.31 | **<0.001** | **<0.001** |
| **CSP NANP** | 0.04 | -0.28;0.35 | 0.82 | 1 | -0.37 | -0.61;-0.05 | **0.02** | **0.048** |
| **HBsAg** | -0.04 | -0.34;0.28 | 0.83 | 1 | -0.32 | -0.58;0 | **0.048** | **0.048** |
|  | **Infant Manhiça** | | | | **Children Manhiça** | | | |
| **CSP FL** | 0.04 | -0.2;0.28 | 0.72 | 1 | -0.24 | -0.49;0.04 | 0.1 | 0.39 |
| **CSP C-term** | 0.02 | -0.21;0.26 | 0.84 | 1 | 0.14 | -0.15;0.41 | 0.34 | 1 |
| **CSP NANP** | 0.08 | -0.16;0.31 | 0.53 | 1 | -0.05 | -0.33;0.24 | 0.76 | 1 |
| **HBsAg** | 0.12 | -0.12;0.34 | 0.34 | 1 | -0.11 | -0.38;0.18 | 0.45 | 1 |
|  | **Infants with prior malaria** | | | | **Children with prior malaria** | | | |
| **CSP FL** | 0.38 | -0.33;0.82 | 0.28 | 0.78 | -0.75 | -0.93;-0.28 | **0.007** | **0.03** |
| **CSP C-term** | 0.39 | -0.31;0.82 | 0.26 | 0.78 | -0.65 | -0.9;-0.09 | **0.03** | 0.09 |
| **CSP NANP** | 0.15 | -0.53;0.71 | 0.68 | 0.78 | -0.62 | -0.89;-0.03 | **0.043** | 0.09 |
| **HBsAg** | 0.54 | -0.14;0.87 | 0.11 | 0.43 | -0.17 | -0.7;0.48 | 0.61 | 0.61 |
|  | **Infants without prior malaria** | | | | **Children without prior malaria** | | | |
| **CSP FL** | -0.02 | -0.22;0.18 | 0.83 | 1 | -0.54 | -0.69;-0.36 | **<0.001** | **<0.001** |
| **CSP C-term** | -0.01 | -0.21;0.19 | 0.9 | 1 | -0.27 | -0.47;-0.04 | **0.02** | 0.06 |
| **CSP NANP** | 0.03 | -0.17;0.23 | 0.76 | 1 | -0.23 | 0.43;0 | **0.049** | 0.1 |
| **HBsAg** | 0 | -0.19;0.2 | 0.97 | 1 | -0.17 | -0.38;0.06 | 0.15 | 0.15 |

**Table S9. Effect of malaria maternal antibody index at baseline on RTS,S immunogenicity.** This was assessed in the subpopulation of subjects age <10 months at pre-vaccination, adjusted by site. M0 = month 0, M3 = month 3.

|  | | **RTS,S M3 (N=96)** | | | **RTS,S + Comparators M0 (N=145)** | | | **Comparators M3 (N=49)** | | |
| --- | --- | --- | --- | --- | --- | --- | --- | --- | --- | --- |
| **Isotype** | **Antigen** | **Coef (CI)** | **P** | **P-Adj** | **Coef (CI)** | **P** | **P-Adj** | **Coef (CI)** | **P** | **P-Adj** |
| IgG | CSP_FL | -10.64 (-22.98;3.67) | 0.14 | 0.68 | 27.19 (20.78;33.94) | **<0.001** | **<0.001** | 8.44 (-18.65;44.55) | 0.57 | 1 |
|  | CSP_Cterm | -13.74 (-24.08;-1.99) | **0.02** | 0.17 | 15.05 (8.77;21.68) | **<0.001** | **<0.001** | 7.22 (-16.39;37.5) | 0.58 | 1 |
|  | CSP_NANP | -15.08 (-26.92;-1.32) | **0.03** | 0.20 | 21.81 (13.28;30.99) | **<0.001** | **<0.001** | 7.32 (-19.35;42.81) | 0.62 | 1 |
|  | HBsAg | -17.85 (-27.96;-6.31) | **0.004** | **0.03** | 10.23 (2.55;18.49) | **0.009** | 0.07 | 2.18 (-12.35;19.13) | 0.78 | 1 |
| IgG1 | CSP_FL | -10.02 (-23.93;6.45) | 0.22 | 0.45 | 33.31 (21.78;45.93) | **<0.001** | **<0.001** | 14.78 (-19.08;62.81) | 0.43 | 0.69 |
|  | CSP_Cterm | -17.04 (-29.11;-2.91) | **0.02** | 0.07 | 12.48 (2.89;22.95) | **0.01** | **0.044** | 13.25 (-14.43;49.89) | 0.38 | 0.64 |
|  | CSP_NANP | -16.51 (-30.24;-0.07) | **0.049** | 0.16 | 24.49 (12.29;38.01) | **<0.001** | **<0.001** | 13.07 (-20.91;61.64) | 0.49 | 0.76 |
|  | HBsAg | -20.25 (-30.21;-8.86) | **0.001** | **0.007** | 7.63 (-1.07;17.09) | 0.09 | 0.25 | 2.46 (-13.73;21.69) | 0.78 | 0.93 |
| IgG2 | CSP_FL | -4.49 (-11.55;3.13) | 0.24 | 0.48 | 26.93 (16.12;38.74) | **<0.001** | **<0.001** | 5.74 (-15.65;32.54) | 0.62 | 0.85 |
|  | CSP_Cterm | 3.57 (-7.8;16.34) | 0.55 | 0.80 | 10.61 (4.75;16.81) | **<0.001** | **0.003** | 1.96 (-14.5;21.6) | 0.82 | 0.94 |
|  | CSP_NANP | -4.26 (-12.95;5.29) | 0.37 | 0.64 | 22.44 (12.55;33.19) | **<0.001** | **<0.001** | 4.76 (-11.54;24.06) | 0.58 | 0.82 |
|  | HBsAg | 0.69 (-8.37;10.64) | 0.89 | 0.95 | 18.08 (7.12;30.17) | **<0.001** | **0.007** | -4.63 (-13.73;5.43) | 0.35 | 0.64 |
| IgG3 | CSP_FL | -10.35 (-24.38;6.28) | 0.21 | 0.45 | 37.59 (23.46;53.34) | **<0.001** | **<0.001** | -0.28 (-28.34;38.75) | 0.99 | 0.99 |
|  | CSP_Cterm | -4.46 (-16.6;9.44) | 0.51 | 0.76 | 7.09 (-2.72;17.88) | 0.16 | 0.39 | 4.39 (-16.53;30.57) | 0.70 | 0.88 |
|  | CSP_NANP | -18.85 (-31.36;-4.06) | **0.02** | 0.06 | 18.72 (5.74;33.3) | **0.004** | **0.02** | -0.24 (-25.68;33.91) | 0.99 | 0.99 |
|  | HBsAg | -7.46 (-14.58;0.25) | 0.06 | 0.17 | -15.79 (-25.06;-5.37) | **0.004** | **0.02** | -9.19 (-28.86;15.9) | 0.43 | 0.69 |
| IgG4 | CSP_FL | 2.66 (-9.72;16.74) | 0.69 | 0.88 | -0.5 (-7.12;6.59) | 0.89 | 0.95 | 1.92 (-12.76;19.07) | 0.81 | 0.94 |
|  | CSP_Cterm | 9.14 (-3.77;23.79) | 0.17 | 0.39 | -1.09 (-7.14;5.35) | 0.73 | 0.90 | -2.45 (-14.14;10.83) | 0.70 | 0.88 |
|  | CSP_NANP | -8.24 (-18.37;3.14) | 0.15 | 0.39 | 0.07 (-6.4;6.99) | 0.98 | 0.99 | 1.04 (-11.85;15.81) | 0.88 | 0.95 |
|  | HBsAg | -2.49 (-4.46;-0.47) | **0.02** | 0.06 | 0.64 (-0.74;2.03) | 0.36 | 0.64 | 1.28 (-0.53;3.13) | 0.16 | 0.39 |
| IgM | CSP_FL | -4.77 (-13.33;4.64) | 0.31 | 1 | -0.56 (-7.9;7.37) | 0.89 | 1 | 4.66 (-9.61;21.19) | 0.53 | 1 |
|  | CSP_Cterm | 1.24 (-6.75;9.91) | 0.77 | 1 | 1.89 (-5.51;9.88) | 0.62 | 1 | 7.51 (-6.54;23.67) | 0.30 | 1 |
|  | CSP_NANP | -0.41 (-10.57;10.9) | 0.94 | 1 | 5.81 (-2.98;15.4) | 0.20 | 1 | 0.11 (-15.41;18.48) | 0.99 | 1 |
|  | HBsAg | -11.95 (-21.18;-1.64) | **0.02** | 0.25 | -20.15 (-26.5;-13.26) | **< 0.001** | **<0.001** | -21.54 (-31.36;-10.32) | **<0.001** | **0.007** |

**Table S10. Effect of malaria exposure antibody index at baseline on RTS,S immunogenicity.** Adjusted by site. M0 = month 0, M3 = month 3.

|  | | **RTS,S M3 (N=129)** | | | **RTSS + Comparators M0 (N=195)** | | | **Comparators M3 (N=66)** | | |
| --- | --- | --- | --- | --- | --- | --- | --- | --- | --- | --- |
| **Isotype** | **Antigen** | **Coef (CI)** | **P** | **P-Adj** | **Coef (CI)** | **P** | **P-Adj** | **Coef (CI)** | **P** | **P-Adj** |
| IgG | CSP_FL | 4.68 (-2.42;12.3) | 0.20 | 1 | 2.82 (-1.21;7.01) | 0.17 | 1 | 1.09 (-12.33;16.56) | 0.88 | 1 |
|  | CSP_Cterm | 5.3 (-1.08;12.1) | 0.1 | 0.94 | 3.2 (-0.11;6.61) | 0.058 | 0.58 | 4.84 (-7.22;18.46) | 0.44 | 1 |
|  | CSP_NANP | 8.12 (0.5;16.32) | **0.04** | 0.40 | 0.26 (-4.17;4.9) | 0.91 | 1 | -1.28 (-14.96;14.61) | 0.86 | 1 |
|  | HBsAg | 14.08 (6.24;22.5) | **<0.001** | **0.004** | 1.79 (-2.41;6.17) | 0.41 | 1 | 6.9 (-2.47;17.16) | 0.15 | 1 |
| IgG1 | CSP_FL | 3.99 (-3.88;12.51) | 0.33 | 0.68 | 1.66 (-3.91;7.56) | 0.56 | 0.82 | -0.71 (-16.74;18.42) | 0.94 | 0.98 |
|  | CSP_Cterm | 6.54 (-1.49;15.23) | 0.11 | 0.36 | 2.35 (-2.32;7.25) | 0.33 | 0.68 | 1.75 (-11.52;17.01) | 0.80 | 0.95 |
|  | CSP_NANP | 9.24 (0.29;18.98) | **0.043** | 0.21 | -2.3 (-7.97;3.72) | 0.44 | 0.73 | -1.99 (-18.8;18.29) | 0.83 | 0.95 |
|  | HBsAg | 14.96 (6.67;23.88) | **<0.001** | **0.01** | 1.44 (-3.38;6.51) | 0.56 | 0.82 | 6.36 (-4.23;18.11) | 0.24 | 0.56 |
| IgG2 | CSP_FL | -3.28 (-7.2;0.8) | 0.11 | 0.36 | -3.39 (-8.01;1.46) | 0.17 | 0.46 | -0.86 (-10.81;10.21) | 0.87 | 0.95 |
|  | CSP_Cterm | 1.22 (-4.44;7.21) | 0.68 | 0.88 | -0.24 (-3.21;2.81) | 0.87 | 0.95 | 1.03 (-6.81;9.53) | 0.80 | 0.95 |
|  | CSP_NANP | -3.51 (-8.07;1.29) | 0.15 | 0.44 | -7.65 (-11.66;-3.45) | **<0.001** | **0.01** | -2.53 (-10.09;5.66) | 0.53 | 0.82 |
|  | HBsAg | 0.69 (-4.78;6.48) | 0.81 | 0.95 | -2.1 (-7.23;3.32) | 0.44 | 0.73 | 10.37 (2.06;19.36) | **0.01** | 0.12 |
| IgG3 | CSP_FL | 7.75 (-0.69;16.91) | 0.07 | 0.27 | 6.17 (-0.59;13.38) | 0.07 | 0.27 | 3.81 (-12.41;23.03) | 0.66 | 0.88 |
|  | CSP_Cterm | 9.6 (2.57;17.11) | **0.007** | 0.09 | 3.48 (-1.88;9.12) | 0.21 | 0.49 | 4.42 (-6.44;16.55) | 0.43 | 0.73 |
|  | CSP_NANP | 9.94 (0.93;19.75) | **0.03** | 0.20 | 4.49 (-2.18;11.61) | 0.19 | 0.48 | -3.94 (-17.7;12.11) | 0.60 | 0.83 |
|  | HBsAg | 6.67 (1.89;11.66) | **0.006** | 0.09 | -1.64 (-7.52;4.6) | 0.60 | 0.83 | 9.48 (-3.96;24.8) | 0.17 | 0.46 |
| IgG4 | CSP_FL | 8.95 (1.72;16.68) | **0.01** | 0.12 | -0.02 (-3.71;3.8) | 0.99 | 0.99 | -0.83 (-8.55;7.54) | 0.84 | 0.95 |
|  | CSP_Cterm | 8.25 (0.48;16.62) | **0.04** | 0.20 | -0.99 (-4.19;2.31) | 0.55 | 0.82 | -2.8 (-8.92;3.74) | 0.39 | 0.73 |
|  | CSP_NANP | 2.75 (-3.64;9.57) | 0.40 | 0.73 | 3.91 (0.24;7.71) | **0.04** | 0.20 | -2.85 (-9.32;4.08) | 0.41 | 0.73 |
|  | HBsAg | 1.38 (-0.05;2.82) | 0.058 | 0.25 | -0.05 (-0.86;0.77) | 0.90 | 0.96 | 0.01 (-1.2;1.24) | 0.99 | 0.99 |
| IgM | CSP_FL | 3.76 (-1.97;9.82) | 0.20 | 1 | 21.26 (17.65;24.99) | **<0.001** | **<0.001** | 3.81 (-3.59;11.79) | 0.32 | 1 |
|  | CSP_Cterm | 1.13 (-3.28;5.75) | 0.62 | 1 | 23.61 (20.53;26.76) | **<0.001** | **<0.001** | 3.79 (-3.42;11.54) | 0.31 | 1 |
|  | CSP_NANP | 4.64 (-1.37;11.01) | 0.13 | 0.79 | 19.92 (15.69;24.31) | **<0.001** | **<0.001** | 3.58 (-4.85;12.76) | 0.41 | 1 |
|  | HBsAg | 13.4 (6.65;20.58) | **<0.001** | **< 0.001** | 22.23 (15.91;28.9) | **<0.001** | **< 0.001** | 16.26 (6.44;26.99) | **0.001** | **0.008** |

**Table S11. Factors affecting the immunogenicity of RTS,S/AS01E in infants with maternal antibodies.** Multivariable linear models in the subset of infants age <10 months, including RTS,S/AS01E vaccinees at month 3. The coefficients indicate % change for a unit change in the predictor (95% confidence intervals), the p values indicated are for statistically significant covariates (in bold) and for those that improved the model.

| **Isotype** | **Antigen** | **Age *** | **Site** | **Prior episode**^†^ | **Season** | **Baseline Ig** | **Maternal** | **Exposure** | **Sex** | **Hb** | **WAZ** | **HAZ** |
| --- | --- | --- | --- | --- | --- | --- | --- | --- | --- | --- | --- | --- |
| **IgG** | **CSP FL** |  |  |  |  |  | -9.35 (-19.6;2.2), 0.11 |  |  |  | -23.34 (-47.26;11.42), 0.16 |  |
|  | **CSP C-term** |  |  | 154.42 (-9.11;612.15), 0.07 |  |  | -12.91 (-21.8;-3.01), **0.01** |  |  |  | -31.27 (-50.16;-5.23), 0.02 |  |
|  | **CSP NANP** | 3.02 (-0.45;6.62), 0.09 |  |  |  | -54.4 (-74.71;-17.79), **0.01** |  |  |  | - |  |  |
|  | **HBsAg** | 8.69 (5.77;11.68), < **0.001** | 126.36 (17.6;335.7), **0.02** | 169.89 (-0.57;632.57), 0.051 |  |  |  |  |  |  |  |  |
| **IgG1** | **CSP FL** |  |  |  |  |  |  |  |  |  | -29.68 (-53.54;6.45), 0.1 |  |
|  | **CSP C-term** |  |  | 223.91 (-7.95;1039.82), 0.07 |  |  | -17.98 (-28.09;-6.44), **0.004** |  |  |  | -36.76 (-57.3;-6.34), 0.02 |  |
|  | **CSP NANP** |  |  | 190.51 (- 30.42;1112.93), 0.14 |  | -41.58 (-67.88;6.24), 0.08 | -12.3 (-25.45;3.18), 0.11 |  |  |  | -38.51 (-60.68;-3.85), 0.03 |  |
|  | **HBsAg** | 10 (7.1;12.98), **< 0.001** | 144.95 (23.41;386.19), **0.01** | 131.47 (-15.86;536.79), 0.1 |  |  |  |  |  |  | -19.07 (-40.24;9.58), 0.17 |  |
| **IgG2** | **CSP FL** | -2.57 (-4.6;-0.49), **0.02** | -75.11 (-85.08;-58.48), **<0.001** |  |  |  | -9.23 (-16.65;-1.15), **0.03** |  |  |  |  |  |
|  | **CSP C-term** | -2.19 (-4.95;0.65), 0.13 | -69.83 (-83.22;-45.78), **<0.001** |  |  |  |  |  | -36.2 (-64.33;14.1), 0.13 |  |  |  |
|  | **CSP NANP** | 1.92 (-0.73;4.65), 0.16 | -49.77 (-72.69;-7.63), **0.03** |  |  | 42.41 (-8.13;120.75), 0.11 |  | -5.47 (-10.67;0.03), 0.051 |  |  | -24.75 (-41.28;-3.57), 0.03 |  |
|  | **HBsAg** | 3.19 (1.52;4.88), **<0.001** |  |  |  | 275.02 (195.44;376.04), **<0.001** |  |  | 36.51 (0.79;84.88), **0.044** |  |  |  |
| **IgG3** | **CSP FL** |  |  | 311.5 (4.48;1520.65), **0.043** |  |  | -10.78 (-22.69;2.98), 0.12 |  |  |  | -33.03 (-56.35;2.73), 0.07 |  |
|  | **CSP C-term** |  |  |  |  |  |  | 6.97 (-0.6;15.13), 0.07 |  |  |  |  |
|  | **CSP NANP** |  |  | 170.29 (-30.82;956.09), 0.15 |  |  | -17.03 (-27.87;-4.56), **0.01** | 11.39 (1.22;22.58), **0.03** |  |  |  |  |
|  | **HBsAg** | 4.47 (2.75;6.23), **<0.001** |  |  |  |  |  |  |  |  | 16.13 (-2.82;38.77), 0.1 |  |
| **IgG4** | **CSP FL** |  | -64.28 (-81.53;-30.9), **0.003** |  |  |  |  | 9.79 (2.16;18), **0.01** |  |  |  |  |
|  | **CSP C-term** |  |  |  | 441.16 (-34.8;4391.39), 0.12 | -44.45 (-73.27;15.45), 0.11 |  | 7.08 (0.09;14.57), **0.047** | -35.22 (-64.8;19.24), 0.16 |  |  |  |
|  | **CSP NANP** |  |  | 412.42 (107.92;1162.9), **<0.001** |  |  | -7.9 (-16.1;1.09), 0.08 |  |  |  |  |  |
|  | **HBsAg** | 0.99 (0.52;1.45), **<0.001** | 14.72 (1.67;29.45), **0.03** | 15.54 (-2.96;37.56), 0.1 |  | 50.16 (-5.23;137.94), 0.08 |  |  |  |  | 7.59 (1.04;14.57), 0.02 | -6.2 (-11.61;-0.45), 0.04 |
| **IgM** | **CSP FL** | 1.72 (-0.46;3.95), 0.12 |  | 68.65 (-17.83;246.14), 0.15 |  |  |  |  |  |  |  | -19.06 (-34.37;-0.18), 0.048 |
|  | **CSP C-term** |  | 54.53 (-6.55;155.54), 0.09 | 92.46 (-8.56;305.06), 0.08 |  |  |  |  |  | -12.1 (-24.46;2.28), 0.09 |  | -16.31 (-31.39;2.09), 0.08 |
|  | **CSP NANP** | 1.98 (-0.51;4.53), 0.12 |  |  |  |  |  |  |  |  |  | -18.5 (-35.71;3.32), 0.09 |
|  | **HBsAg** | 7.25 (4.98;9.56), **< 0.001** | 362.86 (167.57;700.7), **<0.001** | 301.09 (79.86;794.43), **<0.001** |  |  |  |  |  | -23.6 (-35.44;-9.59), **0.002** |  | -24.9 (-39.65;-6.55), 0.01 |

*Continuous age at weeks. Site (Manhiça vs Kintampo). ^†^Clinical malaria episode between month 0 and month 3 (yes vs no). Malaria transmission season at month 3 sample collection (low vs high). Baseline antibodies to the same Ig/antigen. Baseline anti-*P. falciparum* maternal IgG levels (maternal PC1 index). Baseline anti-*P. falciparum* exposure IgM levels (exposure PC1 index). Sex (male vs female). Baseline hemoglobin (Hb, g/dL). Weight-for-Age Z-scores (WAZ). Height-for-Age Z-scores (HAZ).

**Table S12. Univariate crude effect of baseline antibody levels to RTS,S antigens on clinical malaria protection**. Association of levels (log_10_ MFI) of antibodies at month 0 with odds of clinical malaria in a 12-month follow-up period by univariate logistic regression models. P values were adjusted as explained in the Methods section.

| **Antibody** | **Antigen** | **All Vaccinees** | | | **RTS,S/AS01E Vaccinees** | | | **Comparator Vaccinees** | | |
| --- | --- | --- | --- | --- | --- | --- | --- | --- | --- | --- |
|  |  | **OR (95% CI)** | **P** | **P-Adj** | **OR (95% CI)** | **P** | **P-Adj** | **OR (95% CI)** | **P** | **P-Adj** |
| IgG | CSP FL | 4.56 (2.49 ; 8.86) | **<0.001** | **<0.001** | 4.84 (2.32; 11.11) | **<0.001** | **<0.001** | 4.03 (1.44; 12.99) | **0.007** | 0.02 |
|  | CSP Cterm | 4.96 (2.36 ; 11.08) | **<0.001** | **<0.001** | 6.75 (2.58; 19.52) | **<0.001** | **<0.001** | 3.05 (1 ; 10.88) | **0.051** | 0.1 |
|  | CSP NANP | 2.73 (1.61 ; 4.84) | **<0.001** | **<0.001** | 3.03 (1.61 ; 6.11) | **<0.001** | **<0.001** | 2.13 (0.81 ; 6.08) | 0.13 | 0.13 |
|  | HBsAg | 1.19 (0.68 ; 2.09) | 0.55 | 1 | 0.68 (0.33 ; 1.36) | 0.28 | 1 | 4.06 (1.38; 14.81) | **0.009** | 0.057 |
| IgM | CSP FL | 1.83 (1.02 ; 3.32) | **0.041** | **0.041** | 1.75 (0.87 ; 3.59) | 0.11 | 0.11 | 2.01 (0.71 ; 6.2) | 0.19 | 0.33 |
|  | CSP Cterm | 2.39 (1.29 ; 4.56) | **0.005** | **0.02** | 2.27 (1.1 ; 4.92) | **0.03** | 0.08 | 2.69 (0.88 ; 9.41) | 0.08 | 0.25 |
|  | CSP NANP | 2.03 (1.18 ; 3.59) | **0.01** | **0.02** | 2.03 (1.07 ; 4.01) | **0.03** | 0.08 | 2.03 (0.75 ; 6.01) | 0.17 | 0.33 |
|  | HBsAg | 0.84 (0.57 ; 1.25) | 0.39 | 1 | 0.65 (0.39 ; 1.06) | 0.09 | 0.51 | 1.40 (0.71 ; 2.83) | 0.34 | 1 |
| IgG1 | CSP FL | 2.53 (1.69 ; 3.89) | **<0.001** | **<0.001** | 2.85 (1.72 ; 4.96) | **<0.001** | **<0.001** | 2.05 (1.07 ; 4.19) | **0.03** | 0.14 |
|  | CSP Cterm | 2.14 (1.3 ; 3.61) | **0.003** | **0.006** | 2.00 (1.08 ; 3.83) | **0.03** | **0.048** | 2.43 (1.05 ; 6.08) | **0.04** | 0.14 |
|  | CSP NANP | 2.31 (1.54 ; 3.56) | **<0.001** | **<0.001** | 2.64 (1.59 ; 4.64) | **<0.001** | **<0.001** | 1.79 (0.91 ; 3.72) | 0.09 | 0.19 |
|  | HBsAg | 1.58 (0.97 ; 2.62) | 0.06 | 0.38 | 0.93 (0.5 ; 1.72) | 0.81 | 1 | 5.43 (1.97; 18.85) | **<0.001** | **0.003** |
| IgG2 | CSP FL | 2.87 (1.7 ; 5.09) | **<0.001** | **<0.001** | 2.92 (1.59 ; 5.71) | **<0.001** | **0.002** | 2.73 (1.01 ; 9.02) | **0.047** | 0.14 |
|  | CSP Cterm | 1.80 (0.79 ; 4.35) | 0.16 | 0.24 | 2.37 (0.9 ; 6.92) | 0.08 | 0.12 | 0.85 (0.15 ; 4.52) | 0.84 | 0.99 |
|  | CSP NANP | 2.39 (1.4 ; 4.26) | **0.001** | **0.003** | 2.16 (1.19 ; 4.11) | **0.01** | **0.03** | 3.56 (1.11 ; 14.7) | **0.03** | 0.14 |
|  | HBsAg | 0.87 (0.55 ; 1.36) | 0.55 | 1 | 0.71 (0.4 ; 1.23) | 0.23 | 1 | 1.35 (0.6 ; 3.13) | 0.47 | 1 |
| IgG3 | CSP FL | 1.60 (1.12 ; 2.34) | **0.01** | **0.02** | 2.02 (1.27 ; 3.32) | **0.003** | **0.008** | 1.12 (0.65 ; 2.01) | 0.68 | 0.98 |
|  | CSP Cterm | 1.09 (0.7 ; 1.71) | 0.69 | 0.69 | 1.17 (0.64 ; 2.15) | 0.60 | 0.72 | 1.00 (0.51 ; 2) | 0.99 | 0.99 |
|  | CSP NANP | 1.39 (0.97 ; 2.02) | 0.07 | 0.12 | 1.65 (1.06 ; 2.64) | **0.03** | **0.048** | 1.02 (0.57 ; 1.88) | 0.94 | 0.99 |
|  | HBsAg | 0.94 (0.63 ; 1.38) | 0.74 | 1 | 0.83 (0.44 ; 1.41) | 0.49 | 1 | 1.07 (0.61 ; 1.92) | 0.82 | 1 |
| IgG4 | CSP FL | 1.14 (0.61 ; 2.13) | 0.69 | 0.69 | 1.32 (0.62 ; 2.84) | 0.47 | 0.63 | 0.83 (0.27 ; 2.49) | 0.73 | 0.98 |
|  | CSP Cterm | 1.60 (0.79 ; 3.27) | 0.19 | 0.25 | 1.16 (0.47 ; 2.79) | 0.75 | 0.82 | 2.98 (0.89; 11.66) | 0.08 | 0.19 |
|  | CSP NANP | 1.24 (0.66 ; 2.36) | 0.50 | 0.60 | 1.00 (0.45 ; 2.15) | 1 | 1 | 2.04 (0.65 ; 6.99) | 0.23 | 0.39 |
|  | HBsAg | 0.03 (0 ; 0.8) | **0.04** | 0.25 | 0.02 (0 ; 1) | **0.05** | 0.35 | 0.08 (0 ; 23.06) | 0.38 | 1 |

**Table S13. Univariate crude effect of all covariates on clinical malaria protection**. Association of demographic and clinical predictors with odds of clinical malaria in a 12-month follow-up period obtained in logistic regression models.

| **Predictor** | **All Vaccinees** | | | **RTS,S/AS01E Vaccinees** | | | **Comparator Vaccinees** | | |
| --- | --- | --- | --- | --- | --- | --- | --- | --- | --- |
|  | **OR** | **95% CI** | **P value** | **OR** | **95% CI** | **P value** | **OR** | **95% CI** | **P value** |
|  | ***Crude Analysis*** | | | | | | | | |
| Trial age cohort (5-17 months vs 6-12 weeks) | 0.697 | 0.39; 1.23 | 0.22 | 0.529 | 0.25; 1.08 | 0.08 | 1.166 | 0.44; 3.12 | 0.76 |
| Continuous age at weeks | 0.995 | 0.98; 1.01 | 0.47 | 0.99 | 0.97; 1 | 0.19 | 1.007 | 0.99; 1.03 | 0.53 |
| Site (Manhiça vs Kintampo) | 0.098 | 0.05; 0.19 | **<0.001** | 0.115 | 0.05; 0.25 | **<0.001** | 0.068 | 0.02; 0.22 | **<0.001** |
| Malaria episodes before M3 (yes vs no) | 4.305 | 1.6; 13.69 | **0.003** | 8.295 | 2.07; 55.61 | **0.002** | 1.931 | 0.46; 9.86 | 0.37 |
| Malaria transmission season at M3 (Low vs high) | 0.505 | 0.12; 1.81 | 0.30 | 1.396 | 0.05; 35.84 | 0.82 | 0.391 | 0.08; 1.63 | 0.20 |
| Baseline anti-*P. falciparum* exposure IgM levels (exposure PC1 index) | 1.162 | 1.08; 1.26 | **<0.001** | 1.118 | 1.03; 1.23 | **0.01** | 1.322 | 1.12; 1.64 | **<0.001** |
| Baseline anti-P*. falciparum* maternal IgG levels* (maternal PC1 index) | 1.399 | 1.23; 1.61 | **<0.001** | 1.39 | 1.19; 1.65 | **<0.001** | 1.419 | 1.13; 1.86 | **0.002** |
| Sex (Male vs Female) | 1.305 | 0.74; 2.32 | 0.36 | 1.217 | 0.6; 2.47 | 0.58 | 1.495 | 0.56; 4.05 | 0.42 |
| Weight-for-Age Z-score | 0.732 | 0.56; 0.95 | **0.02** | 0.77 | 0.54; 1.08 | 0.13 | 0.675 | 0.43; 1.03 | 0.07 |
| Height-for-Age Z-score | 0.74 | 0.57; 0.95 | **0.02** | 0.745 | 0.53; 1.08 | 0.08 | 0.734 | 0.48; 1.08 | 0.12 |
| Hemoglobin (g/dL) | 0.903 | 0.73; 1.11 | 0.33 | 0.942 | 0.73; 1.21 | 0.64 | 0.835 | 0.58; 1.18 | 0.31 |
|  | ***Adjusted by site*** | | | | | | | | |
| Trial age cohort (5-17 months vs 6-12 weeks) | 0.48 | 0.23; 0.95 | **0.04** | 0.351 | 0.14; 0.82 | **0.02** | 0.882 | 0.26; 2.89 | 0.84 |
| Continuous age at weeks | 0.991 | 0.98; 1.01 | 0.24 | 0.984 | 0.97; 1 | 0.08 | 1.007 | 0.98; 1.03 | 0.60 |
| Malaria episodes before M3 (yes vs no) | 1.296 | 0.42; 4.55 | 0.66 | 2.308 | 0.5; 16.54 | 0.30 | 0.575 | 0.08; 4.07 | 0.57 |
| Malaria transmission season at M3 (Low vs high) | 1.455 | 0.34; 5.54 | 0.59 | 3.529 | 0.13; 92.44 | 0.39 | 1.312 | 0.23; 6.37 | 0.74 |
| Baseline anti-*P. falciparum* exposure IgM levels (exposure PC1 index) | 1.101 | 1.01; 1.21 | **0.03** | 1.04 | 0.94; 1.15 | 0.44 | 1.504 | 1.16; 2.1 | **<0.001** |
| Baseline anti-P*. falciparum* maternal IgG levels* (maternal PC1 index) | 1.228 | 1.06; 1.44 | **0.007** | 1.222 | 1.02; 1.49 | **0.03** | 1.255 | 0.97; 1.68 | 0.09 |
| Sex (Male vs Female) | 1.414 | 0.73; 2.78 | 0.31 | 1.586 | 0.71; 3.69 | 0.27 | 1.034 | 0.3; 3.41 | 0.96 |
| Weight-for-Age Z-score | 0.841 | 0.62; 1.14 | 0.26 | 0.987 | 0.66; 1.46 | 0.95 | 0.626 | 0.36; 1.03 | 0.07 |
| Height-for-Age Z-score | 0.86 | 0.64; 1.15 | 0.31 | 0.978 | 0.66; 1.46 | 0.91 | 0.703 | 0.43; 1.1 | 0.13 |
| Hemoglobin (g/dL) | 0.982 | 0.77; 1.25 | 0.88 | 1.052 | 0.79; 1.4 | 0.73 | 0.831 | 0.53; 1.29 | 0.40 |

*Only in infants age < 10 months at pre-vaccination. Outcomes for the other covariates in this subpopulation do not markedly change compared to the whole population (data not shown).

**Table S14. Machine learning analysis stratified by age and site in RTS,S vaccinees.**

**A)** **Elastic Net**

|  | **Kintampo RTS,S** | |  | **Manhiça** **RTS,S** | |  | **Children RTS,S** | |  | **Infants RTS,S** | |
| --- | --- | --- | --- | --- | --- | --- | --- | --- | --- | --- | --- |
|  | Variable | Coefficient |  | Variable | Coefficient |  | Variable | Coefficient |  | Variable | Coefficient |
|  | IgG1 NANP M0 | 0.43 |  | IgG C-term M0 | 0.51 |  | IgM Pf M0 | 0.74 |  | IgG2 C-term M3 | 0.29 |
|  | IgG3 NANP M0 | 0.32 |  | IgM NANP M3 | 0.37 |  | IgG3 NANP M0 | 0.28 |  | IgG C-term M0 | 0.24 |
|  | IgG2 NANP M3 | 0.19 |  | IgM C-term M0 | 0.26 |  | IgG2 C-term M3 | 0.24 |  | IgG1 NANP M0 | 0.21 |
|  | IgG2 HBsAg M3 | 0.12 |  | IgG1 NANP M3 | 0.22 |  | IgG2 NANP M3 | 0.22 |  | IgG3 C-term M0 | 0.21 |
|  | IgG NANP M0 | 0.11 |  | IgG2 C-term M3 | 0.19 |  | IgG1 NANP M0 | 0.05 |  | IgG2 NANP M3 | 0.18 |
|  | IgG4 NANP M3 | 0.05 |  | IgG3 C-term M3 | 0.16 |  | IgG4 NANP M3 | 0.03 |  | IgM NANP M0 | 0.17 |
|  | IgG2 C-term M3 | 0.01 |  | IgG3 C-term M0 | 0.14 |  | IgM NANP M3 | 0 |  | IgM C-term M0 | 0.17 |
|  | IgG HBsAg M0 | -0.33 |  | IgG1 C-term M0 | 0.13 |  | IgG C-term M3 | -0.86 |  | IgM NANP M3 | 0.17 |
|  | IgG C-term M3 | -0.15 |  | IgG1 NANP M0 | 0.07 |  | IgG3 HBsAg M0 | -0.75 |  | IgG Pf M0 | 0.14 |
|  | IgG1 C-term M3 | -0.13 |  | IgG3 NANP M0 | 0.05 |  | IgG3 HBsAg M3 | -0.25 |  | IgG NANP M0 | 0.14 |
|  | IgG3 HBsAg M3 | -0.11 |  | IgG HBsAg M3 | -0.52 |  | IgG HBsAg M0 | -0.12 |  | IgG1 C-term M0 | 0.13 |
|  | IgG3 HBsAg M0 | -0.08 |  | IgG4 HBsAg M3 | -0.44 |  | IgG NANP M0 | -- |  | IgG4 C-term M0 | 0.12 |
|  | IgG4 HBsAg M0 | -0.02 |  | IgG1 HBsAg M3 | -0.3 |  | IgG2 NANP M0 | -- |  | IgG3 NANP M0 | 0.1 |
|  | IgM Pf M0 | -- |  | IgG3 HBsAg M3 | -0.22 |  | IgM NANP M0 | -- |  | IgG1 NANP M3 | 0.08 |
|  | IgG2 NANP M0 | -- |  | IgG2 NANP M0 | -0.2 |  | IgG4 NANP M0 | -- |  | IgG4 NANP M3 | 0.07 |
|  | IgM NANP M0 | -- |  | IgG4 HBsAg M0 | -0.19 |  | IgG C-term M0 | -- |  | IgG3 C-term M3 | 0.02 |
|  | IgG4 NANP M0 | -- |  | IgG4 NANP M0 | -0.19 |  | IgG1 C-term M0 | -- |  | IgG2 NANP M0 | 0.01 |
|  | IgG C-term M0 | -- |  | IgG1 HBsAg M0 | -0.15 |  | IgG2 C-term M0 | -- |  | IgM C-term M3 | 0.01 |
|  | IgG1 C-term M0 | -- |  | IgG3 NANP M3 | -0.07 |  | IgM C-term M0 | -- |  | IgG4 HBsAg M0 | -0.21 |
|  | IgG2 C-term M0 | -- |  | IgG2 HBsAg M0 | -0.05 |  | IgG4 C-term M0 | -- |  | IgM HBsAg M3 | -0.2 |
|  | IgM C-term M0 | -- |  | IgM HBsAg M3 | -0.01 |  | IgG3 C-term M0 | -- |  | IgG HBsAg M0 | -0.19 |
|  | IgG4 C-term M0 | -- |  | IgM Pf M0 | -- |  | IgG1 HBsAg M0 | -- |  | IgG3 HBsAg M3 | -0.15 |
|  | IgG3 C-term M0 | -- |  | IgG NANP M0 | -- |  | IgG2 HBsAg M0 | -- |  | IgG4 HBsAg M3 | -0.08 |
|  | IgG1 HBsAg M0 | -- |  | IgM NANP M0 | -- |  | IgM HBsAg M0 | -- |  | IgG2 HBsAg M0 | -0.07 |
|  | IgG2 HBsAg M0 | -- |  | IgG2 C-term M0 | -- |  | IgG4 HBsAg M0 | -- |  | IgG HBsAg M3 | -0.06 |
|  | IgM HBsAg M0 | -- |  | IgG4 C-term M0 | -- |  | IgG NANP M3 | -- |  | IgG3 NANP M3 | -0.01 |
|  | IgG NANP M3 | -- |  | IgG HBsAg M0 | -- |  | IgG1 NANP M3 | -- |  | IgM Pf M0 | -- |
|  | IgG1 NANP M3 | -- |  | IgM HBsAg M0 | -- |  | IgG3 NANP M3 | -- |  | IgG4 NANP M0 | -- |
|  | IgM NANP M3 | -- |  | IgG3 HBsAg M0 | -- |  | IgG1 C-term M3 | -- |  | IgG2 C-term M0 | -- |
|  | IgG3 NANP M3 | -- |  | IgG NANP M3 | -- |  | IgM C-term M3 | -- |  | IgG1 HBsAg M0 | -- |
|  | IgM C-term M3 | -- |  | IgG2 NANP M3 | -- |  | IgG4 C-term M3 | -- |  | IgM HBsAg M0 | -- |
|  | IgG4 C-term M3 | -- |  | IgG4 NANP M3 | -- |  | IgG3 C-term M3 | -- |  | IgG3 HBsAg M0 | -- |
|  | IgG3 C-term M3 | -- |  | IgG C-term M3 | -- |  | IgG HBsAg M3 | -- |  | IgG NANP M3 | -- |
|  | IgG HBsAg M3 | -- |  | IgG1 C-term M3 | -- |  | IgG1 HBsAg M3 | -- |  | IgG C-term M3 | -- |
|  | IgG1 HBsAg M3 | -- |  | IgM C-term M3 | -- |  | IgG2 HBsAg M3 | -- |  | IgG1 C-term M3 | -- |
|  | IgM HBsAg M3 | -- |  | IgG4 C-term M3 | -- |  | IgM HBsAg M3 | -- |  | IgG4 C-term M3 | -- |
|  | IgG4 HBsAg M3 | -- |  | IgG2 HBsAg M3 | -- |  | IgG4 HBsAg M3 | -- |  | IgG1 HBsAg M3 | -- |

**B) Supported Vector Machines**

Children RTS,S Infants RTS,S

Kintampo RTS,S Manhiça RTS,S

**C) Random forest**

Children RTS,S Infants RTS,S

Kintampo RTS,S Manhiça RTS,S

**Supplementary References**

1. Ubillos I, Campo JJ, Jimenez A, Dobano C: Development of a high-throughput flexible quantitative suspension array assay for IgG against multiple Plasmodium falciparum antigens. Malar J. 2018;17(1):216.

2. Vidal M, Aguilar R, Campo JJ, Dobano C: Development of quantitative suspension array assays for six immunoglobulin isotypes and subclasses to multiple Plasmodium falciparum antigens. J Immunol Methods. 2018;455:41-54.

3. Ubillos I, Jimenez A, Vidal M, Bowyer PW, Gaur D, Dutta S, Gamain B, Coppel R, Chauhan V, Lanar D et al: Optimization of incubation conditions of Plasmodium falciparum antibody multiplex assays to measure IgG, IgG1-4, IgM and IgE using standard and customized reference pools for sero-epidemiological and vaccine studies. Malar J. 2018;17(1):219.

4. Bryan D, Silva N, Rigsby P, Dougall T, Corran P, Bowyer PW, Ho MM: The establishment of a WHO Reference Reagent for anti-malaria (Plasmodium falciparum) human serum. Malar J. 2017;16(1):314.

5. Alonso PL, Sacarlal J, Aponte JJ, Leach A, Macete E, Milman J, Mandomando I, Spiessens B, Guinovart C, Espasa M et al: Efficacy of the RTS,S/AS02A vaccine against Plasmodium falciparum infection and disease in young African children: randomised controlled trial. Lancet. 2004;364(9443):1411-1420.

6. Armbruster DA, Tillman MD, Hubbs LM: Limit of detection (LQD)/limit of quantitation (LOQ): comparison of the empirical and the statistical methods exemplified with GC-MS assays of abused drugs. Clin Chem. 1994;40(7 Pt 1):1233-1238.

7. Sanz H, Aponte JJ, Harezlak J, Dong Y, Ayestaran A, Nhabomba A, Mpina M, Maurin OR, Diez-Padrisa N, Aguilar R et al: drLumi: An open-source package to manage data, calibrate, and conduct quality control of multiplex bead-based immunoassays data analysis. PLoS One. 2017;12(11):e0187901.

8. Holm S: A simple sequentially rejective multiple test procedure. Scand J Stat. 1979;6:65-70

9. Benjamini Y, Hochberg Y: Controlling the false discovery rate: a practical and powerful approach to multiple testing. J R Stat Soc. 1995;B57:289-300.
